# New dengue virus inhibitors targeting NS3-NS5 interaction identified by *in silico* screening

**DOI:** 10.3389/fmicb.2025.1663404

**Published:** 2025-10-31

**Authors:** Giulio Nannetti, Beatrice Mercorelli, Alessandro Bazzacco, Nicolò Santi, Marta Celegato, Salvatore Ferla, Mattia Sturlese, Niklaas J. Buurma, Andrea Brancale, Arianna Loregian

**Affiliations:** ^1^Swansea University Medical School, Swansea University, Swansea, United Kingdom; ^2^School of Pharmacy and Pharmaceutical Sciences, Cardiff University, Cardiff, United Kingdom; ^3^Department of Molecular Medicine, University of Padua, Padua, Italy; ^4^Molecular Modeling Section, Department of Pharmaceutical and Pharmacological Sciences, University of Padua, Padua, Italy; ^5^School of Chemistry, Physical Organic Chemistry Centre, Cardiff University, Cardiff, United Kingdom; ^6^Department of Organic Chemistry, University of Chemistry and Technology, Prague, Czechia; ^7^Microbiology and Virology Unit, Padua University Hospital, Padua, Italy

**Keywords:** dengue virus inhibitors, NS3-NS5 interaction, dissociative inhibitors, protein-protein interaction, flavivirus, antiviral compounds

## Abstract

Dengue virus (DENV) poses a major public health concern as it is responsible for approximately 100 million human infections annually. Since no antiviral drugs are currently available to treat DENV infection, the development of effective therapeutic strategies is urgently needed. For anti-DENV drug discovery, the interaction between DENV NS3 and NS5 proteins represents an attractive target, as it is essential for viral replication and is highly conserved across all DENV serotypes. In this study, we report two distinct virtual screenings of commercially available drug-like compounds, which were performed to identify inhibitors of the NS3-NS5 interaction. Both screening approaches led to the identification of hit compounds that were able to reduce NS3-NS5 binding *in vitro* in a dose-dependent manner, as measured by an ELISA-based assay. Moreover, the hits inhibited the replication of DENV-2 at low micromolar and non-cytotoxic concentrations. Among these, hit 3 exhibited the highest selectivity index and showed antiviral activity against all four DENV serotypes. Biophysical studies indicated that hit 3 exerts its antiviral activity by directly binding to NS5. Hit 3 was then selected for structure-activity relationship studies, leading to the identification of structural analogues that retained anti-DENV activity through the disruption of NS3-NS5 interaction. Overall, this study reports the identification of a series of novel chemical scaffolds endowed with pan-dengue antiviral activity, representing a promising foundation for the development of new anti-DENV agents.

## 1 Introduction

Dengue virus (DENV) is the most prevalent mosquito-borne viral pathogen in tropical and subtropical regions. DENV belongs to the genus *Orthoflavivirus* within the *Flaviviridae* family and comprises four different serotypes (DENV-1 to DENV-4), classified according to their antigenic properties. All DENV serotypes are responsible for widespread human outbreaks of dengue, which ranges from asymptomatic or mild self-limited febrile illness to life-threatening severe dengue fever, potentially associated with hemorrhagic complications and/or shock syndrome (Guzman et al., 2016). Approximately 100 million DENV infections occur annually, leading to about 4 million hospitalizations and almost 30,000 deaths worldwide each year (Stanaway et al., 2016; Cattarino et al., 2020; Zhang et al., 2025). Dengue is currently endemic in almost 100 countries and about half of the world’s population is at high risk of DENV infection (Nakase et al., 2024). In addition, due to environmental and economic factors (i.e., climate change, intercontinental travel, and increasing urbanization), both the geographical distribution and incidence of DENV infections have been increasing and are expected to further expand into new regions in the coming decades (Nakase et al., 2024; Messina et al., 2019). Indeed, DENV has recently been responsible for epidemic outbreaks in Europe and other non-endemic areas (Frasca et al., 2024; Farooq et al., 2025).

Despite this growing public health threat, the development of safe and effective strategies to prevent and treat DENV infections remains incomplete. No antiviral agents to manage DENV infections are yet licensed and the clinical benefit of the available tetravalent DENV vaccines, Dengvaxia and Qdenga, are limited by safety concerns and inconsistent long-term efficacy across the four DENV serotypes (Halstead, 2024). In fact, Dengvaxia has been shown to increase the risk of hospitalization and development of severe dengue in DENV-seronegative vaccinated individuals (Sridhar et al., 2018). Thus, its current use is restricted only to individuals aged 6–45 years with a confirmed history of DENV infection (European Medicines Agency, 2018). Furthermore, Qdenga vaccine has been demonstrated to provide long-term protection primarily against DENV-1 and DENV-2, with low or uncertain efficacy toward the other DENV serotypes, thereby limiting its application in clinical contexts (Tricou et al., 2024). Waiting for a cross-protective vaccine, the development of antiviral drugs against DENV is a priority for global health management.

In pursuing such a goal, the multi-functional NS3 and NS5 proteins of DENV provide attractive antiviral targets, as they are the only enzymatic components of the flavivirus replication complex and play a key role in viral RNA replication. NS3 and NS5 are the most conserved proteins across DENV serotypes and both possess two distinct functional domains separated by a flexible linker. NS3 comprises an N-terminal chymotrypsin-like protease domain that is activated by the NS2B co-factor and a C-terminal helicase (hel) domain (Luo et al., 2008). On the other hand, NS5 consists of an N-terminal methyl-transferase (MTase) domain and a C-terminal RNA-dependent RNA polymerase (RdRp) domain (Egloff et al., 2002; Yap et al., 2007). During viral RNA replication, NS5 RdRp synthesizes the single-stranded (ss) negative-sense RNA (-RNA) starting from the ss positive-sense genomic RNA (+ RNA), leading to the formation of an intermediate double-stranded (ds) RNA. Subsequently, the NS3 hel unwinds the dsRNA into two RNA strands, allowing the ss -RNA to serve as a template for replicating the genomic + RNA. The interaction between NS3 hel and NS5 RdRp is critical for coordinating and stimulating their enzymatic activities during viral RNA replication in DENV and other flaviviruses to align their RNA binding channels, as shown in infected cells and by recent *in vitro* structural studies (Kapoor et al., 1995; Tay et al., 2015; Xu et al., 2019; Osawa et al., 2023; Brand et al., 2024).

The NS3-NS5 interaction interface has been mapped to the C-terminal part of NS3 hel (residues 566–585, using the DENV-2 numbering) and the N-terminal region of NS5 RdRp (residues 320–341; Tay et al., 2015). Mutagenesis studies have also revealed two residues at the NS3-NS5 binding interface, i.e., Asn570 in NS3 and Lys330 in NS5 (DENV-2 numbering), as crucial for the interaction (Tay et al., 2015; Zou et al., 2011). Indeed, a single amino acid substitution by mutagenesis of both residues to alanine (i.e., N570A in NS3 and K330A in NS5) resulted in the abolishment of DENV replication through the disruption of NS3-NS5 interaction (Tay et al., 2015; Zou et al., 2011). Interestingly, both residues, i.e., Asn570 in NS3 and Lys330 in NS5, are located within two cavities at the interaction interface, previously referred to as site A on NS3 and cavity B on NS5, formed by highly conserved residues in DENV and other related flaviviruses and proposed as druggable pockets (Zou et al., 2011; Malet et al., 2008; Munawar et al., 2018). These insights suggested that the disruption of NS3-NS5 interaction by rationally designed small molecules could lead to the identification of potential broad-spectrum anti-DENV agents.

Targeting critical protein–protein interactions (PPIs) among nonstructural (NS) proteins within the flavivirus replication complex has emerged as a promising antiviral strategy against DENV infection (Chauhan et al., 2024). Indeed, recent studies have led to the development of several PPI inhibitors with demonstrated anti-DENV efficacy in both *in vitro* and *in vivo* models (Kaptein et al., 2021; Moquin et al., 2021; Goethals et al., 2023; Celegato et al., 2023). Among these, two potent compounds targeting the binding between NS3 and NS4B proteins, NITD-688 and mosnodenvir (formerly known as JNJ-1802), have advanced to phase II clinical trials (Goethals et al., 2023; Wang et al., 2025), highlighting the potential clinical translation of compounds targeting essential PPIs between DENV nonstructural proteins. Similarly, previous efforts targeting the NS3-NS5 interaction have identified inhibitors with demonstrated anti-DENV activity (Celegato et al., 2023; Vincetti et al., 2015; Cannalire et al., 2020; Yang et al., 2022), further validating this PPI as a viable antiviral target.

Building on this, using two available crystal structures of DENV NS3 and NS5, we performed two distinct, complementary structure-based virtual screenings (SBVS) of over 3 million commercially available drug-like molecules to identify new potential anti-DENV compounds targeting the NS3-NS5 interaction. Among the selected virtual hits, some compounds were able to disrupt the protein-protein interaction between NS3 and NS5 *in vitro* and inhibited the replication of DENV in infected cells. Starting from the hit exhibiting the highest Selectivity Index (SI), a series of structural analogues were synthesized or purchased, and several compounds were shown to interfere with the NS3-NS5 interaction. Overall, this study led to the identification of novel chemical scaffolds endowed with NS3-NS5 inhibitory activity, which represent a promising starting point in the development of DENV inhibitors of this novel class.

## 2 Materials and methods

### 2.1 Molecular modeling

All the molecular modeling studies were performed on an Intel(R) Core(TM) i7-6700 CPU @ 3.40 GHz processor, running Ubuntu 18.04.03 LTS. Molecular Operating Environment (MOE) 2015.1066, Maestro (Schrödinger Release 2017–1), LeadIT FlexX (version 2.1.8), and PLANTS were used as computational software.

#### 2.1.1 Homology modeling

The homology models of full-length DENV-2 NS3 and NS5 proteins were built using the MOE Homology Model tool. The available crystal structures of NS3 [Protein Data Bank (PDB) code: 2WHX; Luo et al., 2010] and NS5 (PDB code: 5K5M implemented with that of PBD ID: 5JJS to complete the gaps; Lim et al., 2016) were downloaded from the PDB and used as templates for the respective models. FASTA sequences of NS3 and NS5 proteins of DENV-2 were aligned to the respective template samples and the models were built using the Amber12: EHT force field. The automatic disulfide bond detection and the C-/T- terminal outgap modeling were disabled. After a mild refinement to relieve the steric strains, ten intermediate models were generated and ranked according to the Coulomb and Generalized Born/Volume Integral (GB/VI) interaction energies (Labute, 2008). Among them, the top scoring of both proteins were chosen as the final models. The structures of the final homology models were refined using the MOE structure preparation tool and the φ and ψ angles were analyzed with the Ramachandran plot and corrected accordingly, if required. Finally, both models were validated using the SAVES v5.0 server^1^.

#### 2.1.2 Ligand preparation

A selection of commercially available compounds was downloaded from Specs^2^, ChemDiv^3^, Enamine^4^, and Life Chemicals^5^ websites in.sdf format and prepared with the LigPrep tool of the Schrödinger Maestro. The databases were then processed by generating all feasible tautomers for each ligand and assigning all possible protonation states at pH 7.0 ± 2.0 by Epik, using all atom OPLS force field.

#### 2.1.3 Structure-based virtual screening (SBVS)

**FIGURE 1:**
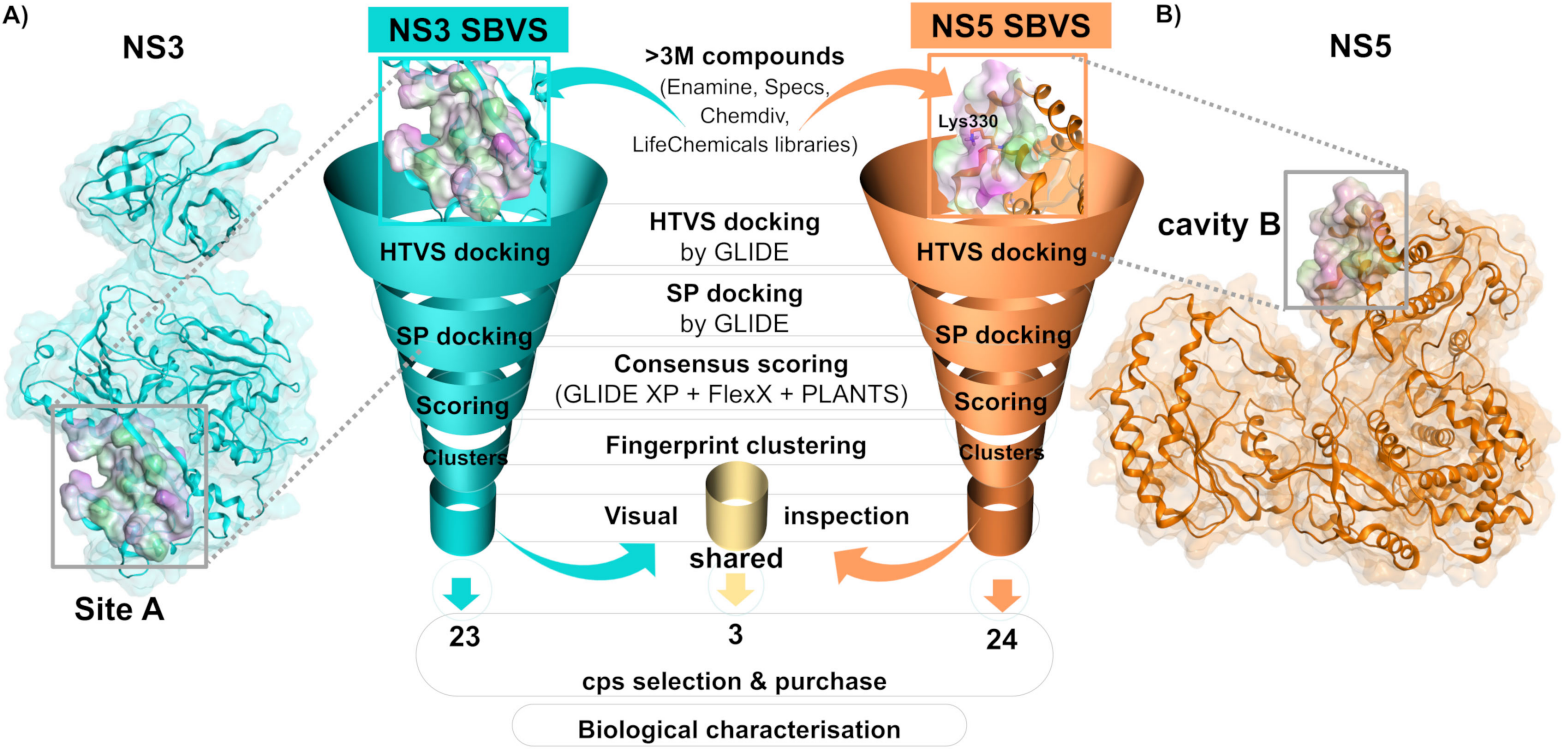
Overview of the structure-based virtual screenings (SBVS) approaches. The database of over 3 million drug-like small molecules was docked into site A **(A)** or cavity B **(B)** of NS3 and NS5, respectively, of DENV-2. A series of increasingly more accurate molecular docking filters were applied to both SBVS approaches. The final docking poses of top-ranked compounds of each SBVS were then rescored using different scoring functions, clustered and visually inspected. Selected hits were purchased and subjected to biological characterization.

The presence of potentially druggable cavities at the interface of NS3-NS5 interaction was inspected on the validated homology models of NS3 and NS5 by the means of the MOE Site Finder tool. The identified cavities on NS3 and NS5 (corresponding to those previously referred to as site A and cavity B, respectively; Munawar et al., 2018; Malet et al., 2008) were subjected to two distinct SBVS (represented in Figure 1).

The validated homology models of DENV-2 NS3 and NS5 were first prepared using the Schrödinger Protein Preparation Wizard tool by: (i) adding missing hydrogens, (ii) determining protonation states, and (iii) performing a restrained minimization of hydrogen atoms, applying the OPLS_2005 force field. After that, a 20 Å-sized docking grid was generated for each protein and centered on the residue Lys330 of NS5 for the NS5 SBVS and on the center of the site A of NS3 for the NS3 SBVS. The size of the ligand diameter midpoint boxes was set at 10 Å. The two generated grids were used to perform two independent SBVS on NS3 and NS5 using the Glide virtual screening workflow of the Schrödinger suite (Friesner et al., 2004). Briefly, the database of prepared drug-like ligands was first docked into both the selected sites on NS3 and NS5 using Glide HTVS precision. The compounds that fell within the top 10% of ranked HTVS outputs were then re-docked by Glide SP (standard precision), generating the three best poses for each selected ligand. The SP docking poses of each SBVS were then rescored using the scoring functions of three different software: Glide XP, PLANTS, and LeadIT-FlexX (Friesner et al., 2004; Korb et al., 2006; Kramer et al., 1997). After applying an internal consensus score procedure, only compounds falling in the top 25% score ranking of all the three scoring functions were selected. The selected compounds from both SBVS approaches were then clustered based on the MACCS algorithm (setting 75% as similarity and overlap values) by MOE. Compounds having i) a molecular weight < 250 or > 500, (ii) logP values > 5 or < 1, or (iii) structural moieties associated with a high risk of toxicity were discarded. Finally, the binding poses of the selected compounds within the respective binding sites of NS3 or NS5 were visually inspected to select the hits from each SBVS, along with any shared compounds identified in both screenings. The selected hits were then purchased and subjected to biological assays. All the hits were also examined for potential Pan Assay INterference compoundS (PAINS) and colloid aggregator risks^6^ (Sterling and Irwin, 2015). All compounds passed the filters, except for hit 50, which was flagged as a potential PAINS. Hit 50 confirmed activity across multiple assays using distinct detection methods, including tests designed to rule out colloidal aggregation, as described below, but was ultimately excluded from the final selection.

### 2.2 Compounds and peptides

Selected hits were purchased from SPECS, Enamine, and ChemDiv. Some hit 3 analogues - compounds 51–54 - were obtained from Enamine, while the remaining analogues were synthesized in-house, with procedures detailed in the Supplementary Information. All compounds, either purchased or synthesized, were > 95% pure and were dissolved in dimethyl sulfoxide (DMSO, Merck Life Sciences, Darmstadt, Germany).

Synthetic NS3_86–100_ and NS3_566–585_ peptides corresponding to residues 86–100 and 566–585 of the NS3 protein sequence of DENV-2, respectively, were purchased from GenScript (GenScript Biotech, Piscataway, NJ, United States) at a purity higher than 95%. Both peptides were dissolved in phosphate-buffered saline (PBS).

### 2.3 Cells and viruses

African Green Monkey Vero cells were purchased from American Type Culture Collection (ATCC, CCL-81) and were cultured in Dulbecco’s modified Eagle medium (DMEM) supplemented with 10% fetal bovine serum (FBS, Life Technologies) in the presence of 100 U/mL penicillin and 100 μg/mL streptomycin (Life Technologies) and maintained at 37 °C in a humidified atmosphere supplemented with 5% CO_2_.

DENV-1 (TH-Sman strain) was purchased from ATCC, while DENV-2 (NGC strain), DENV-3 (H-87 strain), and DENV-4 (H-241 strain) were purchased from NCPV (Public Health England, United Kingdom). All work with infectious DENV was performed in a biosafety level 3 (BSL3) laboratory according to the safety guidelines of the Department of Molecular Medicine (University of Padua, Italy) committee on microbiological safety.

### 2.4 Plasmids

To obtain the pRSET-NS2B18-NS3 full length (f.l.) plasmid, the sequence encoding NS3 of DENV-2 fused to the 49-66 residues of NS2B by a glycine linker (Luo et al., 2008) was custom synthesized and inserted into the *Bam*HI/*Hin*dIII sites of pRSET plasmid downstream of the 6His tag (Thermo Fisher Scientific, Waltham, MA, United States). To obtain the pGEX4T-1-NS3_172–618_ plasmid, the coding sequence of NS3 hel domain (amino acids 172-618) was amplified from pRSET-NS2B18-NS3 using the forward primer 5′-AGACTAGGATCCATTGAAGACAATCCAGAG-3′ and the reverse primer 5′-ATAACTGTCGACTTACTTTCTTCCAGCTG CG-3′. The underlined bases indicate the *Bam*HI and *Sal*I restriction sites, respectively, used for cloning. The amplified sequence was then cloned into *Bam*HI/*Sal*I sites of pGEX-4T-1 (GE Healthcare Life Sciences, Piscataway, NJ, United States), downstream of the GST coding sequence.

To generate the pET28a–NS5_270–900_ plasmid, the coding region of the NS5 RdRp domain (residues 270-900) of the DENV-2 NGC strain was obtained by PCR using the forward primer 5′-TTACACGGATCCAGCGGAACCCGCAACATCGGA-3′ and the reverse primer 5′-TTAGAGGTCGACCTACCACAGGACTCCT GC-3′. The underlined bases indicate the *Bam*HI and *Sal*I restriction sites, respectively, used for cloning. The amplified sequence was cloned into *Bam*HI/*Sal*I sites of pET28a (Novagen, Darmstadt, Germany), downstream of the 6His tag.

The correctness of the plasmid constructs together with the lack of undesired mutations was confirmed by DNA sequencing (Eurofins Genomics, Ebersberg, Germany).

### 2.5 Protein expression and purification

*Escherichia coli*-expressed, purified 6His-tagged proteins were obtained as previously described (Li Pira et al., 2004; Sinigalia et al., 2012), with some modifications. To obtain the 6His–NS2B(CF18)-gly-NS3 full-length (NS3 f.l.) and 6His–NS5 RdRp_270–900_ proteins, the pRSET-NS2B18-NS3 and pET28a–NS5_270–900_ plasmids, respectively, were transformed into *E. coli* BL21-CodonPlus-RIPL competent cells (Stratagene, Bellingham, WA, United States) and grown at 37 °C in Luria Bertani (LB) medium containing the respective selection antibiotics. After reaching an optical density at 600 nm (OD_600_) of 0.6, the expression of 6His–NS5 RdRp_270–900_ was induced by adding 0.2 mM isopropylβ-d-1-thiogalactopyranoside (IPTG, Merck Life Sciences) overnight at 16 °C, while the induction of 6His–NS2B(CF18)-gly-NS3 expression was performed overnight at 18 °C by adding 0.4 mM IPTG. Induced cells were then collected by centrifugation at 5,000 *g* for 10 min at 4 °C and lysed by sonication after 30-min incubation at 4 °C in buffer A [20 mM sodium phosphate, pH 7.4, 500 mM NaCl, 5 mM β-mercaptoethanol, 10% (v/v) glycerol]) supplemented with 1 mg/mL lysozyme (Thermo Fisher Scientific), 25 U/mL Pierce Universal Nuclease (Thermo Fisher Scientific), EDTA-free protease inhibitor Cocktail (Roche, Waltham, MA, United States) and 10 mM imidazole (Merck Life Science). The lysates were centrifuged at 20,000 *g* for 30 min at 4 °C and the soluble fractions were filtered through a 0.45-μm membrane filter (Millipore, Bedford, MA, United States) and loaded onto pre-equilibrated HisTrap HP columns (GE Healthcare Life Sciences) with buffer A. The columns were then thoroughly washed with buffer A supplemented with 30 mM imidazole and the His-tagged proteins were eluted by 300 mM imidazole in the same buffer. Subsequently, the buffer of the eluted fractions was exchanged using a Vivaspin 20 Ultra centrifugal unit (30 kDa cut-off, Merck Life Science) and the proteins were further purified using a gel-filtration column (SuperdexTM 200 HR 10/300 column, GE Healthcare Life Sciences) in 20 mM Tris-HCl pH 7.4, 300 mM NaCl, 5% glycerol, and 1 mM dithiothreitol (DTT). Fractions were pooled, concentrated using a Vivaspin 20 Ultra centrifugal unit in the storage buffer (20 mM Tris-HCl, pH 7.4, 300 mM NaCl, 1 mM DTT, 20% glycerol) and stored in aliquots at −80 °C.

The GST and GST-NS3_172–618_ proteins were expressed from *E. coli* BL21-CodonPlus-RIPL competent cells transformed with pGEX-4T-1 and pGEX-4T-1-NS3_172–618_ plasmids, respectively. Transformed bacteria were grown in LB medium plus 100 μg/mL ampicillin until the OD_600_ value reached 0.6 and the expression of both GST proteins was then induced by the addition of 0.5 mM IPTG. After overnight incubation, cells were pelleted and resuspended with buffer B (20 mM Tris-HCl, pH 7.4, 150 mM NaCl, 2 mM DTT, 10% glycerol) supplemented with 1 mg/mL lysozyme, 25 U/mL Pierce Universal Nuclease (Thermo Fisher Scientific) and protease inhibitor Cocktail (Thermo Fisher Scientific). Resuspended cells were incubated for 30 min at 4 °C, sonicated on ice and centrifuged at 20,000 *g* for 30 min at 4 °C. The supernatants were further clarified through a 0.45-μm filter and applied onto GSTrap FF columns (GE Healthcare Life Sciences), which had been equilibrated with buffer B. After washing both columns with buffer B, the GST proteins were eluted with 20 mM reduced glutathione (Merck Life Science) added to the same buffer. Each GST protein was finally dialyzed at 4 °C against the storage buffer, aliquoted and stored at −80 °C. Protein purity was estimated by sodium dodecyl sulfate-polyacrylamide gel analysis.

### 2.6 ELISA-based NS3-NS5 interaction assay

Nunc Maxisorp microplates (Thermo Fisher Scientific) were coated with 400 ng/well of 6His-NS5_270–900_, or GST protein as a control, in PBS for 3 h at 37 °C and then blocked with 5% (wt/v) bovine serum albumin (BSA, Merck Life Science) in PBS overnight at 4 °C. Blocked wells were incubated at room temperature (RT) for 2 h with increasing amounts of NS3 f.l. or GST–NS3_172–618_. 6His-NS5_270–900_-coated plates were also incubated with increasing concentration of GST, as a control. After washing with PBS containing 0.3% Tween 20 (Fisher Scientific, Waltham, MA, United States), the NS3-NS5 interaction was detected by incubating the plates with the anti-NS3 monoclonal antibody (GT2811 clone, Genetex, Irvine, CA, United States) diluted 1:6,000 in PBS + 2% (v/v) FBS (Thermo Fisher Scientific) for 1 h at RT. The unbound primary antibody was removed by further washes with PBS + 0.3% Tween 20 and the secondary goat anti-mouse HRP-conjugated antibody (GenScript Biotech) at a dilution of 1:8,000 in PBS + 2% FBS was then added. After incubation for 1 h at RT and following washes, the colorimetric substrate 3,3′,5,5′-tetramethylbenzidine (TMB, SeraCare Life Sciences, Milford, MA, United States) was added to each well and absorbance was read at 450 nm by an ELISA plate reader (Tecan Infinite 200 PRO, Mannedorf, Switzerland).

To test the inhibitory effect of compounds on NS3-NS5 interaction, 6His-NS5_270–900_-coated wells were incubated with a fixed amount of NS3 f.l. (200 ng) along with increasing concentrations of test compounds or DMSO as a control. To exclude non-specific inhibition of compounds due to colloidal aggregation, the inhibitory activities of compounds were tested in the presence or the absence of 0.002% Triton X-100 (Fisher Scientific), a condition which reverses and prevents the colloidal formation of organic molecules (McGovern et al., 2003). All the compound concentrations were tested at least in duplicate.

### 2.7 Cytotoxicity assay

The cytotoxicity of the test compounds was assessed in Vero cells by the 3-(4,5-dimethylthiazol-2-yl)-2,5-diphenyl tetrazolium bromide (MTT, Merck Life Science) method. Vero cells were cultured in 96-well plates at a density of 1 × 10^4^ cells per well and incubated at 37 °C overnight. The following day, cells were incubated with different concentrations of test compounds or DMSO for 72 h at 37 °C. All the compound concentrations were tested at least in duplicate. Cell viability was then determined as previously reported (Mercorelli et al., 2016).

### 2.8 Plaque reduction assays

Plaque reduction assays were performed as previously described (Pismataro et al., 2021; Celegato et al., 2023), with some modifications. Vero cells were seeded at 3 × 10^5^ cells/well in 12-well plates and incubated for 24 h at 37 °C. The next day, cells were washed and then infected with 40 Plaque Forming Units (PFU) per well of DENV-2 NGC strain. Following 1 h of incubation at 37 °C, cells were washed and then incubated with DMEM containing 2% FBS, 1.2% Avicel microcrystalline cellulose (FMC BioPolymer Philadelphia, PA, United States), and different concentrations of test compounds or DMSO as a control. In both compound-treated and control samples, DMSO was maintained at 0.1% to prevent cytotoxic effects. Each compound concentration was tested at least in duplicate. After 7 days of infection, cells were fixed with 4% formaldehyde solution (Merck Life Science) and stained. The mean plaque number of compound-treated samples was compared to that of the DMSO-treated control, which was set as 100%.

### 2.9 Viral RNA quantification

For viral RNA reduction assays with DENV serotypes 1 to 4, Vero cells were seeded at a density of 1 × 10^5^ cells per well in 24-well plates. The next day, cells were infected at 37 °C with the different viruses in serum-free DMEM at the multiplicity of infection (MOI) of 0.001 and plates were rocked every 15 min. At the end of incubation, the viral inoculum was removed, and medium with 2% FBS containing various concentrations of test compounds was added. Viral RNA was isolated from the culture supernatant at 72 h p.i., using the PureLink Viral RNA/DNA Mini kit (Invitrogen, Waltham, MA, United States), according to the manufacturer’s instructions. Viral RNA was quantified using the One-Step RT-PCR kit Dengue Virus subtypes 1, 2, 3 and 4 PCRmax (Cole Parmer Instrument, Saint Neots, United Kingdom), according to the manufacturer’s instructions, on a 7,000 Real-time PCR platform (Applied Biosystems, Waltham, MA, United States).

### 2.10 Microscale thermophoresis (MST) studies

To obtain the purified helicase portion of NS3 (6His-NS3_177–618_) and the main protease of SARS-CoV-2 (6His-M^pro^) used for the MST analysis, we followed previously described protocols (Celegato et al., 2023; Mercorelli et al., 2022). The 6His–NS5 RdRp_270–900_ protein was expressed and purified as described above. For MST studies, 6His-NS5_270–900_, 6His-NS3_177–618_, and 6His-M^pro^ were fluorescently labeled using NanoTemper Protein labeling kit RED-NHS 2*^nd^* generation (MO-L011, NanoTemper Technologies GmbH, Munich, Germany). Protein and dye concentrations were determined by absorption spectroscopy using NanoDrop™ One spectrophotometer (ThermoFisher Scientific), considering, at 280 nm, ε = 171,350 M^–1^cm^1^ for recombinant NS5, ε = 66,140 M^–1^cm^1^ for NS3, ε = 32,890 M^–1^cm^1^ for M^pro^, respectively. The ε = 195,000 M^1^cm^1^ at 650 nm was considered for the RED-NHS fluorophore, with a correction factor of 0.04. The degree of labeling (DOL) was calculated according to the manufacturer’s instructions, yielding a value of 0.63 for NHS-NS5, 0.98 for NHS-NS3, and 0.60 for NHS-M^pro^, respectively. Binding experiments were performed in MST buffer (20 mM Tris-HCl, pH 8.0, 140 mM NaCl, 1 mM EDTA, 0.01% Tween 20, 1 mM DTT) previously reported (Desantis et al., 2024). For NS3-NS5 binding affinity experiments, 100 nM NHS-NS5_270–900_ was incubated with 16 serially diluted (1:2) concentrations of unlabeled 6His-NS3_177–618_ starting from 35 μM. For binding affinity experiments with hit compound 3, 100 nM NHS-NS5_270–900_ and 50 nM NHS-NS3_177–618_ were incubated with 16 different concentrations of compound 3 serially diluted 1:2 starting from 250 μM. As a control for specificity, binding check experiments were performed with 125 μM compound 3 and SARS-CoV-2 NHS-M^pro^ (50 nM). All samples were incubated for 15 min at room temperature in the dark and then loaded into glass capillaries (Monolith premium Capillaries, #MO-K025, NanoTemper). Thermophoresis analysis was performed using NanoTemper Monolith X (at Medium MST laser power). To detect the binding of NS3-hel or compound 3 to NHS-NS5, 20% and 40% LED power were used, respectively. To detect the binding of compound 3 to NHS-NS3 or NHS-M^pro^, 60% LED power was used. In all experiments, signal quality was monitored by the Monolith device to detect possible ligand auto-fluorescence, aggregation, or target protein adsorption to capillaries. Experiments were performed in triplicates and analyzed with the MO. Affinity Analysis software (NanoTemper).

### 2.11 Statistical analysis

Statistical analysis was performed using GraphPad Prism version 8.0 (GraphPad Software, San Diego, CA, United States). Data are shown as the means ± standard deviations (SD) of at least two experiments in duplicate. The dose-response curves and IC_50_, EC_50_, and CC_50_ values were calculated by non-linear regression curve fitting [(inhibitor) versus normalized response with variable slope] using GraphPad Prism 8.0 software.

## 3 Results

### 3.1 Hits identification by NS3- and NS5-structure-based virtual screenings

To identify potential inhibitors of the DENV NS3-NS5 interaction, two parallel and independent structure-based virtual screenings (SBVS) of over 3 million commercially available drug-like compounds were conducted on two pockets located at the interface on both NS3 and NS5, as shown in Figure 1. Since the NS3 and NS5 structures of the DENV strain used in our ELISA assay were not solved at the time of the screening, homology models of the DENV-2 NGC strain were built using the available structures of DENV NS3 and NS5 (Luo et al., 2010; Lim et al., 2016). The validated DENV-2 NS3 and NS5 homology models were then inspected to find druggable pockets at the NS3-NS5 interaction interface. Two potential druggable cavities encompassing two crucial residues for the NS3-NS5 interaction, i.e., Asn570 of NS3 and Lys330 of NS5 (Tay et al., 2015; Zou et al., 2011), corresponding to the aforenamed site A on NS3 and cavity B on NS5 (Malet et al., 2008; Munawar et al., 2018), were identified on both proteins and chosen as target sites. Successively, more than 3 million compounds from the Specs, Enamine, LifeChem, and ChemDiv libraries were virtually screened with an increasing accuracy level first using Glide-HTVS and Glide-SP scoring functions against both the selected target cavities. The resulting SP docking poses of the 300,000 top-ranked molecules on each binding site were then re-scored using Glide XP, LeadIT-FlexX, and PLANTS software (Friesner et al., 2004; Korb et al., 2006; Kramer et al., 1997). Compounds were then ranked using an internal consensus scoring procedure based on all three rescoring functions (Ferla et al., 2018). Following visual inspection of the docking poses of the 500 top-ranked compounds at their respective target sites, 23 virtual hits were selected from the NS3 SBVS and 24 from the NS5 SBVS. Additionally, 3 top-ranked compounds identified by both screenings were included. In total, 50 compounds were selected (Supplementary Table 1), purchased, and subjected to the biological evaluation.

### 3.2 Development of the NS3-NS5 ELISA-based interaction assay

**FIGURE 2:**
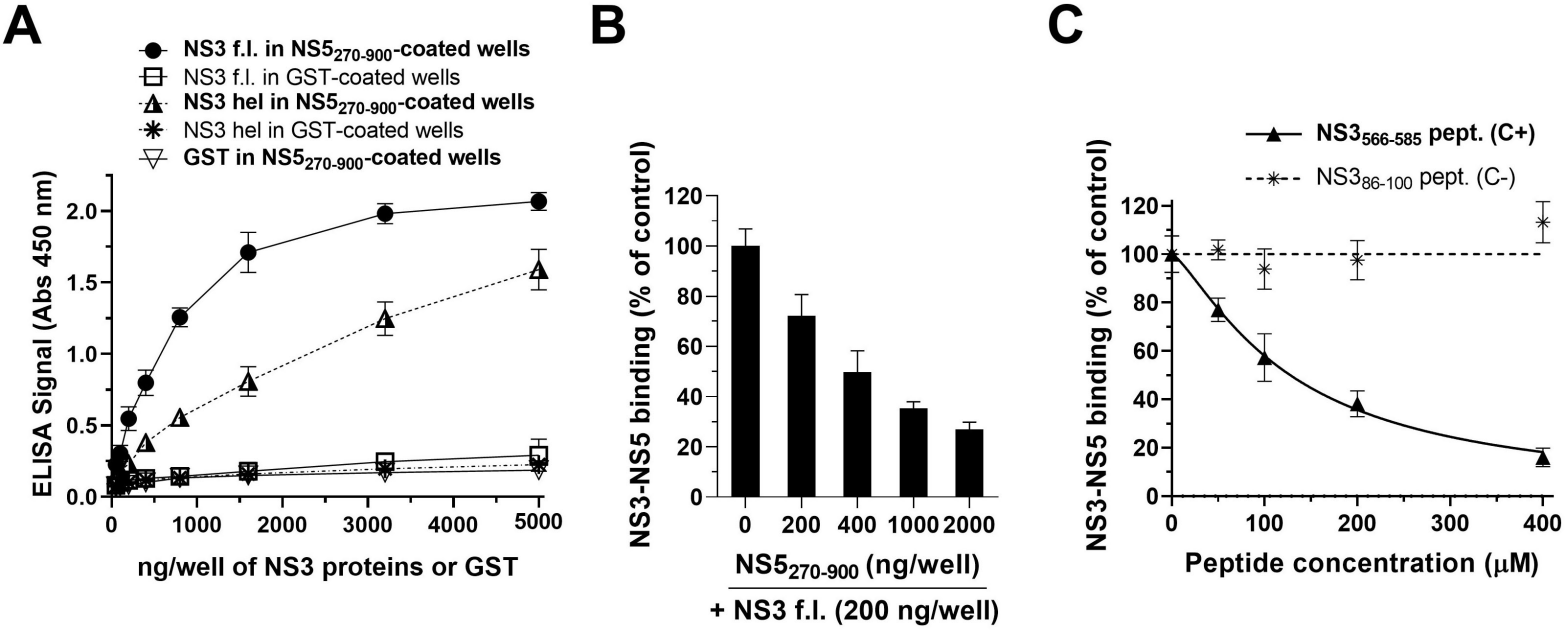
NS3-NS5 ELISA interaction assay. **(A)** Wells coated with 400 ng of 6His–NS5_270–900_ or GST as a control, were incubated with increasing amounts of NS3 f.l. or GST–NS3_172–618_. Increasing concentrations with GST were also added to 6His–NS5_270–900_-coated wells, as a further control. The NS3-NS5 binding was detected by the addition of an anti-NS3 monoclonal antibody, followed by an HRP-conjugated secondary antibody. The data shown represent the mean ± SD of at least three independent experiments in duplicate. Increasing amounts of free 6His–NS5_270–900_ **(B)** and either NS3_566–585_ or NS3_86–100_ peptides **(C)** were added along with a fixed amount (200 ng) of NS3 f.l. to wells coated with 400 ng of 6His–NS5_270–900_. Residual NS3-NS5 binding was detected as reported above. Data shown represent the mean ± SD of *n* = 3 independent experiments in duplicate.

In order to evaluate the inhibitory effects of the 50 selected hits from the NS3 and NS5 SBVS, an ELISA-based assay was developed to detect and measure the interaction between NS3 and NS5, similar to assays we previously carried out for other viral protein-protein interactions (Loregian et al., 2003; Muratore et al., 2012; Celegato et al., 2020; Bertagnin et al., 2023). The 6His-NS5_270–900_ protein was used for the coating of microtiter plates and then incubated with GST-NS3_172–618_, NS3 full-length (f.l.) conjugated to a 18-residue segment of its NS2B cofactor (hereafter NS3 f.l.), or GST as a control. The NS3-NS5 interaction was successively detected by a monoclonal anti-NS3 antibody, targeting a region of NS3 hel that is distant from the NS5-binding site, followed by a secondary antibody coupled with HRP. As reported in Figure 2A, the incubation of the fixed amount of NS5_270–900_ with increasing amounts of either NS3 f.l. or GST-NS3_172–618_ (NS3 hel) led to a dose-dependent increase in the interaction signal. On the contrary, no specific binding was detected when GST was added as the interacting protein in NS5-coated plates (Figure 2A). A similar result was obtained when GST was used as a negative control to coat the plates, which were then incubated with NS3 (Figure 2A), thus confirming the specificity of the assay. Consistent with data previously obtained by a similar ELISA (Tay et al., 2015), the addition of NS3 f.l. to NS5-coated plates resulted in a higher signal compared to that obtained by adding an equal amount of NS3 hel (Figure 2A).

To demonstrate the ability of the ELISA to detect inhibition of the NS3-NS5 interaction, a series of competitive binding assays were performed. First, microplates coated with 6His-NS5_270–900_ were incubated with a fixed concentration of NS3 f.l. along with an increasing amount of free 6His-NS5_270–900_. As shown in Figure 2B, the addition of an increasing amount of free NS5-RdRp resulted in a dramatic reduction of the ELISA signal as a consequence of the competition with the coated NS5 in interacting with NS3. To further validate this assay, we then assessed the inhibitory effect of a known inhibitor of the NS5-NS5 interaction, i.e., the synthetic peptide NS3_566–585_, corresponding to the NS5-interacting region of NS3 (Tay et al., 2015). The NS3_86–100_ peptide, corresponding to a region of NS3 that is not involved in the NS5 binding, was also tested as a negative control. As expected, the NS3_566–585_ peptide inhibited the NS3-NS5 interaction in a dose-dependent manner, exhibiting an IC_50_ value of 127.8 μM (Figure 2C), which is in agreement with previously reported data (Tay et al., 2015). On the contrary, no inhibitory effect was observed with the inactive NS3_86–100_ peptide up to 480 μM (Figure 2C), as previously published (Tay et al., 2015). Overall, these data showed that this ELISA-based NS3-NS5 interaction assay is able to specifically measure the interaction between DENV NS3 and NS5 proteins and detect specific inhibitory effects on their binding.

### 3.3 Hit compounds effects on NS3-NS5 interaction *in vitro*

Once validated, the ELISA interaction assay was used to evaluate the ability of the virtual hits to interfere with the NS3-NS5 binding *in vitro*. All 50 compounds were first tested at the fixed concentration of 100 μM and their activity was compared to that of control samples incubated with an equal volume of DMSO. The NS3_566–585_ and NS3_86–100_ peptides were also included in each experiment as a positive and a negative control of inhibition, respectively. Among the tested compounds, 10 hits (i.e., 1, 3, 4, 6, 15, 29, 30, 44, 47, and 50) exhibited ≥ 50% inhibition of NS3-NS5 interaction at 100 μM (Supplementary Figure 1). On the contrary, the remaining compounds showed weaker or no inhibitory effect in ELISA (Supplementary Figure 1).

**FIGURE 3:**
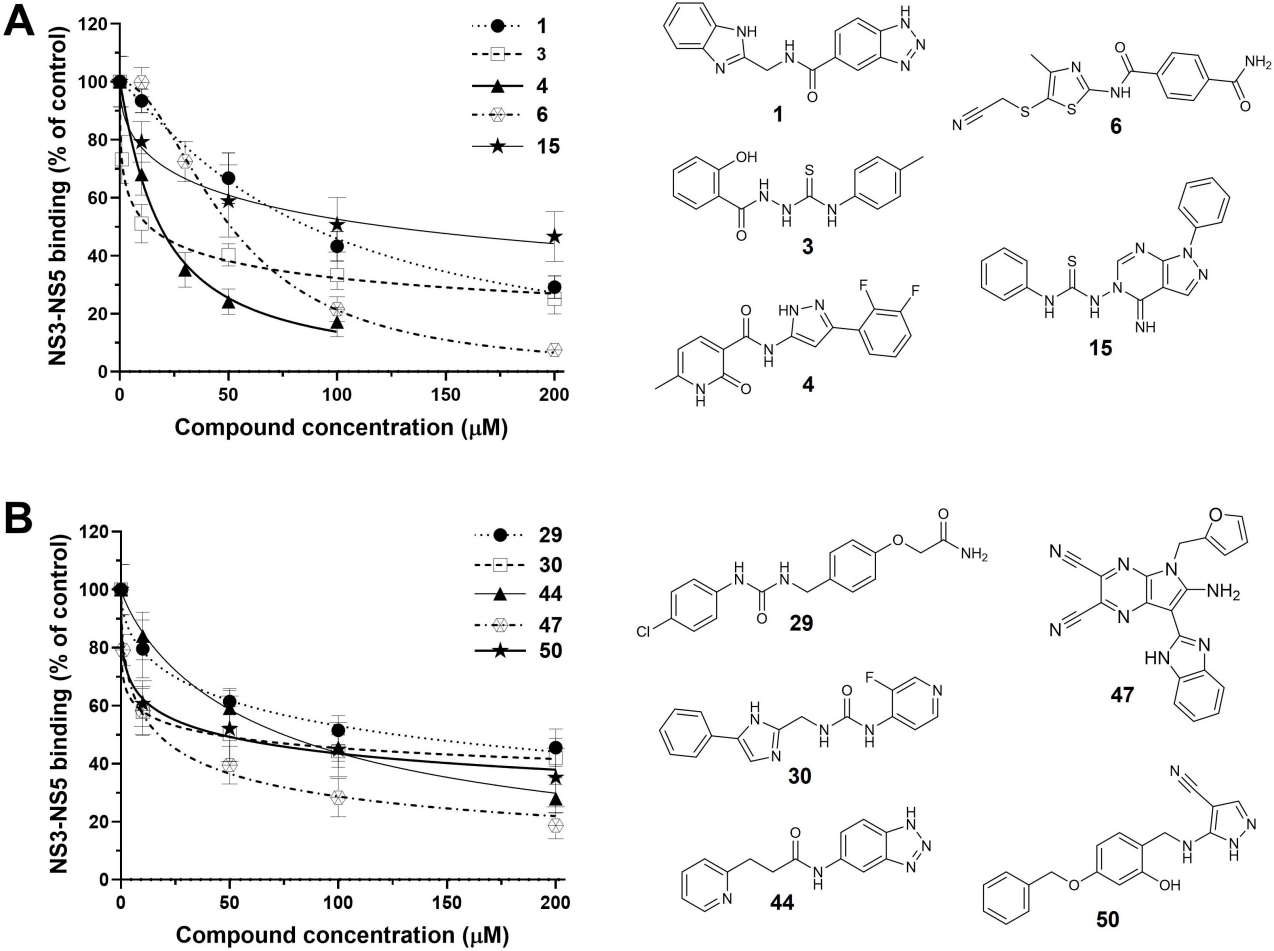
Concentration-dependent inhibition of NS3-NS5 binding by the selected hits in the ELISA NS3-NS5 interaction assay. Increasing concentrations of active hits 1, 3, 4, 6, and 15 **(A)** and 29, 30, 44, 47, and 50 **(B)** were added along with a fixed amount of NS3 f.l. (200 ng) to wells pre-coated with 6His–NS5_270–900_. Data shown represent the mean ± SD of *n* ≥ 3 independent experiments in duplicate.

All the active hits, together with the reference peptides, were then evaluated in a dose-response analysis by ELISA at different concentrations. As highlighted in Figures 3A, B along with their chemical structures, all the tested compounds showed a dose-dependent inhibition of the NS3-NS5 interaction. Hits 4 (from the NS3 SBVS), 47 (from the NS5 SBVS), and 3 (identified in both screenings) emerged as the most potent inhibitors of the series, exhibiting IC_50_ values ranging from 15.6 to 18.7 μM (Table 1). Weaker inhibitory effects were instead observed with hits 1, 6, 15, 29, 30, 44, 47, and 50, obtaining IC_50_ values lower than, or comparable to, that of the reference NS3_566–585_ peptide (Table 1). To exclude that the detected inhibition of NS3-NS5 interaction by the active compounds might be due to a non-specific mechanism of colloidal formation, their inhibitory activities were also tested in the presence of Triton X-100, a well-characterized non-ionic detergent that reverses and prevents aggregates of small molecules (McGovern et al., 2003). No substantial differences in the inhibitory activity of the hits were found when tested in the presence or the absence of 0.002% Triton X-100, as the IC_50_s remained constant (data not shown).

**TABLE 1:** Biological activity of selected hit compounds.

Hit	*In vitro* activity IC_50_*^a^*, μM	Antiviral activity EC_50_*^b^*, μM	Cytotoxicity CC_50_*^c^*, μM	SI*^d^*
1	85.9 ± 14.5	>100	>250	>3
3	15.6 ± 4.5	1.9 ± 0.3	>250	>130
4	18.7 ± 2.1	>10	12.7 ± 4.0	1
6	51.4 ± 10.3	98.8 ± 10.4	>250	>3
15	123 ± 16	>100	>250	>3
29	142 ± 26	34.3 ± 9.4	35.7 ± 4.9	1
30	44.6 ± 8.1	24.6 ± 5.9	>250	>54
44	78.5 ± 16.4	97.4 ± 12.6	>100	>1
47	16.9 ± 2.5	53.5 ± 6.9	>250	>5
50	32.0 ± 6.7	13.4 ± 1.8	>250	>19
NS3_566–585_ peptide	127 ± 15	ND	ND	ND

*^a^*The IC_50_ values correspond to the compound concentrations that decreased the NS3-NS5 binding by 50% as determined by the ELISA-based NS3-NS5 interaction assay.

*^b^*The EC_50_ values represent the compound concentrations that inhibited 50% of viral plaque formation as determined by PRAs in Vero cells.

*^c^*The CC_50_ values correspond to the compound concentrations that reduced the cell viability by 50% as determined by MTT assays in Vero cells at 72 h. Reported values represent the means ± SD of data obtained from at least three independent experiments in duplicate.

*^d^*Selectivity index, i.e., the ratio between CC_50_ and EC_50_. ND, not determined.

### 3.4 Pan-serotypic antiviral activity of the selected hits against DENV

**FIGURE 4:**
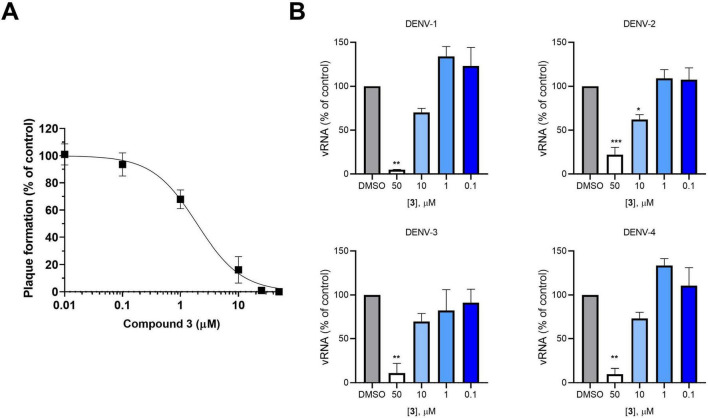
Antiviral activity of hit 3 against DENV. **(A)** Concentration-response curve for hit 3 in Vero cells infected with DENV-2. Data shown represent the means ± SD of *n* = 3 experiments performed in duplicate. **(B)** Concentration-dependent inhibition of DENV-1 to -4 viral RNA synthesis in Vero cells treated with different concentrations of hit 3 as determined by RT-qPCR at 72 h p.i. Graphs represent the mean ± SD of *n* = 3 independent experiments in duplicate. Data were analyzed by a one-way ANOVA followed by Dunnett’s multiple comparison test. (***p < 0.0001; **p < 0.005; *p < 0.05, compared to control, i.e., DMSO-treated, infected sample).

To assess their anti-DENV activity, each NS3-NS5 inhibitor active in ELISA was tested in infected Vero cells by PRAs using DENV-2 NGC strain. In parallel, all the hit compounds were also tested by MTT assays in the same cell line (i.e., Vero cells) to exclude any potential cytotoxic effect. As reported in Table 1, hits 3, 6, 30, 44, 47, and 50 were able to inhibit DENV-2 replication in PRA, without showing cytotoxicity at the tested concentrations in Vero cells. Among them, hit 3 exhibited a dose-dependent antiviral activity in the low micromolar range (EC_50_ of 1.93 μM; Figure 4A), emerging as the most potent hit with a SI > 130. On the contrary, no inhibitory effects were observed for hits 1, 4, and 15, at least at the tested concentrations (Table 1), suggesting possible cell permeability issues for these compounds. Finally, similar EC_50_ and CC_50_ values were obtained for hit 29 (Table 1), indicating that the inhibitory activity observed in the PRA was most likely due to the compound’s cytotoxicity profile.

Since the residues forming the pockets used in both SBVS approaches are conserved in all DENV serotypes, we then aimed to investigate whether the antiviral activity of the most promising hit 3 was conserved across all four DENV serotypes. To this aim, Vero cells were infected with the four different DENV serotypes and the effect of hit 3 on the synthesis of viral RNA at 72 h post-infection was assessed by RT-qPCR. As reported in Figure 4B, we observed a concentration-dependent reduction in viral RNA synthesis with all four DENV serotypes, thus pointing out a potential pan-dengue antiviral activity for hit 3.

### 3.5 Biological evaluation of structural analogues of hit 3

To explore whether structurally related compounds could display similar or more potent anti-DENV activity compared to hit 3, a series of analogues with high structural similarities with the initial hit were either synthesized or purchased, if commercially available, and subjected to biological evaluation. In particular, four compounds, i.e., 51–54, were purchased from Enamine (Supplementary Table 2), whereas 25 analogues, i.e., 55–79, were synthesized, along with hit 3, in an optimized one-step procedure, as illustrated in Supplementary Scheme 1.

To investigate the ability of the analogues to inhibit the NS3-NS5 interaction *in vitro*, dose-response analysis was carried out for each synthesized and acquired compound by our validated NS3-NS5 ELISA. The in-house synthesized hit 3 along with the NS3_86–100_ peptide were also tested at different concentrations in ELISA, as controls. As summarized in Table 2, a total of 22 analogues inhibited the NS3-NS5 binding in a dose-dependent manner, exhibiting IC_50_ values in the micromolar range. Interestingly, compounds 64, 66, 69, 71, 72, 76, and 79 exhibited IC_50_ values lower than the parental hit 3, demonstrating a similar or increased potency in disrupting the NS3-NS5 interaction.

**TABLE 2:** Inhibitory activity of the analogues of hit 3 on the NS3-NS5 binding *in vitro*.

Cpd	*In vitro* activity IC_50_*^a^*, μM	Cpd	*In vitro* activity IC_50_, μM
3	19.4 ± 7.8	66	12.5 ± 4.1
51	97.2 ± 24.3	67	>200
52	167 ± 29	68	20.1 ± 7.5
53	82.7 ± 21.5	69	1.0 ± 0.3
54	31.0 ± 18.3	70	25.3 ± 5.4
55	118 ± 23	71	11.6 ± 2.6
56	21.2 ± 3.4	72	4.6 ± 1.5
57	55.6 ± 22.7	73	>200
58	21.3 ± 7.9	74	>200
59	29.0 ± 6.9	75	40.3 ± 8.3
60	149 ± 21	76	6.3 ± 2.2
61	>200	77	>200
62	>200	78	>200
63	172 ± 31	79	13.9 ± 6.5
64	3.9 ± 1.8	NS3_86–100_ peptide	>200
65	64.9 ± 19.5	–	–

*^a^*The reported IC_50_ values represent the compound concentrations that inhibited the NS3-NS5 binding *in vitro* by 50%. Values shown are the means ± SD from *n* ≥ 3 independent experiments performed in duplicate.

Among them, compounds 64, 69, 72, and 76 exhibited IC_50_ < 10 μM and compound 69 emerged as the most potent NS3-NS5 inhibitor of the series (IC_50_ of 1.0 μM). On the contrary, compounds 61, 62, 67, 73, 74, 77, and 78 did not cause ≥ 50% reduction of NS3-NS5 interaction at the tested concentrations (Table 2).

The three most promising analogues (i.e., compounds 64, 69, and 72) that inhibited the NS3-NS5 interaction *in vitro* with IC_50_ values ≤ 5 μM were further tested to assess their anti-DENV activities in infected Vero cells by PRA, as well as their cytotoxic effects in the same cell line by MTT assays. In-house synthesized hit 3 was included in both experiments as a control. As shown in Table 3, treatment with compounds 64, 69, and 72 showed a concentration-dependent anti-DENV activity in PRA against the NGC strain. Compounds 64 and 72, exhibiting EC_50_ of 2.1 and 2.8 μM, respectively, showed comparable antiviral potency with hit 3 (EC_50_ of 1.8), while a higher EC_50_ was obtained with 69 (EC_50_ of 12.4 μM, Table 3). However, the SI of all three analogues (SIs up to 75) was found to be lower than that of the parental hit, as they showed higher cytotoxicity than compound 3 (Table 3).

**TABLE 3:** Anti-DENV activity and cytotoxicity of the best analogues of hit 3 in Vero cells.

Cpd	Antiviral activity EC_50_*^a^*, μM	Cytotoxicity CC_50_*^b^*, μM	SI*^c^*
3	1.8 ± 0.6	>250	>130
64	2.1 ± 1.6	39.6 ± 10.2	19
69	12.4 ± 5.8	72.0 ± 17.2	6
72	2.8 ± 1.9	211 ± 20	75

*^a^*The EC_50_ values represent the compound concentrations that inhibited 50% of viral plaque formation as determined by PRAs in Vero cells.

*^b^*The CC_50_ values correspond to the compound concentrations that reduced the cell viability by 50% as determined by MTT assays in Vero cells at 72 h.

*^c^*Selectivity index, i.e., the ratio between CC_50_ and EC_50_. Reported values represent the means ± SD of data obtained from *n* ≥ 3 independent experiments in duplicate.

### 3.6 Biophysical characterization of the binding of NS3 and NS5 by microscale thermophoresis

**FIGURE 5:**
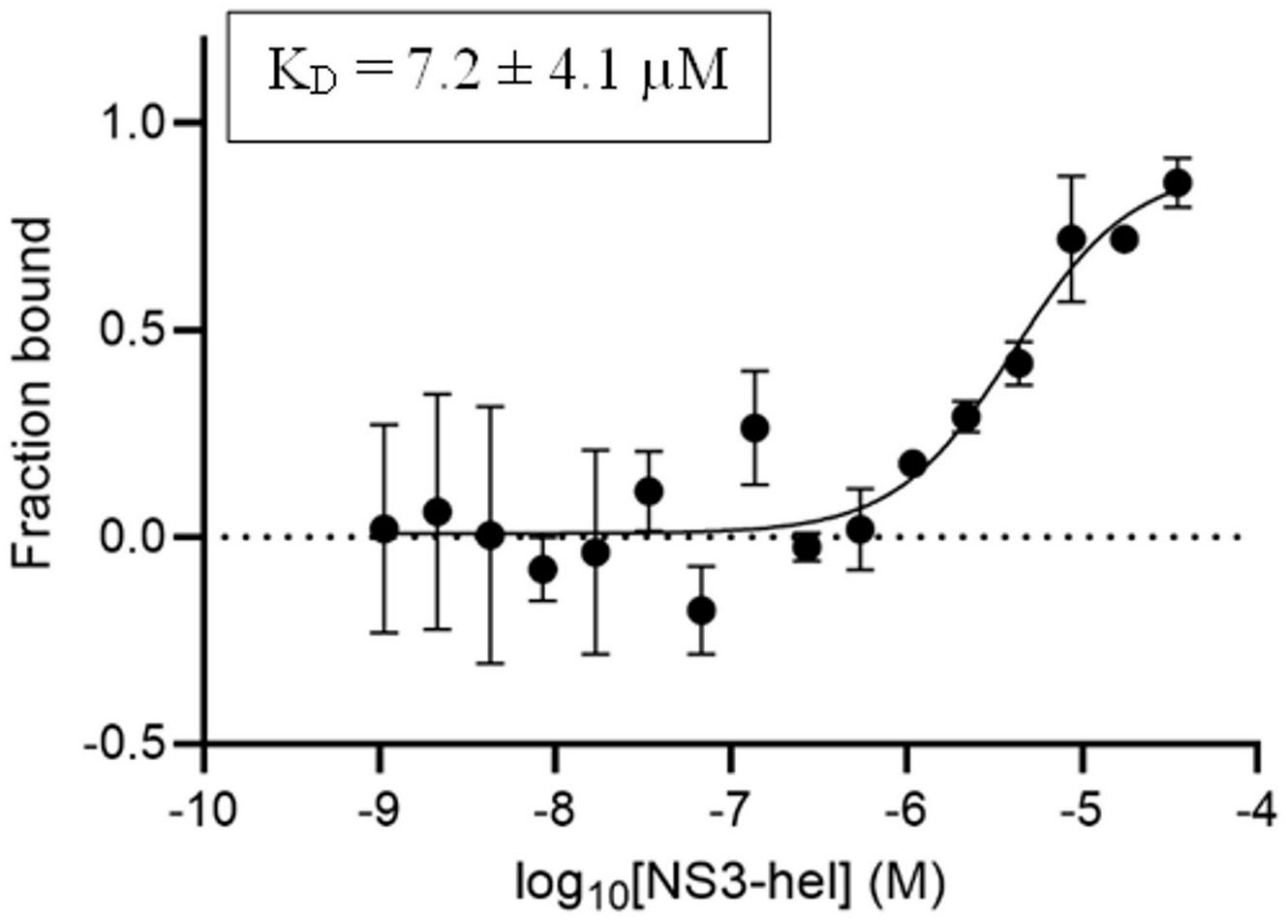
Biophysical characterization of NS3-NS5 interaction by microscale thermophoresis (MST). Concentration-dependent binding curve of NS3-hel to labeled NS5-RdRp. MST measurements were performed at 670 nm, medium MST power, and 20% LED power. The hot region in the MST curves used to determine the K_D_ values was 2.5 s. Data are represented as fraction bound vs. ligand concentration (M). Graph shows the mean ± SD obtained from *n* = 3 independent experiments.

Before assessing the binding affinity of the compound with the highest SI (i.e., compound 3) to either protein, we first characterized the interaction between NS3-hel and NS5-RdRp by microscale thermophoresis (MST), a biophysical technique that allows to quantitatively study the interaction between macromolecules in solution and without requiring surface immobilization (Seidel et al., 2013). To optimize the experimental conditions for the binding experiments (i.e., buffer composition, the laser power, etc.) NS3-NS5 binding was first confirmed in the MST buffer at a single NS3-hel concentration of 35 μM (Supplementary Figure 2). Then, serial dilutions of NS3-hel were used to determine the K_D_ for the interaction with NS5-RdRp and a value of 7.2 ± 4.1 μM was determined (Figure 5). This is consistent with the K_D_ of 4.4 ± 0.3 μM previously reported for NS5RdRp-NS3hel interaction determined by SPR (Zou et al., 2011). We also detected the binding of NS5-RdRp to labeled NS3-hel under the same experimental conditions (data not shown).

### 3.7 Hit compound 3 binds to NS5

**FIGURE 6:**
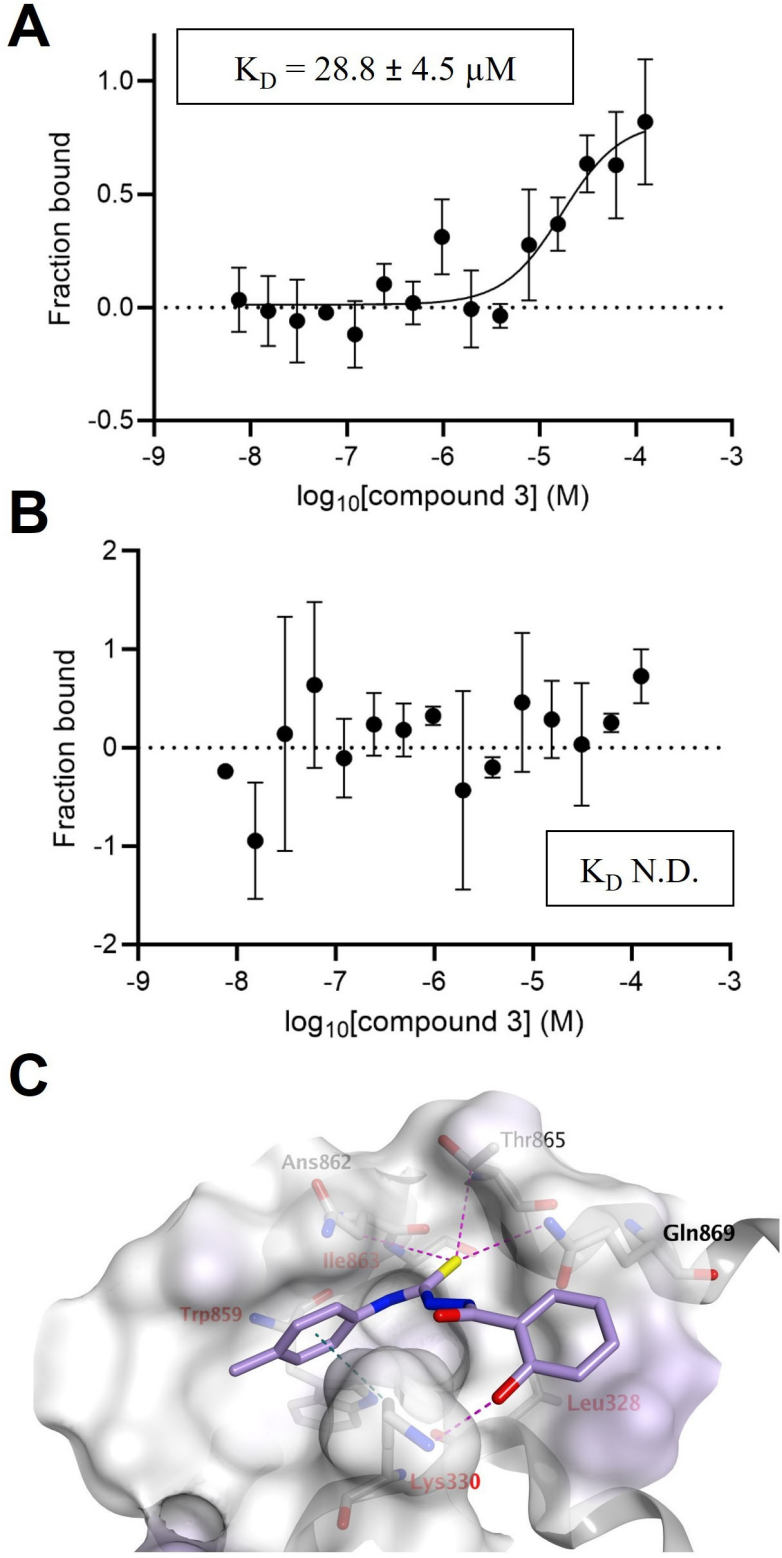
Hit 3 binds to NS5-RdRp. **(A)** Concentration-dependent binding curve of hit 3 to labeled NS5-RdRp. Microscale thermophoresis (MST) measurements were performed at 670 nm, medium MST power, and 40% LED power. The hot region in the MST curves used to determine the K_D_ values was 10 s. Data are represented as fraction bound *vs* ligand concentration (M). Graph shows the means ± SD obtained from *n* = 3 independent experiments. **(B)** Concentration-dependent experimental data of hit 3 binding to labeled NS3-Hel. MST measurements were performed at 670 nm, medium MST power, and 60% LED power. The hot region in the MST analysis was 1.5 s. Data are represented as fraction bound vs. ligand concentration (M). Graph shows the means ± SD obtained from *n* = 3 independent experiments. **(C)** Docking pose of hit 3 on DENV-2 NS5 cavity B. The best binding pose of hit 3 within cavity B (score: –5.8 Kcal/mol), as used in the NS5 SBVS, is shown, highlighting the key residues that define the pocket shape and the interactions that stabilize the compound.

To determine the target of hit 3, we investigated by MST its binding to either NS3-Hel or NS5-RdRp, since this compound was selected as a potential dissociative inhibitor in both virtual screenings targeting NS3 and NS5. First, the binding of hit 3 to NS5-RdRp was investigated at a single concentration and we detected a potential interaction of hit 3 to NS5-RdRp (Supplementary Figure 3A). Successively, serial dilutions of hit 3 were used to obtain a concentration-dependent binding curve, determining a K_D_ of 28.8 ± 4.5 μM (Figure 6A). On the other hand, we were not able to detect the binding of hit 3 to NS3-hel at the same test concentrations (Figure 6B). As a control for the specificity of the binding of hit 3 to NS5, MST experiments were repeated with an unrelated protein, i.e., SARS-CoV-2 M^pro^. No binding was detected up to 125 μM compound concentration (Supplementary Figure 3B). Collectively, these results demonstrated that hit 3 specifically interacts with NS5-RdRp. This finding is further supported by the predicted docking pose of hit 3 within NS5 cavity B, as shown in Figure 6C. The molecule was well accommodated within the pocket, fully occupying the available space and stabilized by multiple interactions with the surrounding residues. The sulfur atom, acting as a hydrogen bond acceptor, formed hydrogen bonds with the side chains of Thr858, Asn862, and Thr865. Interestingly, an additional hydrogen bond was also predicted between the phenolic oxygen of hit 3, which is negatively charged at physiological pH, and the side chain of the positively charged Lys330. In addition, Lys330 was also involved in a CH–π interaction between its side-chain methylene group and the electron-rich π-system of the *p*-toluidine ring of hit 3. As outlined in the introduction, Lys330 is highly conserved across DENV serotypes and is essential for maintaining the interaction with NS3 (Zou et al., 2011). Overall, these findings are in agreement with the observation that hit 3 interferes with the NS3-NS5 interaction by binding to NS5.

## 4 Discussion

Interactions between all the seven viral nonstructural proteins of DENV are crucial events to establish a membrane-bound replication complex (RC), which is essential for the viral life cycle inside host cells. A functional RC assembly is responsible for the viral RNA replication, and by engaging with a number of host proteins, it facilitates the packaging of new virions and hijacks innate immune responses [reviewed in Van Den Elsen et al. (2021)]. Disruption of such PPIs within the RC network has been recently proven a successful strategy by the development of potent non-enzymatic anti-DENV agents targeting either NS2B-NS3 (Phong et al., 2011; Pambudi et al., 2013) or NS3-NS4B interactions (Kaptein et al., 2021; Moquin et al., 2021; Goethals et al., 2023; Wang et al., 2025). These include two NS3-NS4B inhibitors, mosnodenvir (previously JNJ-1802) and NITD-688, that have progressed into stage II of clinical trials for treating DENV infections in patients. Within the RC, NS3 and NS5 form its functional core, and disruption of their interaction leads to the impairment of the viral replication, as shown by mutagenesis studies and the identification of dissociative inhibitors through various structure-based approaches or phenotypic antiviral screenings (Tay et al., 2015; Celegato et al., 2023; Vincetti et al., 2015; Cannalire et al., 2020; Yang et al., 2022). Analysis of the interacting regions of both NS5 and NS3 proteins revealed two potential hotspot sites; i.e., cavity B in NS5 and site A in NS3 (Malet et al., 2008; Munawar et al., 2018), paving the way to dual and complementary structure-based drug design opportunities.

In this study, we described a new SBVS approach for the identification of new small molecules that inhibit the NS3-NS5 interaction through two parallel SBVS strategies on hot pockets involved in the interaction between NS5 RdRp (cavity B) and NS3 hel (site A). The identified new small molecules display chemical scaffolds that are substantially different from the NS3-NS5 inhibitors reported previously. They effectively disrupted the *in vitro* NS3-NS5 binding in a dose-dependent manner as well as inhibited DENV replication in cell-based assays at non-cytotoxic concentrations. Among the active NS3-NS5 inhibitors, hit 3 emerged as the most potent hit, as it inhibited DENV-2 replication in PRA in a low micromolar concentration range (EC_50_ of 1.8–1.9 μM) and exhibited an SI > 130 (Table 1). Hit 3 also affected the replication of all four DENV serotypes in Vero cells (Figure 4B), thus exhibiting a pan-serotype anti-DENV potential. To the best of our knowledge, no prior studies have reported antiviral activity of this compound against any virus. Regarding hit 3 analogs, compound 69 has been included in a Korean patent application for its activity against MERS coronavirus by targeting viral entry (patent KR20200068815A, Jeong et al., 2020).

Ligand-binding analysis using our validated MTS assay demonstrated that hit 3 directly binds to the NS5 RdRp domain, exhibiting a K_D_ of 28.8 μM (Figure 6A). This binding affinity is consistent with its ability to block the NS3-NS5 interaction in ELISA, with IC_50_ values ranging from 15.6 ± 4.5 to 19.4 ± 7.8 μM (Tables 1, 3, respectively). Interestingly, although hit 3 was initially selected as a top-ranked hit in both NS3 and NS5 SBVS, no binding was observed to the NS3 hel in the MST assay (Figure 6B). These findings suggested that the antiviral activity of hit 3 might be mediated through interaction with the NS5 RdRp at the interface with NS3. Consistent with these results, molecular docking simulations indicated that hit 3 fits well within the cavity B in the NS5 RdRp domain, forming favorable interactions with key pocket residues, including the conserved Lys330 (Figure 6C), which is critical for NS3 binding. Indeed, previous studies showed that alanine substitution of Lys330 disrupts the NS3-NS5 interaction (Zou et al., 2011). The proposed binding mode for hit 3 aligns with those of other structurally distinct NS3-NS5 inhibitors reported in the literature, which also target cavity B of NS5 (Celegato et al., 2023; Vincetti et al., 2015; Cannalire et al., 2020), further reinforcing its relevance as a primary druggable pocket for the development of effective anti-DENV agents acting by a novel dissociative mechanism. In contrast, although site A within the NS3 helicase domain has been demonstrated to play a crucial role in mediating NS3-NS5 interaction, its potential as a druggable target remains unconfirmed, as, to the best of our knowledge, no anti-DENV inhibitors have yet been reported to bind this site.

To confirm the specificity of the NS3-NS5 inhibition around the chemical scaffold of hit 3, we synthesized or purchased and then tested a series of 29 structural analogues in our validated NS3-NS5 interaction ELISA. Among them, 22 analogues retained the ability to disrupt the interaction between NS3 and NS5 *in vitro* in a dose-dependent manner and 7 compounds (i.e., 64, 66, 69, 71, 72, 76, and 79) showed improved or similar NS3-NS5 inhibitory activity compared to the parental compound, resulting in a decrease in the IC_50_ values. In particular, compound 69 was found the most potent NS3-NS5 inhibitor *in vitro*, exhibiting an IC_50_ 15-fold lower than that of hit 3 (IC_50_ of 1.0 μM versus 15.6 μM, respectively). Although it is not yet possible to generate a comprehensive SAR study, some preliminary observations can be drawn. In particular, the thiourea group seems very important for imparting NS3-NS5 disruption activity (see the difference between compound 3 and 78), and pyridine (compounds 64 and 69) or bulky substituents (compound 72) at the *para* position on the benzoyl group were well-tolerated. In addition, the insertion of bulky (compound 76) or strong electron-donating substituents (i.e., NMe_2_, compounds 66 and 69) at the *p*-position of the *N*-phenyl ring led to an increase of NS3-NS5 interaction inhibitory activity. The best analogues (i.e., compounds 64, 69, and 72) were confirmed to possess anti-DENV activity in infected cells, showing low-micromolar antiviral potency, which was comparable with that of hit 3. However, these analogues displayed some cytotoxic effects at higher concentrations, although still retaining good SIs. Overall, although we have not significantly improved the hit activity in cells, the interesting results obtained for the analogues have confirmed that the scaffold we have identified confers NS3-NS5 inhibitory activity and can be used as a starting point for further structural optimization studies. Future efforts will be focused on designing more analogues to identify new NS3-NS5 inhibitors with improved anti-DENV activity and toxicity profiles.

Crucial interactions between viral nonstructural proteins, including the NS3-NS5 binding, can represent very attractive drug targets (Loregian et al., 2002; Loregian and Palù, 2013). Importantly, the interacting sequences on both NS3 and NS5 (i.e., residues 566–585 of NS3 and 320–341 of NS5, using the DENV-2 numbering) are well conserved across the four DENV serotypes (Tay et al., 2015). Targeting structures with a high conservation degree usually correlates with the potential to develop broad-spectrum inhibitors and this corroborates with the pan-serotype anti-DENV activity exhibited by hit 3. Therefore, the development of NS3-NS5 interaction inhibitors based on the chemical scaffold we have identified could pave the way to the discovery of antiviral drugs with broad-spectrum activity against different DENV strains and serotypes.

## 5 Conclusion

To conclude, this work presents a dual structure-based screening strategy conducted on two pockets of DENV NS3 and NS5 that successfully identified a series of new small molecules targeting the essential NS3-NS5 interaction in dengue virus. The most promising inhibitor, hit 3, binds directly to NS5 and disrupts its interaction with NS3, resulting in low-micromolar, pan-serotype anti-DENV activity in cell-based assays. Several structural analogues of hit 3 retained both anti-DENV activity and the ability to disrupt the NS3-NS5 interaction *in vitro*. These findings confirm the identification of a novel scaffold endowed with broad antiviral activity against DENV through the inhibition of the NS3-NS5 interaction. This work lays the foundation for the further development and optimization of this class of small molecules as promising antiviral candidates for the treatment of DENV infections.

## Data Availability

The original contributions presented in this study are included in this article/Supplementary material, further inquiries can be directed to the corresponding authors.

## Footnotes

1
https://saves.mbi.ucla.edu/

2
http://www.specs.net

3
https://www.chemdiv.com

4
https://enamine.net/

5
https://lifechemicals.com

6
http://zinc15.docking.org/patterns/home
