# Supplementary material for: New dengue virus inhibitors targeting NS3-NS5 interaction identified by in silico screening
Source: Front Microbiol. 2025 Oct 31;16:1663404. doi: 10.3389/fmicb.2025.1663404 (PMC12615485; doi:10.3389/fmicb.2025.1663404)
Supplement: Supplementary file 1 [file Data_Sheet_1.pdf]

Supplementary Material for

New dengue virus inhibitors targeting NS3-NS5 interaction identified by *in silico* screening

Giulio Nannetti [1,2,*], Beatrice Mercorelli [3], Alessandro Bazzacco [3], Nicolò Santi [4,†], Marta Celegato [3], Salvatore Ferla [1], Mattia Sturlese [4], Niklaas J. Buurma [5], Andrea Brancale [2,6,‡], Arianna Loregian [3,7,*,‡]

Table of contents

Figures and Scheme

Figure S1. Inhibitory activity of the hits on the DENV NS3–NS5 interaction.

Figure S2. Single-concentration binding check of NS5 to NS3-hel.

Figure S3. Single-concentration binding check of compound **3** to NS5-RdRp and SARS-CoV-2 M^{pro}.

Scheme S1. General synthetic procedure for the preparation of hit **3** analogues (compounds **55-79**).

Tables

Table S1. Hit candidates selected by the virtual screenings.

Table S2. Structural analogues of hit **3** purchased from Enamine.

Supplementary methods

1. Chemistry
2. Synthesis and characterization of hit **3** and analogues **55-79**
3. ^{1}H-NMR, ^{13}C- and UPLC spectra for compounds **3**, **55-79**
4. References

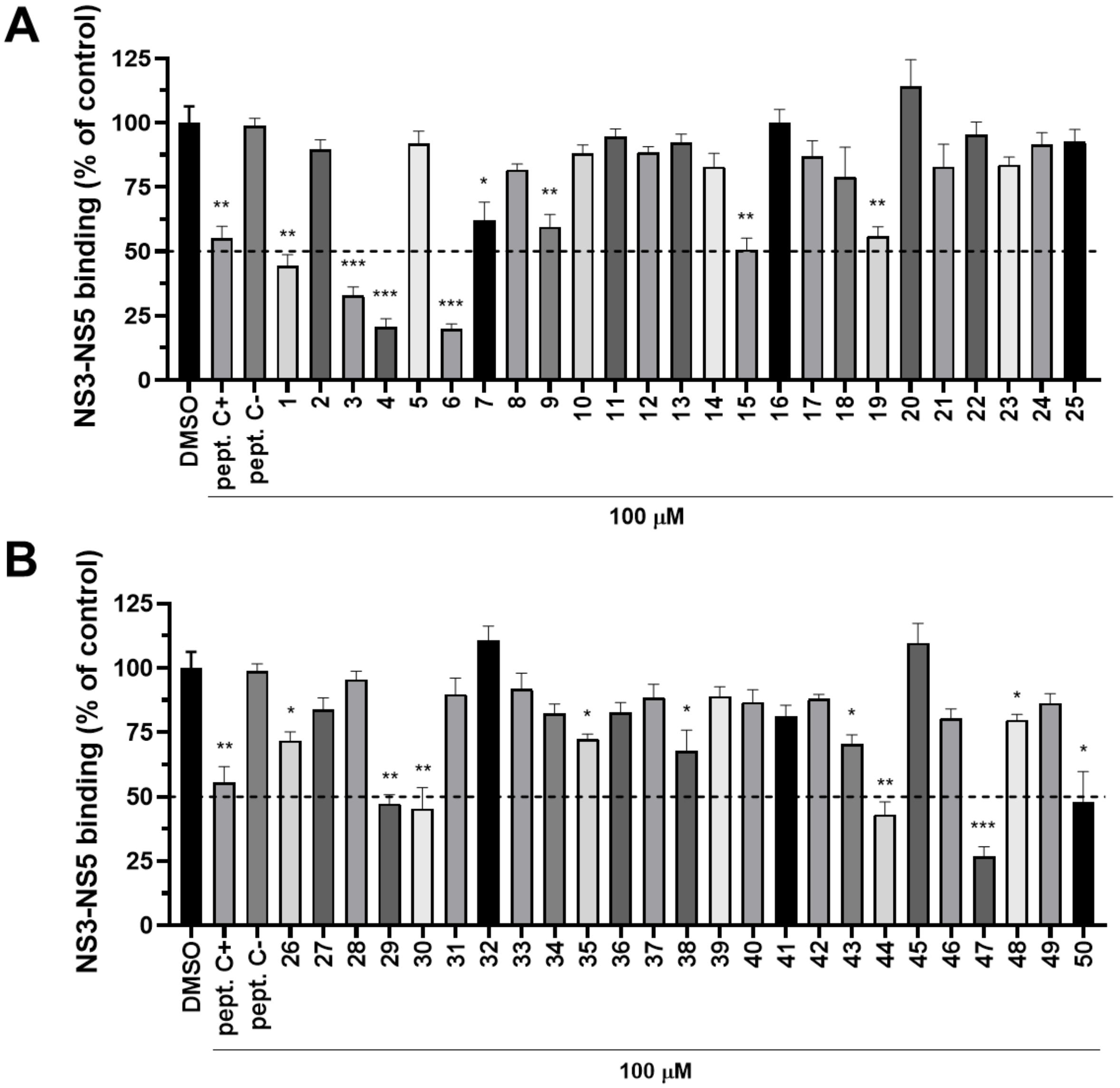

Figure S1. Inhibitory activity of the hits on the DENV NS3-NS5 interaction. The effect of hits **1 - 25** (A) and hits **26 – 50** (B) was evaluated in the NS3-NS5 interaction assay at 100 µM. The $NS3_{566-585}$ (pept. C+) and $NS3_{566-585}$ peptides (pept. C-) were also tested at 100 µM in each assay as positive and negative controls, respectively. All data shown represent the means ± SD of data derived from n ≥ 3 independent experiments in duplicate. Data were analysed by an unpaired two-tailed Student's t-test. (**p* < 0.05, ***p* < 0.01, ****p* < 0.001 of compound-related samples *versus* the mock control treated with DMSO).

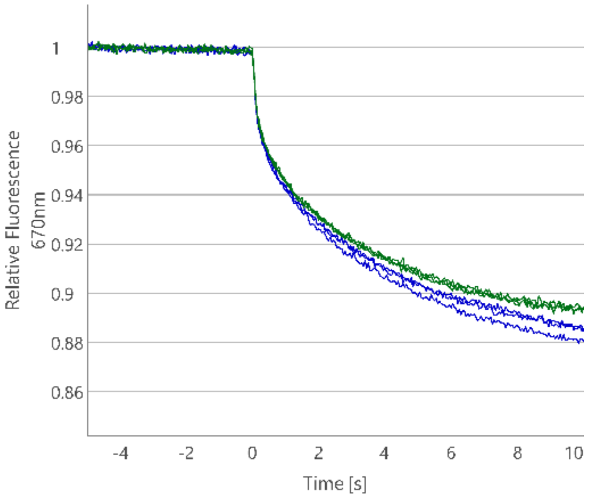

Figure S2. **Single-concentration binding check of NS5 to NS3-hel.** MST fluorescence traces comparing NS5-RdRp alone (blue traces) and NS5-RdRp with NS3-hel (35 µM) (green traces). The curves correspond to a representative experiment, with each condition tested in triplicate. MST measurements were performed at 670 nm with medium MST power, using 20% LED power.

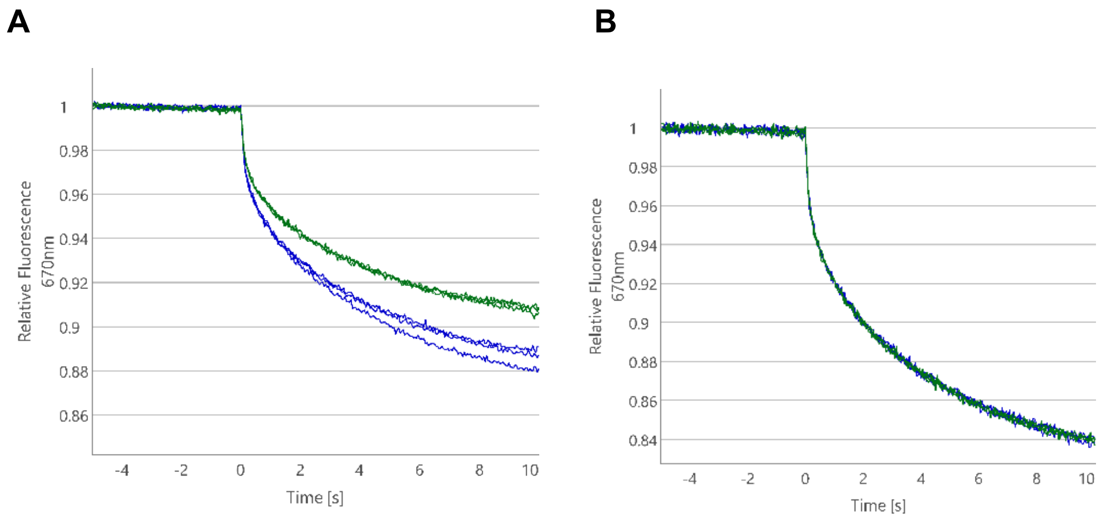

Figure S3. Single-concentration binding check of compound **3** to NS5-RdRp and SARS-CoV-2 M^{pro}. MST fluorescence traces comparing NS5-RdRp (A) and M^{pro} (B) alone (blue traces) and in the presence of compound **3** at 250 µM and 125 µM concentration, respectively (green traces). The curves correspond to a representative experiment, with each condition tested in triplicate. MST measurements were performed at 670 nm with medium MST power, using 40% LED power for NS5-RdRp and 60% LED power for M^{pro}, respectively.

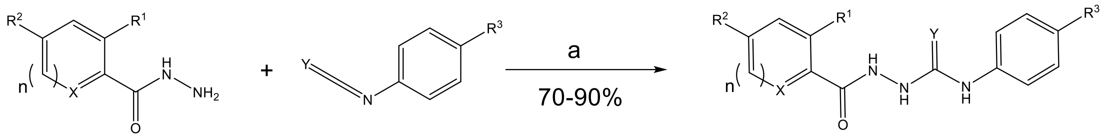

3 X=C, Y=S, n=1, R^1=OH, R^2=H, R^3=Me
55 X=C, Y=S, n=1, R^1=H, R^2=CF_3, R^3=Me
56 X=C, Y=S, n=1, R^1=OMe, R^2=H, R^3=Me
57 X=C, Y=S, n=1, R^1,R^2=H, R^3=Me
58 X=C, Y=S, n=1, R^1=F, R^2=H, R^3=Me
59 X=C, Y=S, n=1, R^1=Me, R^2=H, R^3=Me
60 X=C, Y=S, n=1, R^1=H, R^2=Me, R^3=Me

61

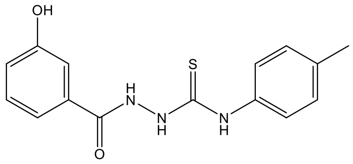

62 X=C, Y=S, n=1, R^1=H, R^2=OH, R^3=Me
63 X=C, Y=S, n=1, R^1,R^2=OH, R^3=Me
64 X=N, Y=S, n=1, R^1,R^2=H, R^3=Me
65 X=S, Y=S, n=0, R^1,R^2=H, R^3=Me
66 X=C, Y=S, n=1, R^1=OH, R^2=H, R^3=$N(Me)_2$
67 X=C, Y=S, n=1, R^1=OH, R^2=H, R^3=NO_2
68 X=O, Y=S, n=0, R^1,R^2=H, R^3=Me
69 X=N, Y=S, n=1, R^1,R^2=H, R^3=$N(Me)_2$
70 X=S, Y=S, n=0, R^1,R^2=H, R^3=$N(Me)_2$
71 X=O, Y=S, n=0, R^1,R^2=H, R^3=$N(Me)_2$
72 X=C, Y=S, n=1, R^1=H, R^2=*t-bu*, R^3=Me
73 X=C, Y=S, n=1, R^1,R^2=CF_3, R^3=Me
74 X=C, Y=S, n=1, R^1=OH, R^2=H, R^3=OMe
75 X=C, Y=S, n=1, R^1=OH, R^2=H, R^3=CN
76 X=C, Y=S, n=1, R^1=OH, R^2=H, R^3=*t-bu*
77 X=C, Y=S, n=1, R^1=H, R^2=CN, R^3=Me

78

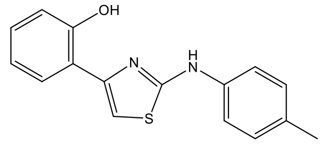

79 X=C, Y=O, n=1, R^1=OH, R^2=H, R^3=Me

Scheme S1. General synthetic procedure for the preparation of hit **3** analogues (compounds **55**-**79**); a. absolute ethanol, reflux, 5h.

Table S1. Hit candidates selected by the virtual screenings.

Compounds	Primary code / Compound ID	Vendor	SBVS / Selection
**1**	Z59655986	Enamine	Both NS3 and NS5
**2**	Z910854744	Enamine	Both NS3 and NS5
**3**	Z45833251	Enamine	Both NS3 and NS5
**4**	Z1423688307	Enamine	NS3
**5**	Z364310504	Enamine	NS3
**6**	Z1000172522	Enamine	NS3
**7**	Z212692824	Enamine	NS3
**8**	Z2040073728	Enamine	NS3
**9**	Z1516251893	Enamine	NS3
**10**	Z285040928	Enamine	NS3
**11**	Z359420058	Enamine	NS3
**12**	Z1171546223	Enamine	NS3
**13**	Z45913263	Enamine	NS3
**14**	AI-204/31695001	Specs	NS3
**15**	AO-476/40672242	Specs	NS3
**16**	Z1103041634	Enamine	NS5
**17**	Z594417796	Enamine	NS5
**18**	Z1369849002	Enamine	NS5
**19**	Z1512512272	Enamine	NS5
**20**	Z1015110076	Enamine	NS5
**21**	Z973549454	Enamine	NS5
**22**	Z1182757190	Enamine	NS5
**23**	Z973149686	Enamine	NS5
**24**	Z15477346	Enamine	NS5
**25**	Z220150438	Enamine	NS5
**26**	Z978968884	Enamine	NS5
**27**	Z105287584	Enamine	NS5
**28**	Z645237800	Enamine	NS5

**29**	Z237696050	Enamine	NS5
**30**	Z1677743628	Enamine	NS5
**31**	Z212164116	Enamine	NS5
**32**	Z3032605004	Enamine	NS5
**33**	Z372502806	Enamine	NS5
**34**	AN-329/40254080	Specs	NS5
**35**	AP-853/43261339	Specs	NS5
**36**	Y020-2789	ChemDiv	NS5
**37**	C328-0331	ChemDiv	NS5
**38**	Z1521893938	Enamine	NS3
**39**	Z31996616	Enamine	NS3
**40**	Z1536616273	Enamine	NS3
**41**	Z138425192	Enamine	NS3
**42**	Z1165953787	Enamine	NS3
**43**	Z1725346253	Enamine	NS3
**44**	Z1420687767	Enamine	NS5
**45**	Z165982390	Enamine	NS3
**46**	AG-690/12071213	Specs	NS3
**47**	AF-399/13426042	Specs	NS5
**48**	AK-968/40605527	Specs	NS3
**49**	8006-2677	ChemDiv	NS3
**50**	8017-2714	ChemDiv	NS3

Table S2. Hit **3** and structural analogues purchased from Enamine.

Compounds	Structure	Vendor primary code
**Hit 3**	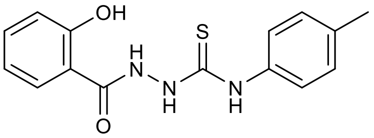	Z45833251
**51**	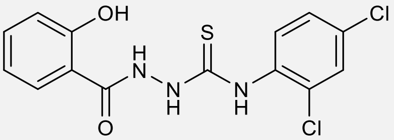	Z45833253
**52**	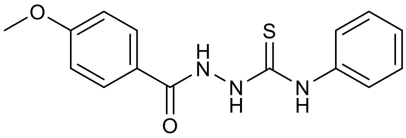	Z33098708
**53**	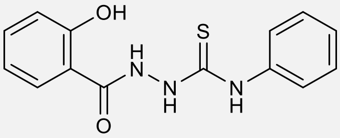	Z45833242
**54**	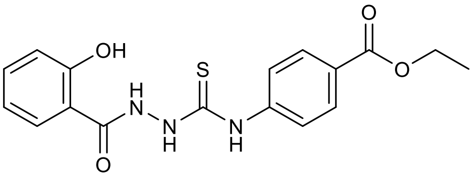	Z45833263

Supplementary methods - Synthesis and characterization of hit 3 and analogues 55-79.

1. General information

1.1 Chemistry

All solvents and reagents were used as obtained from commercial sources, unless otherwise indicated. All solvents used for chromatography were HPLC grade from Fisher Scientific (Loughborough, UK). All reactions were performed under a nitrogen atmosphere. ^{1}H and ^{13}C NMR spectra were recorded with a Bruker Avance III HD spectrometer operating at 500 MHz for ^{1}H and 125 MHz for ^{13}C, with Me_4Si as an internal standard (Bruker, Coventry, UK). Dimethyl sulfoxide (DMSO) was used as the solvent for NMR experiments, unless otherwise stated. ^{1}H chemical shifts values (δ) are referenced to the residual nondeuterated components of the NMR solvents (δ_{DMSO} = 2.50). The ^{13}C chemical shifts (δ) are referenced to DMSO (central peak, δ= 39.8 ppm). TLC was performed on silica gel 60 F254 plastic sheets. Flash column chromatography was performed using an Interchim automated system (Interchim, Montluçon, France). UPLC-MS analysis was conducted on a Waters UPLC system with both Diode Array detection and Electrospray (+0ve and −0ve ion) MS detection (Waters, Wilmslow, UK). The stationary phase was a Waters Acquity UPLC BEH C18 1.7 µm, 2.1 × 50 mm column. The mobile phase was LC-MS grade H_2O containing 0.1% formic acid (A) and LC-MS grade MeCN containing 0.1% formic acid (B). Column temperature: 40 ºC. Sample diluent: MeCN. Sample concentration 1 µg/mL. Injection volume 2 µL. Three alternative methods were used: Linear gradient standard method (A): 90% A (0.1 min), 90–0% A (2.5 min), 0% A (0.3 min), 90% A (0.1 min); flow rate 0.5 mL/min. Linear gradient standard method (B): 90% A (0.1 min), 90–0% A (2.1 min), 0% A (0.8 min), 90% A (0.1 min); flow rate 0.5 mL/min. Linear gradient standard method (C): 90% A (0.1 min), 90–0% A (1.5 min), 0% A (1.4 min), 90% A (0.1 min); flow rate 0.5 mL/min. All compounds tested in biological assays were >95% pure. Compound **51-54** were purchased from Enamine (Kyiv, Ukraine). Details for the preparation and full characterization of compounds **3**, **55-79** are given below.

2. Synthesis, procedures, and analytical data

2.1. Synthesis and characterization of compound 3

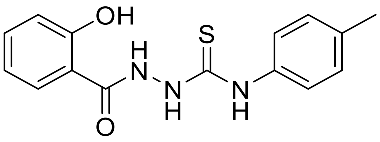

The synthesis of the hit compound **3** was carried out from a known procedure (Cansiz et al., 2009).

General procedure for the synthesis of 2-(2-hydroxybenzoyl)-*N*-(p-tolyl)hydrazine-1-carbothioamide (**3**): 0.76 g (5.0 mmol, 1 eq.) of salicylic acid hydrazide were dissolved in 50 mL of absolute ethanol and heated at 50 °C until completely dissolved. *p*-tolyl isothiocyanate (0.75 g, 5.0 mmol, 1 eq.) was added and the reaction mixture was refluxed for 5 h. After the completion of the reaction, the crude product was collected via filtration, and washed several times with Et_2O to afford **3** (1.06 g, 71% yield).

^{1}H-NMR (500 MHz, DMSO), δ: 11.92 (s, 1H), 10.73 (br s, 1H), 9.83 (s, 2H), 7.91 (d, *J*= 6.3 Hz, 1H), 7.48 (dt, *J*= 6.3 Hz, 1H), 7.35 (d, *J*= 7.5 Hz, 2H), 7.18 (t, *J*=7.2 Hz, 2H), 6.97 (dd, *J*= 15.8 and 7.8 Hz, 2H), 2.31 (s, 3H) ppm.

^{13}C-NMR (126 MHz, DMSO), δ: 136.5, 134.6, 128.8, 119.0, 115.6, 115.1, 21.0 ppm.

MS (ESI-(+)): m/z: calculated for $C_{15}H_{15}N_3O_2S$: 301.09, $[M+H]^+$; found: 302.09.

2.2. Synthesis and characterization of compounds 55-79

The synthesis of compounds **55-79** was carried out using the same procedure applied for compound **3** in the section 2.1, unless otherwise specified.

2.2.1. Characterization data for compound 55

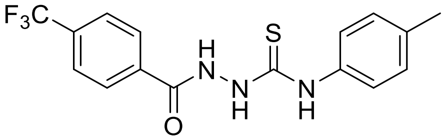

^{1}H-NMR (500 MHz, DMSO), δ: 10.76 (s, 1H), 9.75 (d, *J*= 32.1 Hz, 2H), 8.14 (d, *J*= 8.1 Hz, 2H), 7.90 (d, *J*= 8.3 Hz, 2H), 7.30 (s, 2H), 7.14 (d, *J*= 8.2 Hz, 2H), 2.29 (s, 3H) ppm.

^{13}C-NMR (126 MHz, DMSO), δ: 136.8, 132.2, 130.6, 129.3, 126.3, 125.5, 125.0, 20.9 ppm.

MS (ESI-(+)): m/z: calculated for $C_{16}H_{14}F_3N_3OS$: 353.08, $[M+H]^+$; found: 354.08.

2.2.2. Characterization data for compound 56

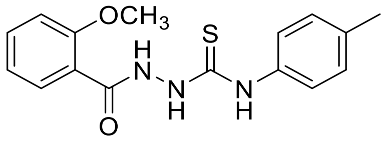

^{1}H-NMR (500 MHz, DMSO), δ: 9.77 (m, 2H), 7.88 (s, 1H), 7.55 (m, 2H), 7.40 (m, 1H), 7.20 (d, *J*= 8.3 Hz, 1H), 7.14 (d, *J*= 8.2 Hz, 2H), 7.04 (t, *J*= 7.4 Hz, 1H), 3.90 (s, 3H), 2.29 (s, 3H) ppm.

^{13}C-NMR (126 MHz, DMSO), δ: 157.7, 136.9, 133.5, 130.5, 121.0, 112.5, 56.2, 21.1 ppm.

MS (ESI-(+)): m/z: calculated for $C_{16}H_{17}N_3O_2S$: 315.10, $[M+H]^+$; found: 316.10.

2.2.3. Characterization data for compound 57

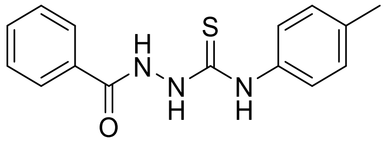

^{1}H-NMR (500 MHz, DMSO), δ: 10.52 (s, 1H), 9.73 (s, 1H), 9.63 (s, 1H), 7.95 (d, *J*= 7.9 Hz, 2H), 7.58 (t, *J*= 7.4 Hz, 1H) 7.50 (t, *J*= 7.5 Hz, 2H), 7.30 (s, 2H), 7.13 (d, *J*= 8.1 Hz, 2H), 2.28 (s, 3H) ppm.

^{13}C-NMR (126 MHz, DMSO), δ: 137.2, 133.1, 131.4, 127.1, 56.6, 21.2 ppm.

MS (ESI-(+)): m/z: calculated for $C_{15}H_{15}N_3OS$: 285.09, $[M+H]^+$; found: 286.09.

2.2.4. Characterization data for compound 58

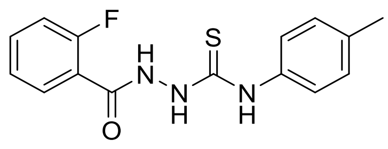

^{1}H-NMR (500 MHz, DMSO), δ: 10.31 (s, 1H), 9.74 (s, 1H), 9.65 (s, 1H), 7.85 (s, 1H), 7.60 (m, 1H), 7.33 (m, 4H), 7.14 (d, *J*= 8.2 Hz, 2H), 2.29 (s, 3H) ppm.

^{13}C-NMR (126 MHz, DMSO), δ: 137.2, 134.0, 130.2, 125.2, 116.2, 20.7 ppm.

MS (ESI-(+)): m/z: calculated for $C_{15}H_{14}FN_3OS$: 303.08, $[M+H]^+$; found: 304.08.

2.2.5. Characterization data for compound 59

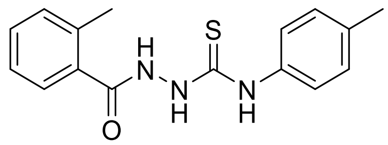

^{1}H-NMR (500 MHz, DMSO), δ: 10.18 (s, 1H), 9.66 (s, 2H), 7.65 (s, 1H), 7.38 (tt, *J* = 4.5 and 2.2 Hz, 1H), 7.34 (br s, 2H), 7.26 (m, 2H), 7.15 (d, *J*= 8.1 Hz, 2H), 2.41 (s, 3H), 2.29 (s, 3H) ppm.

^{13}C-NMR (126 MHz, DMSO), δ: 136.7, 130.6, 130.2, 129.8, 127.9, 125.7, 125.3, 20.4, 19.3 ppm.

MS (ESI-(+)): m/z: calculated for $C_{16}H_{17}N_3OS$: 299.11, $[M+H]_+$; found: 300.10.

2.2.6. Characterization data for compound 60

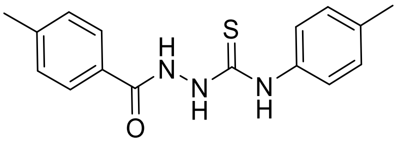

^{1}H-NMR (500 MHz, DMSO), δ: 10.43 (s, 1H), 9.71 (s, 1H), 9.61 (s, 1H), 7.86 (d, *J*= 8.1 Hz, 2H), 7.31 (d, *J*= 8.2 Hz, 4H), 7.13 (d, *J*= 8.2 Hz, 2H), 2.38 (s, 3H), 2.29 (s, 3H) ppm.

^{13}C-NMR (126 MHz, DMSO), δ: 142.3, 137.1, 130.2, 129.2, 128.8, 128.2, 21.5, 21.0 ppm.

MS (ESI-(+)): m/z: calculated for $C_{16}H_{17}N_3OS$: 299.11, $[M+H]^+$; found: 300.10.

2.2.7. Characterization data for compound 61

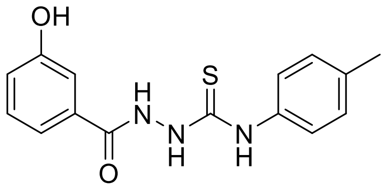

^{1}H-NMR (500 MHz, DMSO), δ: 10.39 (s, 1H), 9.72 (s, 2H), 9.59 (s, 1H), 7.40-7.25 (m, 5H), 7.13 (d, *J*= 8.2 Hz, 2H), 6.96 (ddd, *J*= 8.1 2.5 and 0.9 Hz, 1H), 2.28 (s, 3H) ppm.

^{13}C-NMR (126 MHz, DMSO), δ: 157.5, 137.1, 134.3, 129.7, 128.9, 119.1, 118.8, 115.4, 21.0 ppm.

MS (ESI-(+)): m/z: calculated for $C_{15}H_{15}N_3O_2S$: 301.09, $[M+H]^+$; found: 302.08.

2.2.8. Characterization data for compound 62

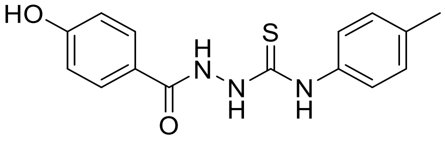

^{1}H-NMR (500 MHz, DMSO), δ: 10.23 (s, 1H), 10.06 (s, 1H), 9.65 (s, 1H), 9.53 (s, 1H), 7.81 (d, *J*= 8.7 Hz, 2H), 7.29 (s, 2H), 7.11 (d, *J*= 8.2 Hz, 2H), 6.81 (d, *J*= 8.2 Hz, 2H), 2.27 (s, 3H) ppm.

^{13}C-NMR (126 MHz, DMSO), δ: 160.6, 136.6, 130.3, 128.8, 123.3, 115.4, 21.1 ppm.

MS (ESI-(+)): m/z: calculated for $C_{15}H_{15}N_3O_2S$: 301.09, $[M+H]^+$; found: 302.08.

2.2.9. Characterization data for compound 63

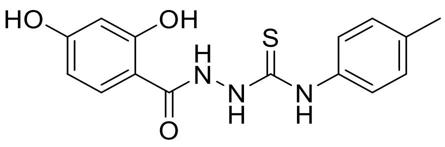

^{1}H-NMR (500 MHz, DMSO), δ: 12.21 (s, 1H), 10.46 (s, 1H), 10.19 (s, 1H), 9.67 (s, 2H), 7.74 (d, *J*= 8.7 Hz, 1H), 7.32 (br s, 2H), 7.13 (d, *J*= 8.1 Hz, 2H), 6.34 (dd, *J*= 8.6 and 2.3 Hz, 1H), 6.30 (d, *J*= 2.0 Hz, 1H), 2.28 (s, 3H) ppm.

^{13}C-NMR (126 MHz, DMSO), δ: 162.3, 137.1, 130.5, 129.3, 128.8, 124.2, 107.5, 103.1, 20.9 ppm.

MS (ESI-(+)): m/z: calculated for $C_{15}H_{15}N_3O_3S$: 317.08, $[M+H]^+$; found: 318.08.

2.2.10. Characterization data for compound 64

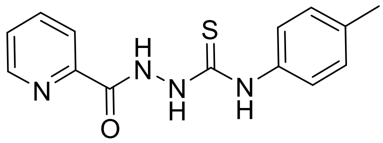

^{1}H-NMR (500 MHz, DMSO), δ: 10.71 (s, 1H), 9.66 (br s, 2H), 8.69 (ddd, *J*= 4.8, 1.6 and 1.0 Hz, 1H), 8.09-7.98 (m, 2H), 7.65 (ddd, *J*= 7.4, 4.5 and 1.5 Hz, 1H), 7.33 (d, *J*= 6.5 Hz, 2H), 7.12 (d, *J*= 8.2 Hz, 2H), 2.28 (s, 3H) ppm.

^{13}C-NMR (126 MHz, DMSO), δ: 148.7, 137.9, 137.0, 134.3, 128.9, 127.2, 123.0, 20.9 ppm.

MS (ESI-(+)): m/z: calculated for $C_{14}H_{14}N_4OS$: 286.09, $[M+H]^+$; found: 287.09.

2.2.11. Characterization data for compound 65

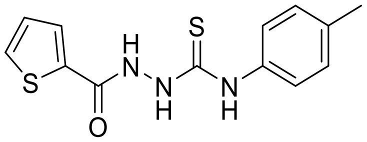

^{1}H-NMR (500 MHz, DMSO), δ: 10.53 (s, 1H), 9.79 (s, 1H), 9.66 (s, 1H), 7.86 (dd, *J*= 7.2 and 2.3 Hz, 2H), 7.29 (t, *J*= 14.4 Hz, 2H), 7.20 (dd, *J*= 5.0 and 3.8 Hz, 1H), 7.13 (d, *J*= 8.2 Hz, 2H), 2.29 (s, 3H) ppm.

^{13}C-NMR (126 MHz, DMSO), δ: 137.9, 137.0, 132.1, 128.9, 128.5, 21.0 ppm.

MS (ESI-(+)): m/z: calculated for $C_{13}H_{13}N_3OS_2$: 291.05, $[M+H]^+$; found: 292.08.

2.2.12. Characterization data for compound 66

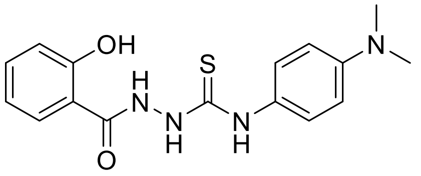

^{1}H-NMR (500 MHz, DMSO), δ: 11.92 (s, 1H), 10.77 (s, 1H), 9.65 (s, 2H), 7.89 (d, *J*= 7.3 Hz, 1H), 7.48-7.40 (m, 1H), 7.19 (d, *J*= 8.6 Hz, 2H), 6.98-6.89 (m, 2H), 6.69 (d, *J*= 8.9 Hz, 2H), 2.88 (s, 6H) ppm.

^{13}C-NMR (126 MHz, DMSO), δ: 148.7, 134.3, 129.2, 119.3, 117.2, 114.9, 112.5, 40.8 ppm.

MS (ESI-(+)): m/z: calculated for $C_{16}H_{18}N_4O_2S$: 330.12, $[M+H]^+$; found: 331.12.

2.2.13. Characterization data for compound 67

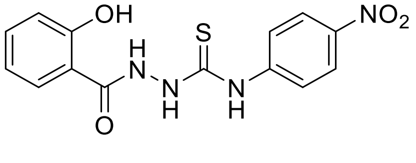

^{1}H-NMR (500 MHz, DMSO), δ: 11.84 (s, 1H), 10.57 (br, 1H), 10.19 (d, *J*= 18.8 Hz, 1H), 8.22 (d, *J*= 9.2 Hz, 2H), 7.92 (s, 3H), 7.46 (t, *J*= 7.4 Hz, 1H), 6.97 (dt, *J*= 13.9 and 7.8 Hz, 2H) ppm.

^{13}C-NMR (126 MHz, DMSO), δ: 134.5, 129.0, 125.1, 123.9, 117.6 ppm.

MS (ESI-(+)): m/z: calculated for $C_{14}H_{12}N_4O_4S$: 332.06, $[M+H]^+$; found: 333.06.

2.2.14. Characterization data for compound 68

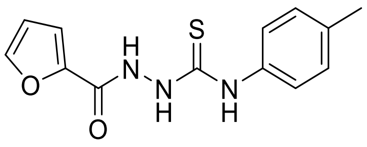

^{1}H-NMR (500 MHz, DMSO), δ: 10.40 (s, 1H), 9.73 (s, 1H), 9.61 (s, 1H), 7.91 (dd, *J*= 1.7 and 0.7 Hz, 1H), 7.29 (s, 2H), 7.24 (d, *J*= 3.3 Hz, 1H), 7.12 (d, *J*= 8.2 Hz, 2H), 6.67 (dd, *J*= 3.5 and 1.7 Hz, 1H), 2.28 (s, 3H) ppm.

^{13}C-NMR (126 MHz, DMSO), δ: 146.9, 146.0, 137.1, 128.8, 115.2, 112.2, 20.8 ppm.

MS (ESI-(+)): m/z: calculated for $C_{13}H_{13}N_3O_2S$: 275.07, $[M+H]^+$; found: 276.03.

2.2.15. Characterization data for compound 69

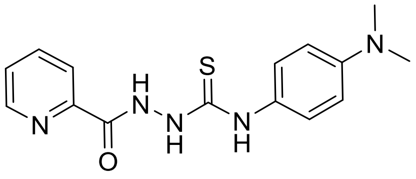

^{1}H-NMR (500 MHz, DMSO), δ: 10.66 (s, 1H), 9.55 (br s, 2H), 8.72-8.65 (m, 1H), 8.11-7.99 (m, 2H), 7.64 (ddd, *J*= 7.3, 4.8, 1.4 Hz, 1H), 7.19 (d, *J*= 8.6 Hz, 2H), 6.67 (d, *J*= 9.0 Hz, 2H), 2.88 (s, 6H) ppm.

^{13}C-NMR (126 MHz, DMSO), δ: 148.8, 148.5, 137.8, 128.8, 127.2, 123.0, 112.3, 40.5 ppm.

MS (ESI-(+)): m/z: calculated for $C_{15}H_{17}N_5OS$: 315.12, $[M+H]^+$; found: 316.10.

2.2.16. Characterization data for compound 70

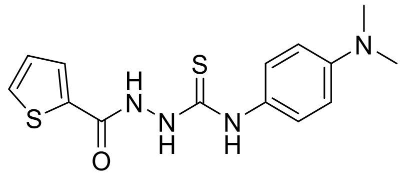

^{1}H-NMR (500 MHz, DMSO), δ: 10.48 (s, 1H), 9.63 (s, 1H), 9.50 (s, 1H), 7.92-7.80 (m, 2H), 7.22-7.12 (m, 3H), 6.67 (d, *J*= 8.9 Hz, 2H), 2.87 (s, 6H) ppm.

^{13}C-NMR (126 MHz, DMSO), δ: 148.7, 137.9, 132.1, 130.1, 128.8, 128.5, 112.3, 41.0 ppm.

MS (ESI-(+)): m/z: calculated for $C_{14}H_{16}N_4OS_2$: 320.08, $[M+H]^+$; found: 321.12.

2.2.17. Characterization data for compound 71

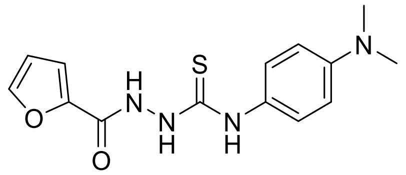

^{1}H-NMR (500 MHz, DMSO), δ: 10.35 (s, 1H), 9.59 (s, 1H), 9.45 (s, 1H), 7.90 (d, *J*= 0.9 Hz, 1H), 7.24 (t, *J*= 4.9 Hz, 1H), 7.16 (d, *J*= 8.6 Hz, 2H), 6.68 (s,1H), 6.67 (dd, *J*= 3.3 and 1.8 Hz, 2H), 2.88 (s, 6H) ppm.

^{13}C-NMR (126 MHz, DMSO), δ: 148.8, 147.3, 146.6, 128.6, 127.3, 114.9, 112.1, 40.8 ppm.

MS (ESI-(+)): m/z: calculated for $C_{14}H_{16}N_4O_2S$: 304.12, $[M+H]^+$; found: 305.12.

2.2.18. Characterization data for compound 72

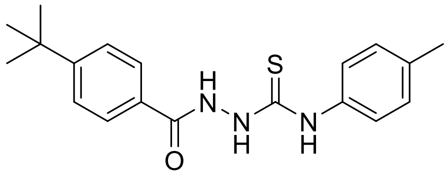

^{1}H-NMR (500 MHz, DMSO), δ: 10.45 (s, 1H), 9.67 (s, 2H), 7.90 (d, *J*= 8.2 Hz, 2H), 7.52 (d, *J*= 8.4 Hz, 2H), 7.31 (s, 2H), 7.13 (d, *J*= 8.0 Hz, 2H), 2.28 (s, 3H), 1.30 (s, 9H) ppm.

^{13}C-NMR (126 MHz, DMSO), δ: 155.2, 137.1, 130.1, 129.2, 128.2, 125.5, 31.9, 20.9 ppm.

MS (ESI-(+)): m/z: calculated for $C_{19}H_{23}N_3OS$: 341.16, $[M+H]^+$; found: 342.24.

2.2.19. Characterization data for compound 73

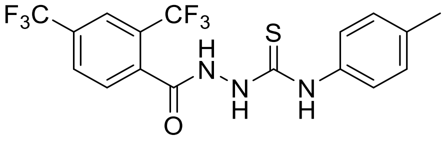

^{1}H-NMR (500 MHz, DMSO), δ: 11.06 (s, 1H), 9.81 (s, 2H), 8.57 (s, 2H), 8.40 (s, 1H), 8.30 (d, *J*= 6.1 Hz, 1H), 7.37-7.23 (m, 2H), 7.15 (d, *J*= 8.2 Hz, 2H), 2.29 (s, 3H) ppm.

^{13}C-NMR (126 MHz, DMSO), δ: 136.9, 135.4, 131.0, 130.9, 128.9, 125.9, 124.4, 122.4, 20.7 ppm.

MS (ESI-(+)): m/z: calculated for $C_{17}H_{13}F_3N_3OS$: 421.07, $[M+H]^+$; found: 422.18.

2.2.20. Characterization data for compound 74

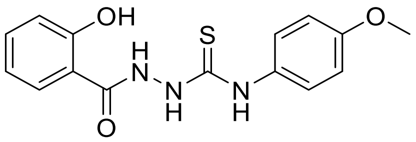

^{1}H-NMR (500 MHz, DMSO), δ: 11.91 (s, 1H), 10.73 (br, 1H), 9.76 (s, 2H), 7.89 (d, *J*= 7.2 Hz, 1H), 7.48-7.42 (m, 1H), 7.31 (d, *J*= 6.3 Hz, 2H), 6.97-6.92 (m, 2H), 6.92-6.88 (m, 2H), 3.75 (s, 3H) ppm.

^{13}C-NMR (126 MHz, DMSO), δ: 157.2, 134.3, 132.4, 129.1, 119.2, 117.7, 115.5, 113.8, 55.7 ppm.

MS (ESI-(+)): m/z: calculated for $C_{15}H_{15}N_3O_3S$: 317.08, $[M+H]^+$; found: 318.11.

2.2.21. Characterization data for compound 75

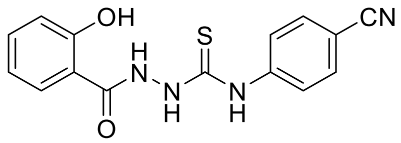

^{1}H-NMR (500 MHz, DMSO), δ: 11.82 (s, 1H), 11.01 (br, 1H), 10.08 (br, 2H), 7.78 (d, *J*= 8.1 Hz, 5H), 7.45 (t, *J*= 7.1 Hz, 1H), 6.95 (d, *J*= 13.9 and 7.9 Hz, 2H) ppm.

^{13}C-NMR (126 MHz, DMSO), δ: 176.6, 134.7, 132.6, 126.4, 119.3, 117.6 ppm.

MS (ESI-(+)): m/z: calculated for $C_{15}H_{12}N_4O_2S$: 312.07, $[M+H]^+$; found: 313.09.

2.2.22. Characterization data for compound 76

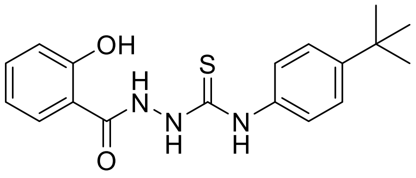

^{1}H-NMR (500 MHz, DMSO), δ: 11.87 (s, 1H), 9.72 (s, 2H), 7.88 (s, 1H), 7.48-7.42 (m, 1H), 7.35 (d, *J*= 8.0 Hz, 4H), 6.95 (dd, *J*= 15.7 and 8.0 Hz, 2H), 1.28 (s, 9H) ppm.

^{13}C-NMR (126 MHz, DMSO), δ: 137.1, 134.3, 129.2, 125.3, 119.5, 117.6, 115.5, 31.6 ppm.

MS (ESI-(+)): m/z: calculated for $C_{18}H_{21}N_3O_2S$: 343.14, $[M+H]^+$; found: 344.22.

2.2.23. Characterization data for compound 77

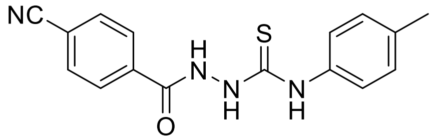

^{1}H-NMR (500 MHz, DMSO), δ: 10.78 (s, 1H), 9.74 (s, 2H), 8.05 (d, *J* = 8.0 Hz, 4H), 7.28 (s, 2H), 7.13 (d, *J*= 7.7 Hz, 2H), 2.28 (s, 3H) ppm.

^{13}C-NMR (126 MHz, DMSO), δ: 137.1, 133.0, 128.9, 118.7, 114.7, 21.3 ppm.

MS (ESI-(+)): m/z: calculated for $C_{16}H_{14}N_4OS$: 310.09, $[M+H]^+$; found: 311.10.

2.2.24. Characterization data for compound 78

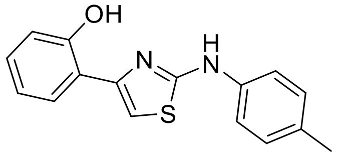

^{1}H-NMR (500 MHz, DMSO), δ: 10.30 (s, 1H), 7.87 (dd, *J* = 1.7 and 8.0 Hz, 1H), 7.42 (d, *J* = 5 Hz, 2H), 7.38 (s, 1H), 7.19-7.15 (m, 3H) 6.91-6.86 (m, 3H), 2.28 (s, 3H) ppm.

MS (ESI-(+)): m/z: calculated for $C_{16}H_{14}N_2OS$: 282.08, $[M+H]^+$; found: 283.00.

2.2.25. Characterization data for compound 79

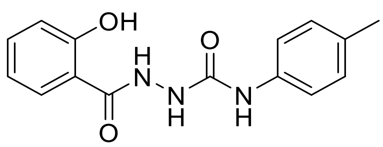

^{1}H-NMR (500 MHz, DMSO), δ: 11.96 (s, 1H), 10.46 (s, 2H), 8.80 (s, 1H), 8.28 (s, 1H), 7.89 (dd, *J*= 7.9 and 1.6 Hz, 1H), 7.49-7.42 (m, 1H), 7.35 (d, *J*= 8.4 Hz, 2H), 7.07 (d, *J*= 8.2 Hz, 2H), 6.94 (dd, *J*= 16.4 and 7.9 Hz, 2H), 2.24 (s, 3H) ppm.

^{13}C-NMR (126 MHz, DMSO), δ: 137.3, 134.3, 131.2, 129.5, 119.3, 119.1, 117.8, 20.4 ppm.

MS (ESI-(+)): m/z: calculated for $C_{15}H_{15}N_3O_3$: 285.11, $[M+H]^+$; found: 286.11.

3. ^{1}H-NMR, ^{13}C- and UPLC spectra for compounds 3, 55-79

3.1 Compound 3

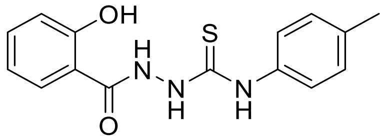

3.1.1 ^{1}H-NMR

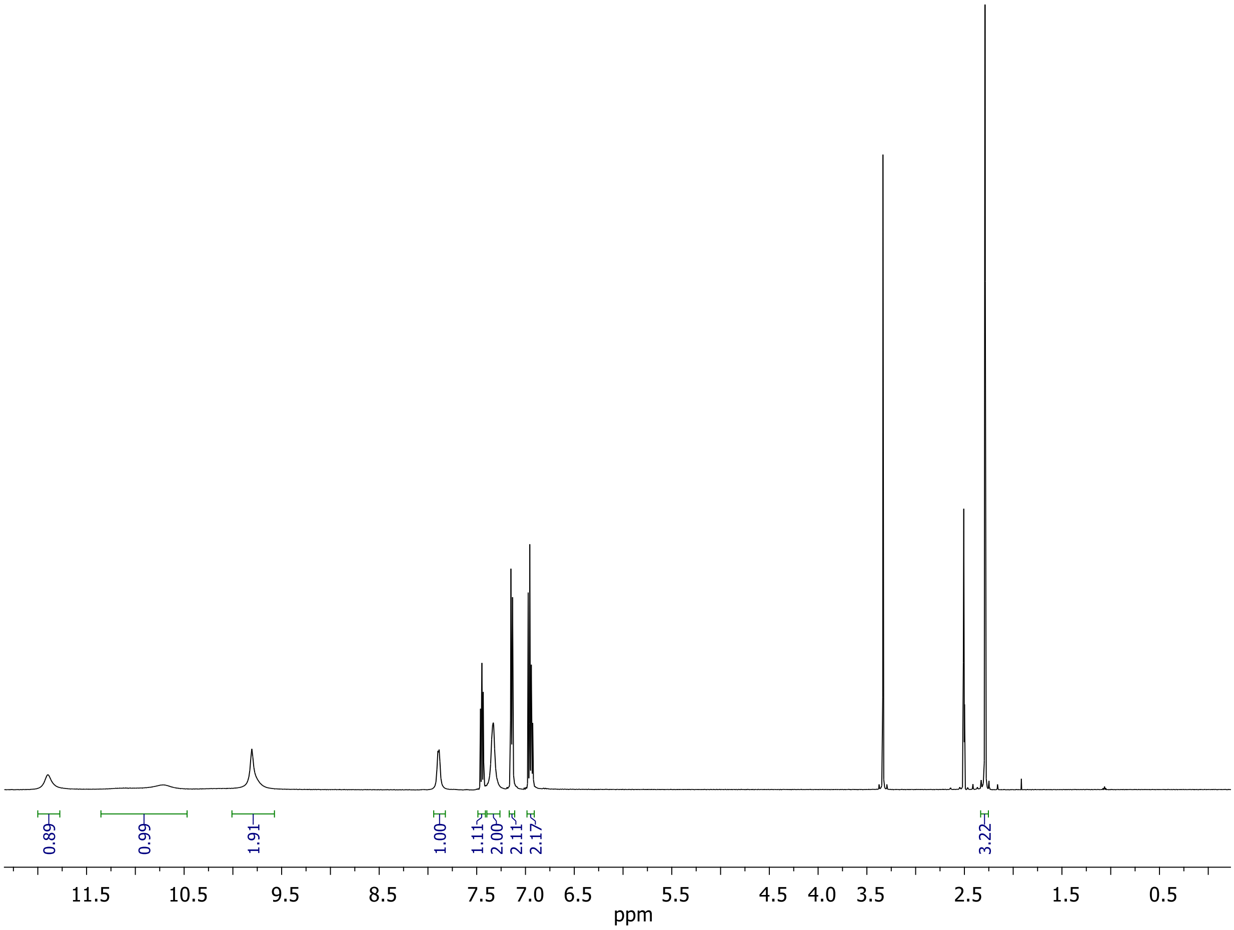

3.1.2 ^{13}C-NMR

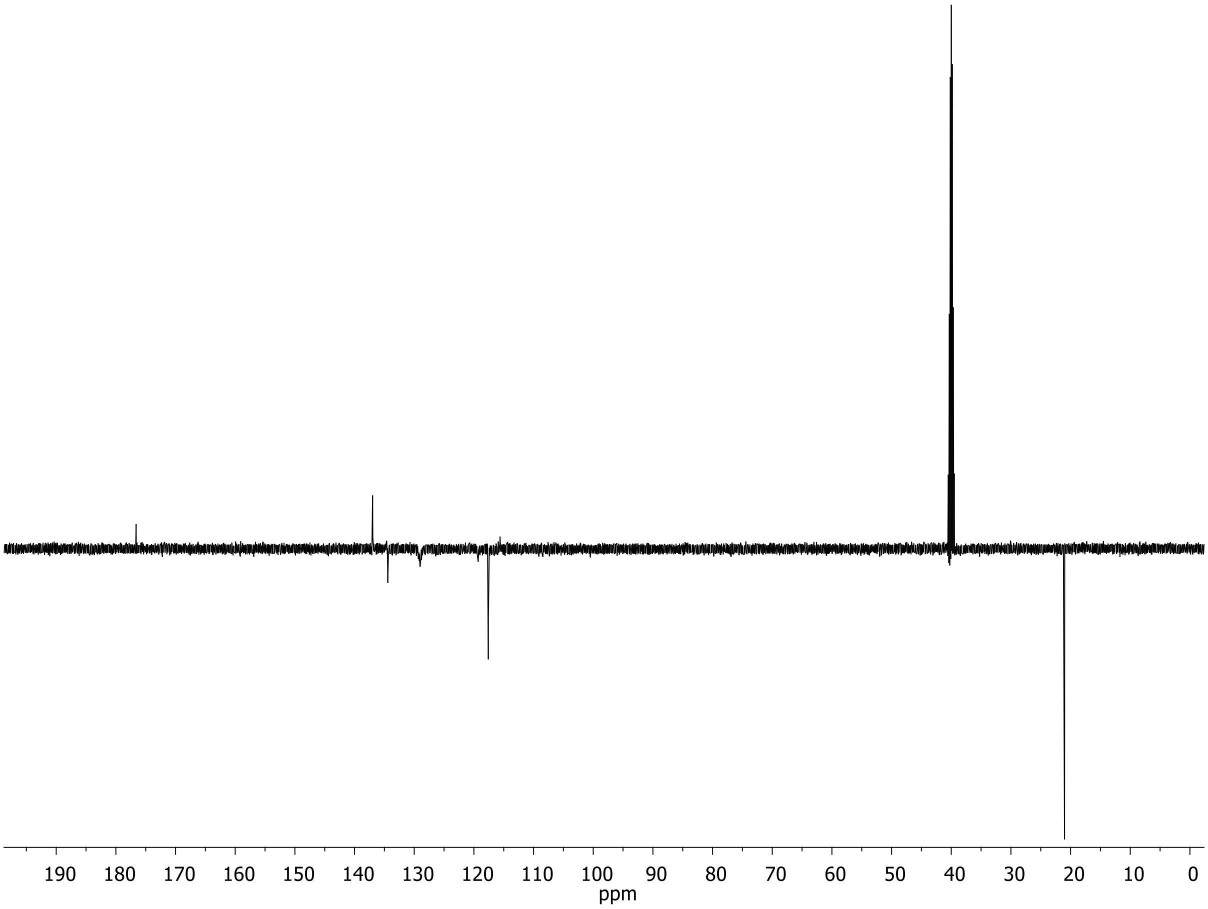
190 180 170 160 150 140 130 120 110 100 90 80 70 60 50 40 30 20 10 0
ppm

3.1.3. UPLC

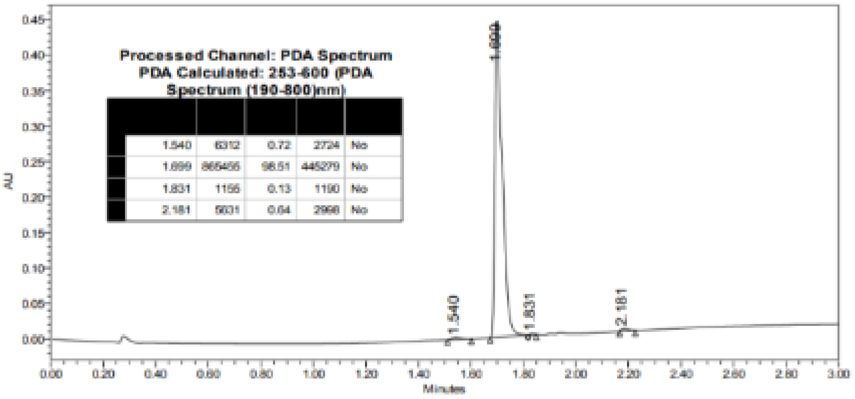
Processed Channel: PDA Spectrum
PDA Calculated: 253-600 (PDA Spectrum (190-800)nm)
1.540 6312 0.72 2724 No
1.699 865455 98.51 445279 No
1.831 1155 0.13 1190 No
2.181 5031 0.64 2998 No
1.699
1.540
1.831
2.181
AU
Minutes

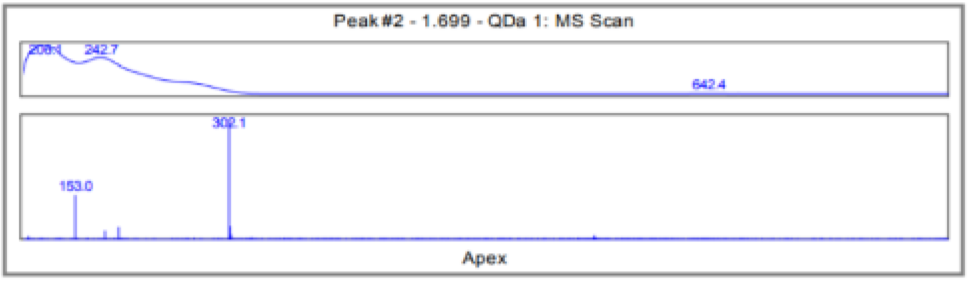
Peak #2 - 1.699 - QDa 1: MS Scan
208.4
242.7
642.4
302.1
153.0
Apex

3.2 Compound 55

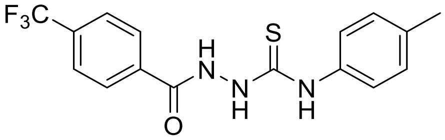

3.2.1 ^{1}H-NMR

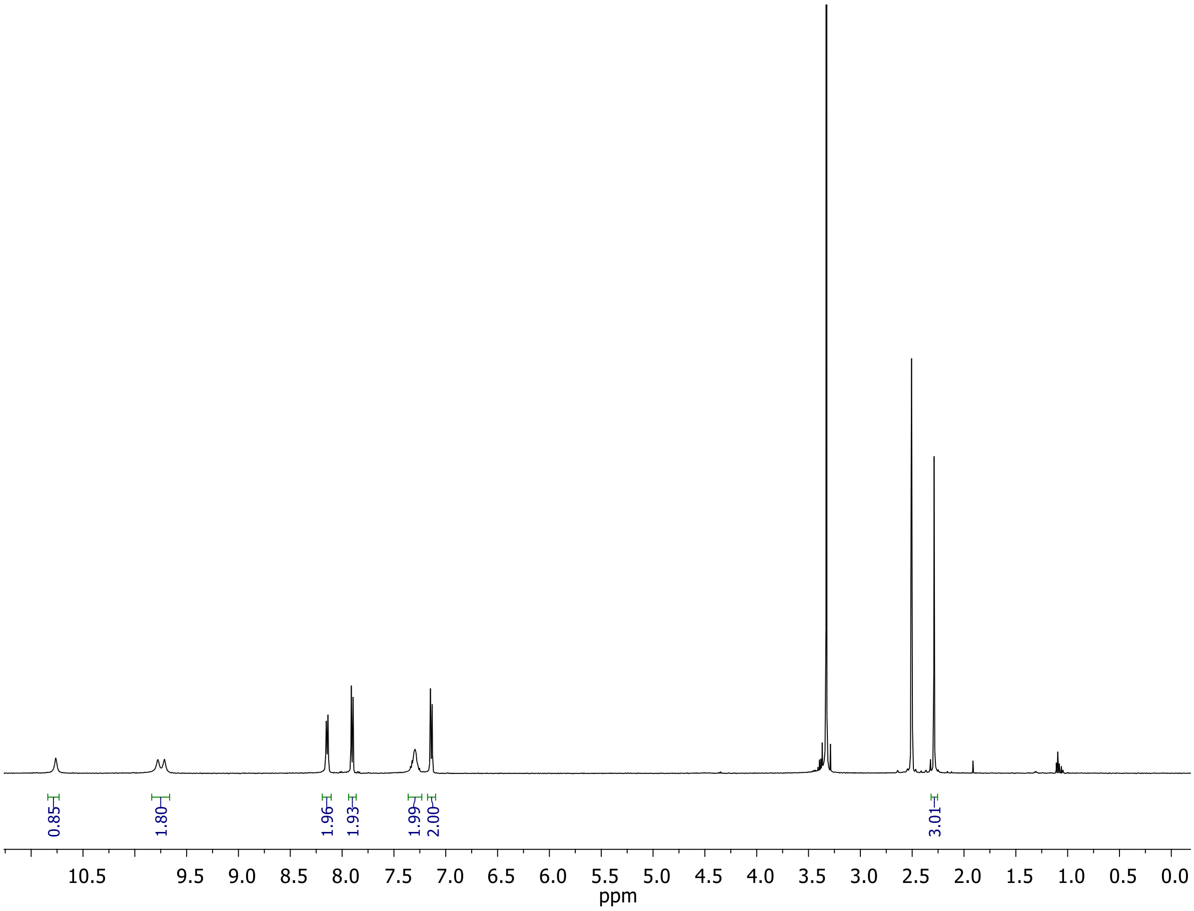

3.2.2 ^{13}C-NMR

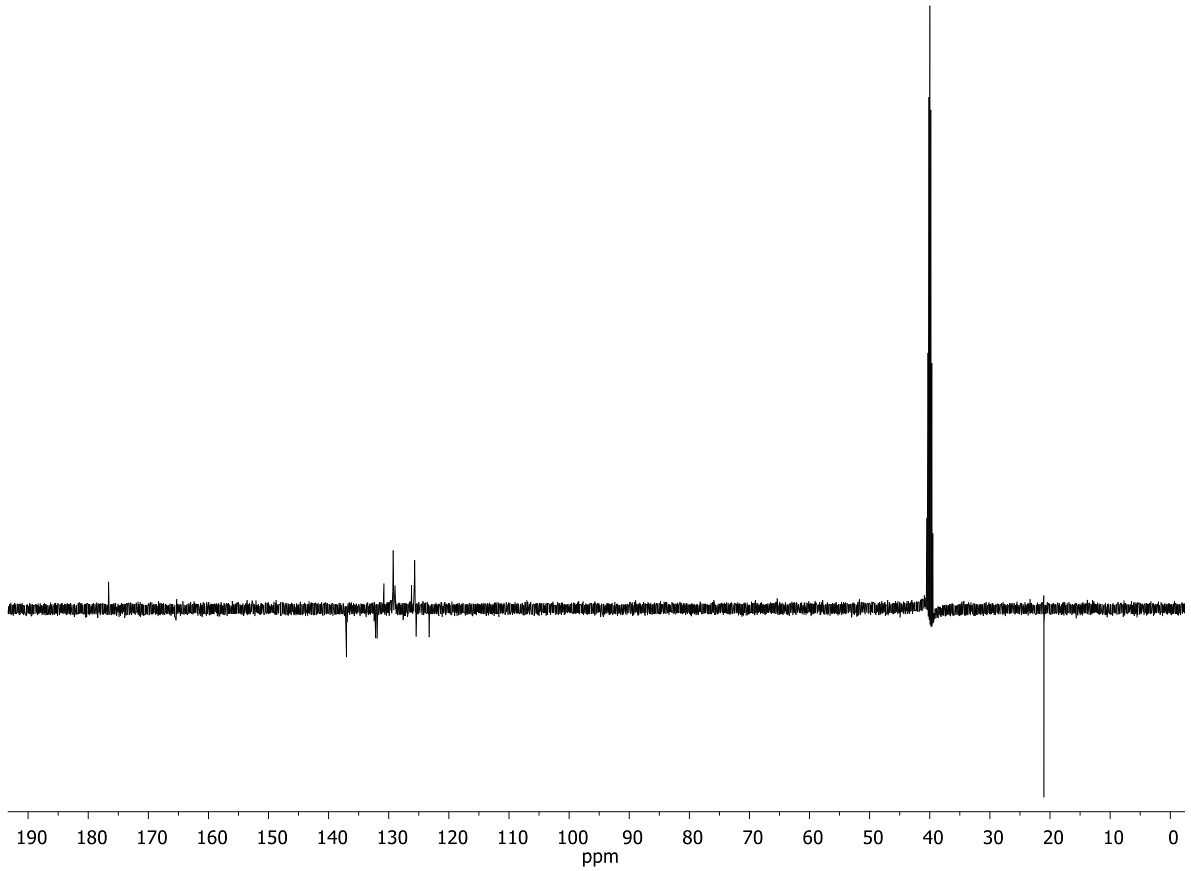
190
180
170
160
150
140
130
120
110
100
90
80
70
60
50
40
30
20
10
0
ppm

3.2.3 UPLC

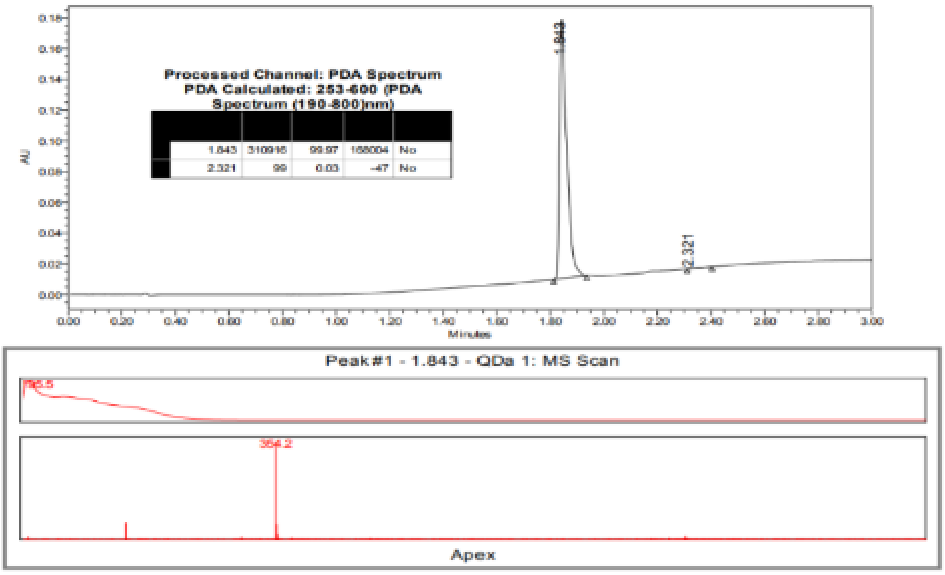

Processed Channel: PDA Spectrum
PDA Calculated: 253-600 (PDA Spectrum (190-800)nm)
1.843 310916 99.97 168004 No
2.321 99 0.03 -47 No
1.843
2.321
AU
Minutes
Peak #1 - 1.843 - QDa 1: MS Scan
354.2
Apex

3.3 Compound 56

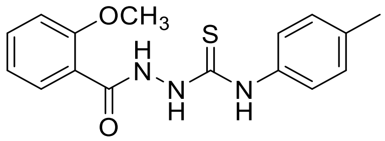

3.3.1 ^{1}H-NMR

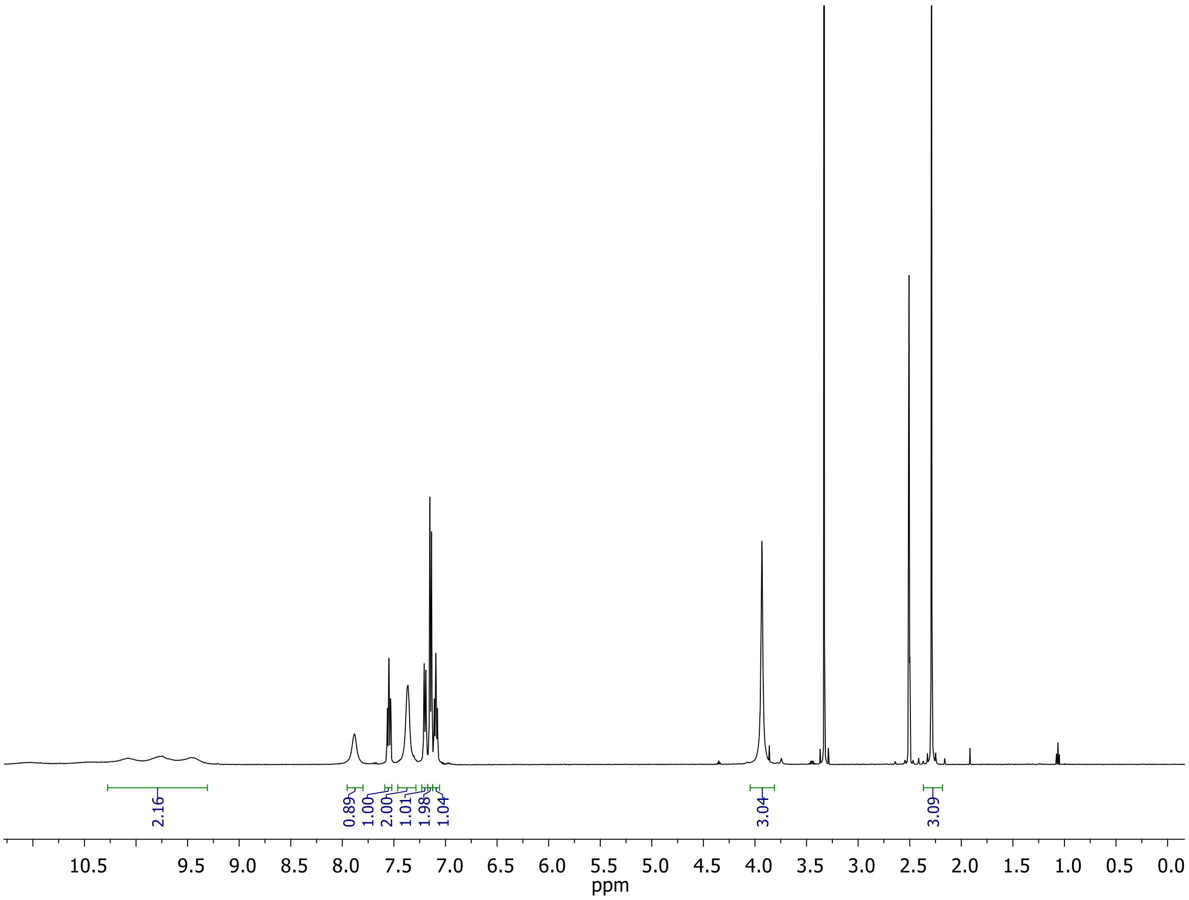

3.3.2 ^{13}C-NMR

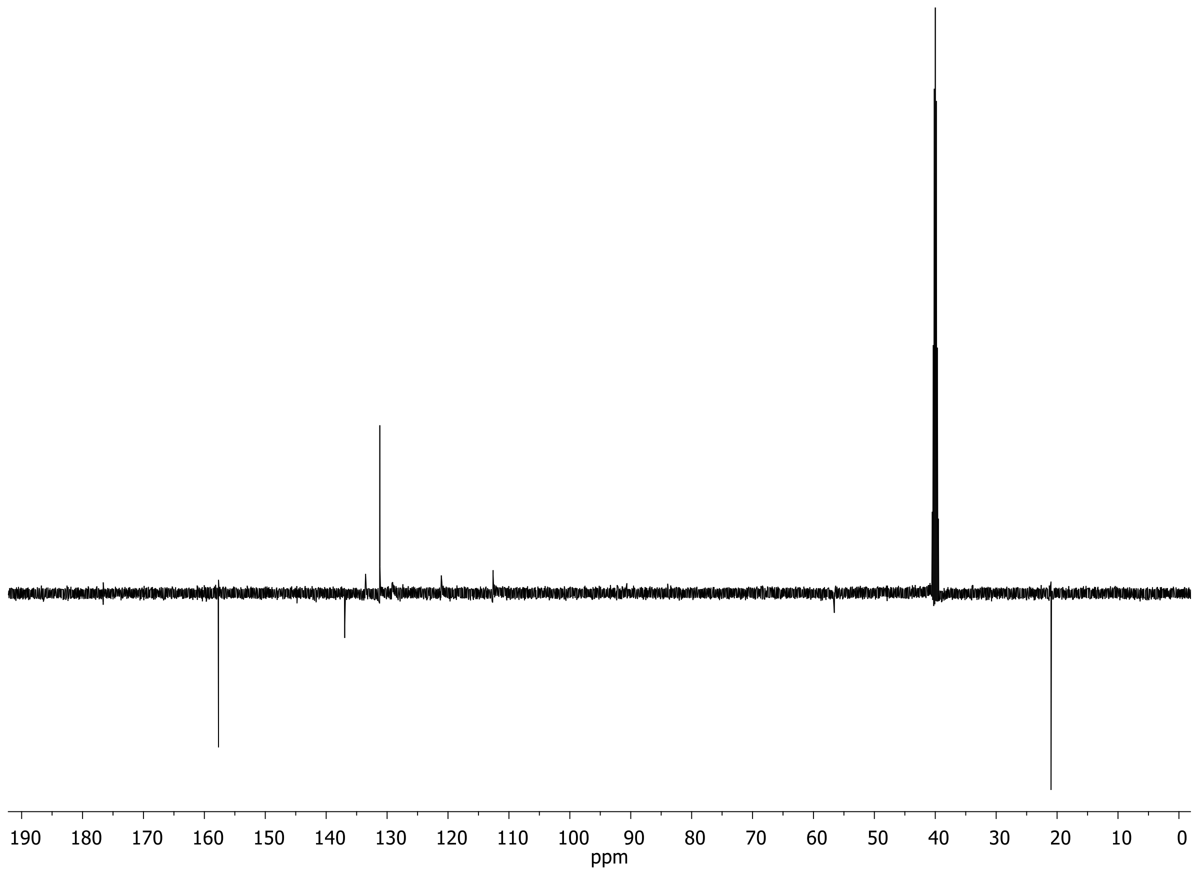

190 180 170 160 150 140 130 120 110 100 90 80 70 60 50 40 30 20 10 0
ppm

3.3.3 UPLC

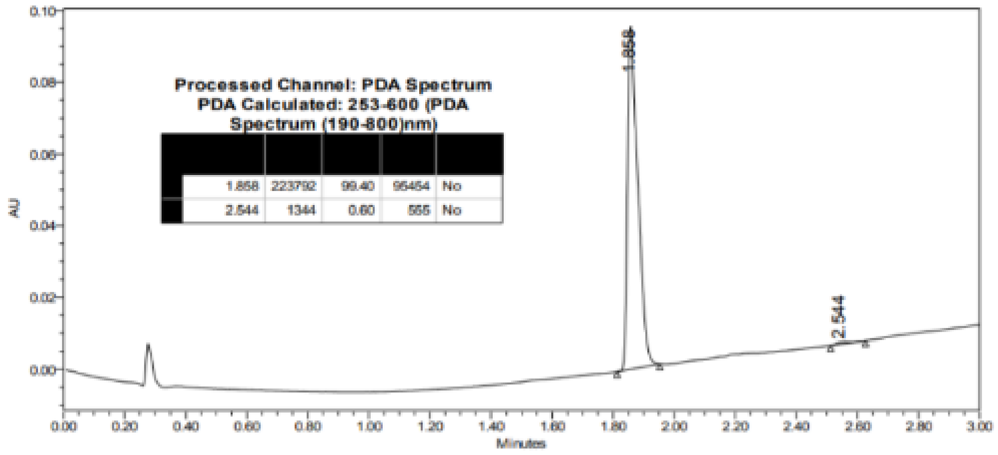
Processed Channel: PDA Spectrum
PDA Calculated: 253-600 (PDA Spectrum (190-800)nm)
1.858 223792 99.40 95454 No
2.544 1344 0.60 555 No
1.858
2.544
AU
Minutes
0.00
0.02
0.04
0.06
0.08
0.10
0.00 0.20 0.40 0.60 0.80 1.00 1.20 1.40 1.60 1.80 2.00 2.20 2.40 2.60 2.80 3.00

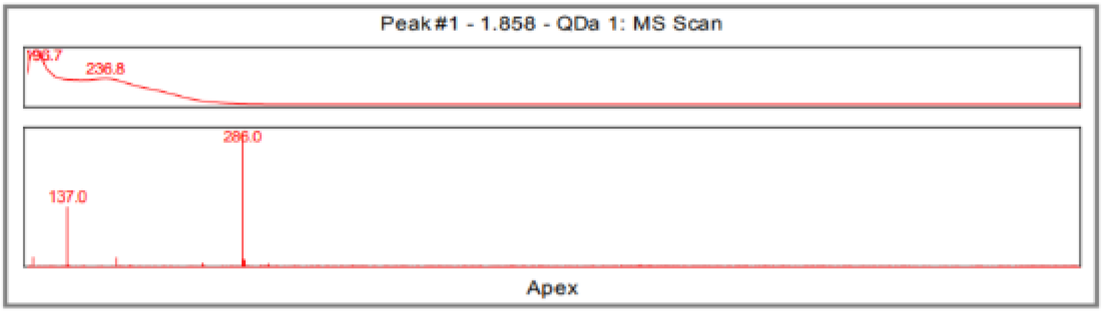
Peak #1 - 1.858 - QDa 1: MS Scan
236.8
286.0
137.0
Apex

3.4 Compound 57

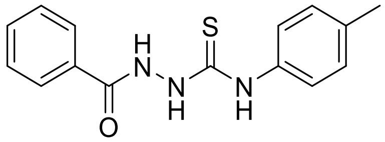

3.4.1 ^{1}H-NMR

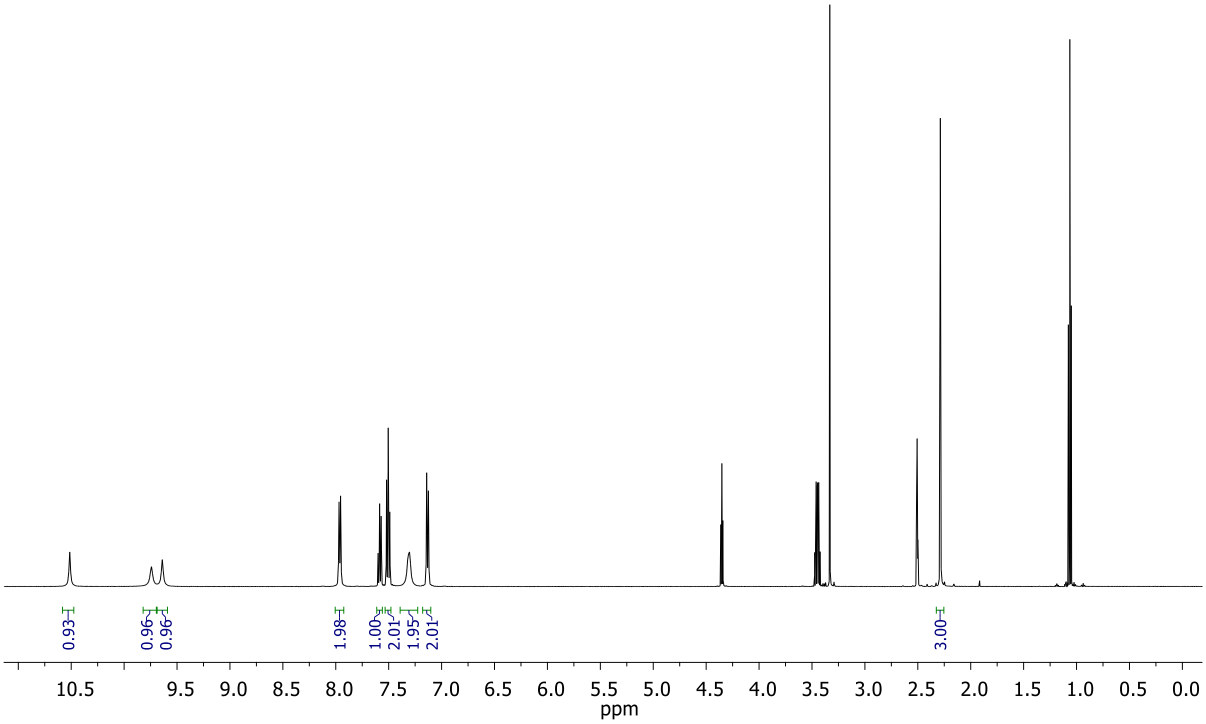

3.4.2 ¹³C-NMR

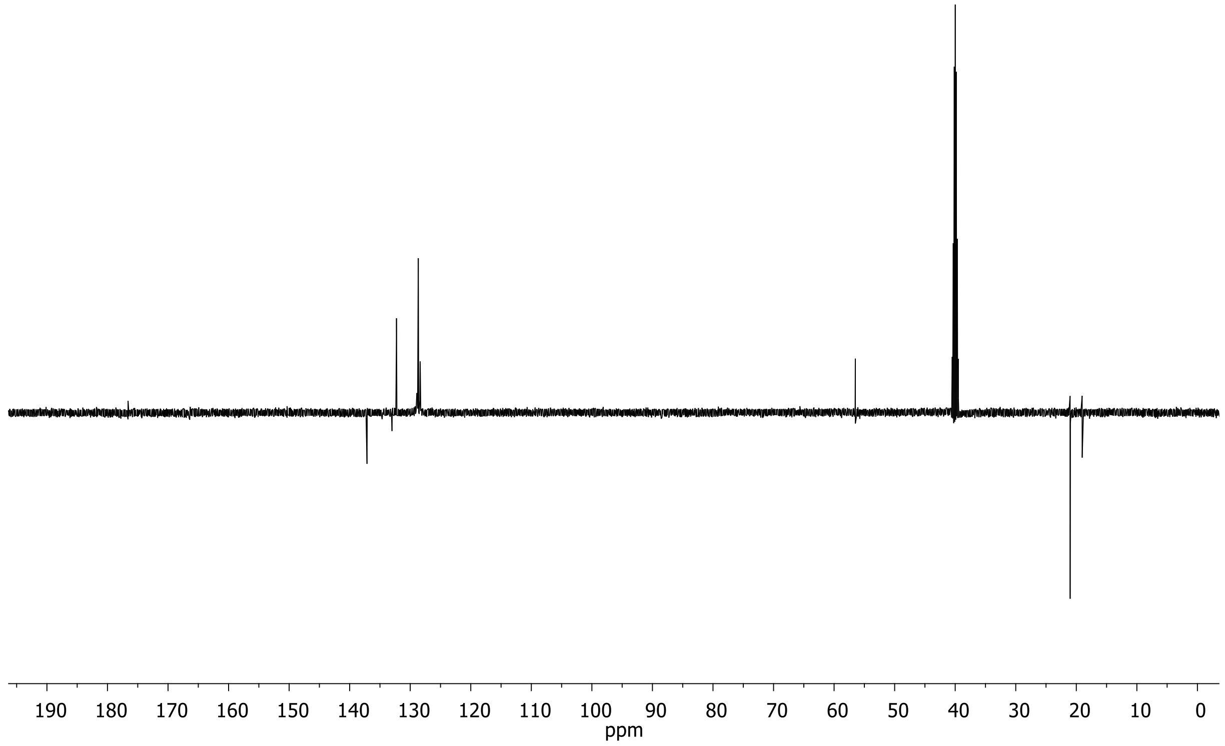
190
180
170
160
150
140
130
120
110
100
90
80
70
60
50
40
30
20
10
0
ppm

3.4.3 UPLC

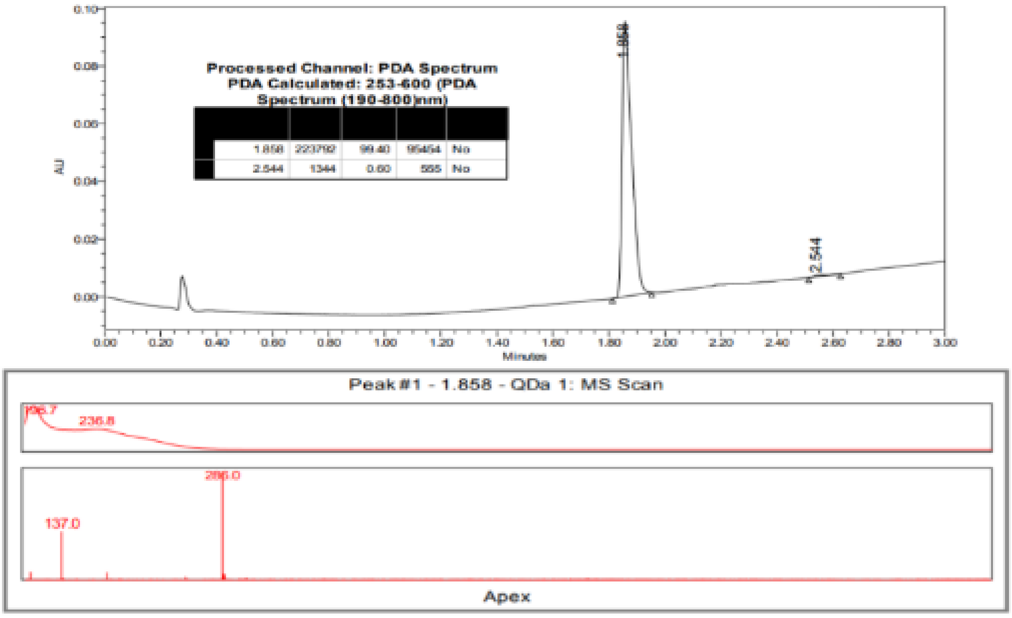
Processed Channel: PDA Spectrum
PDA Calculated: 253-600 (PDA Spectrum (190-800)nm)
1.858 223792 99.40 95454 No
2.544 1344 0.60 555 No
AU
Minutes
1.858
2.544
Peak #1 - 1.858 - QDa 1: MS Scan
236.8
286.0
137.0
Apex

3.5 Compound 58

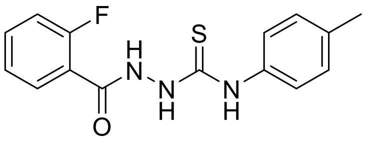

3.5.1 ^{1}H-NMR

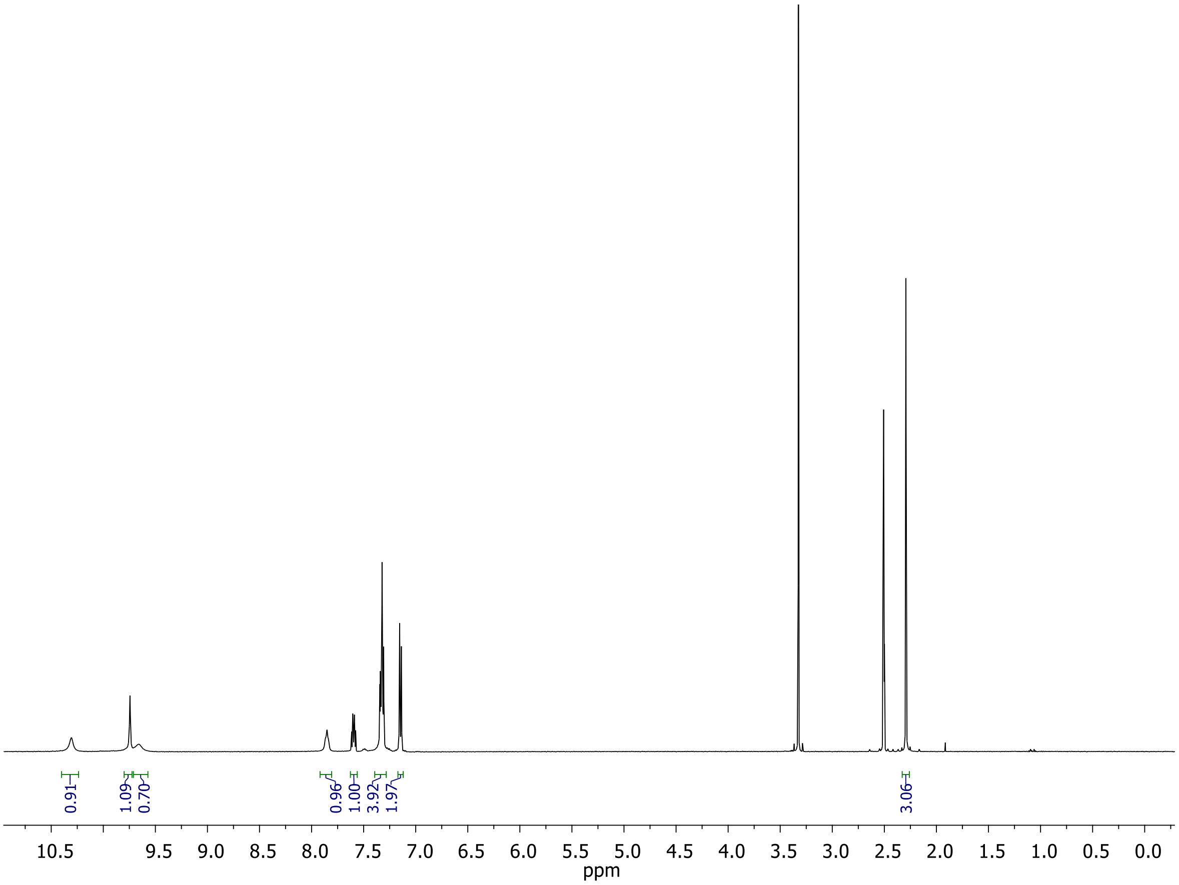

3.5.2 ^{13}C-NMR

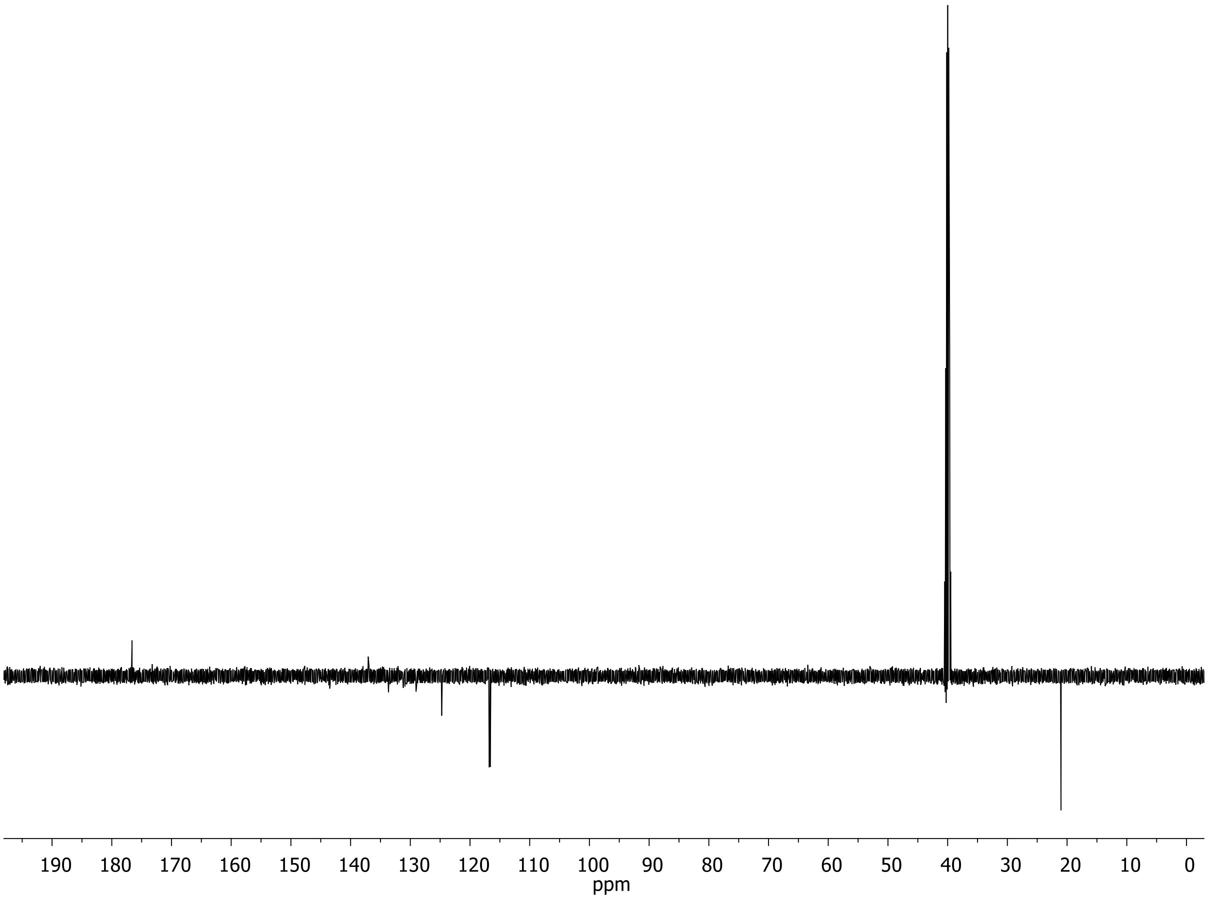
190 180 170 160 150 140 130 120 110 100 90 80 70 60 50 40 30 20 10 0
ppm

3.5.3 UPLC

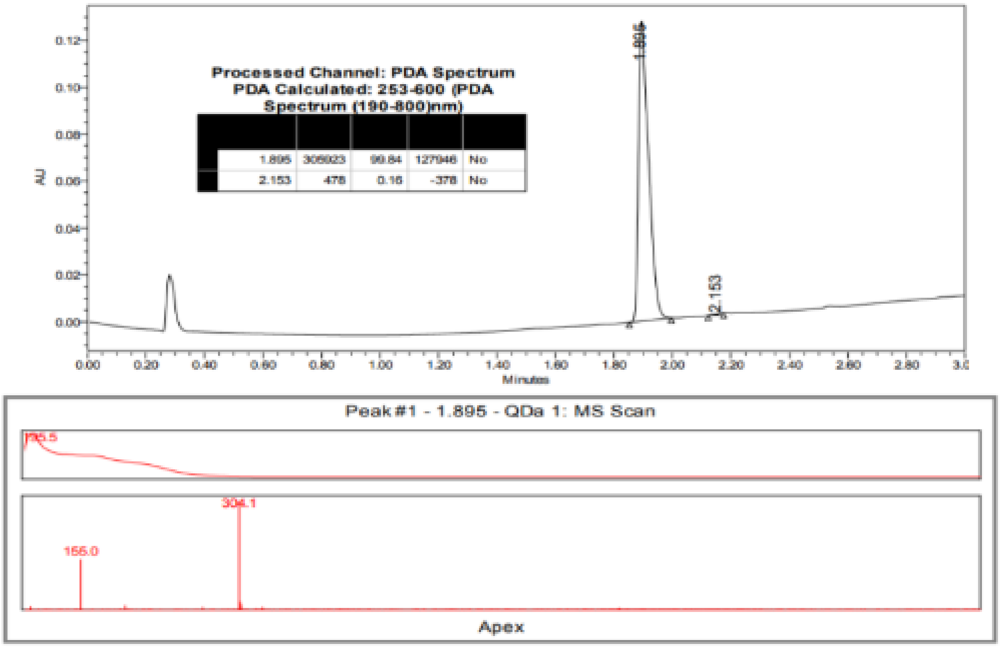
1.895
2.153
Processed Channel: PDA Spectrum
PDA Calculated: 253-600 (PDA
Spectrum (190-800)nm)
1.895 305023 99.84 127946 No
2.153 478 0.16 -378 No
AU
0.12
0.10
0.08
0.06
0.04
0.02
0.00
0.00 0.20 0.40 0.60 0.80 1.00 1.20 1.40 1.60 1.80 2.00 2.20 2.40 2.60 2.80
Minutes
Peak #1 - 1.895 - QDa 1: MS Scan
155.0
Apex

3.6 Compound 59

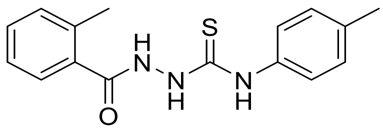

3.6.1 ^{1}H-NMR

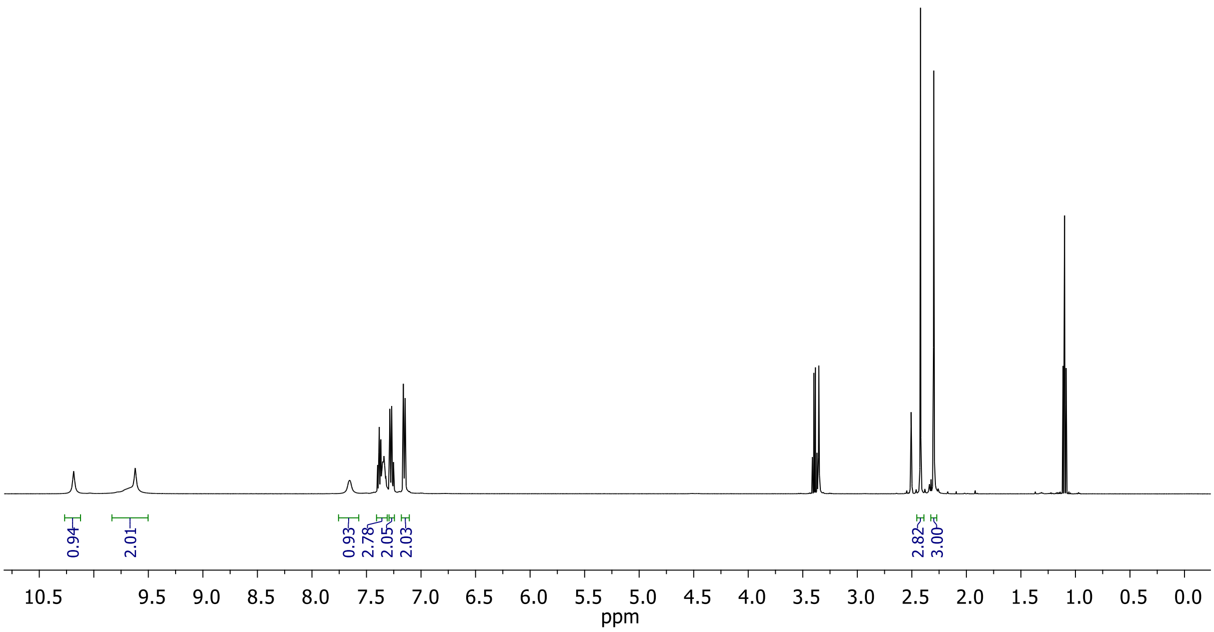

3.6.2 ^{13}C-NMR

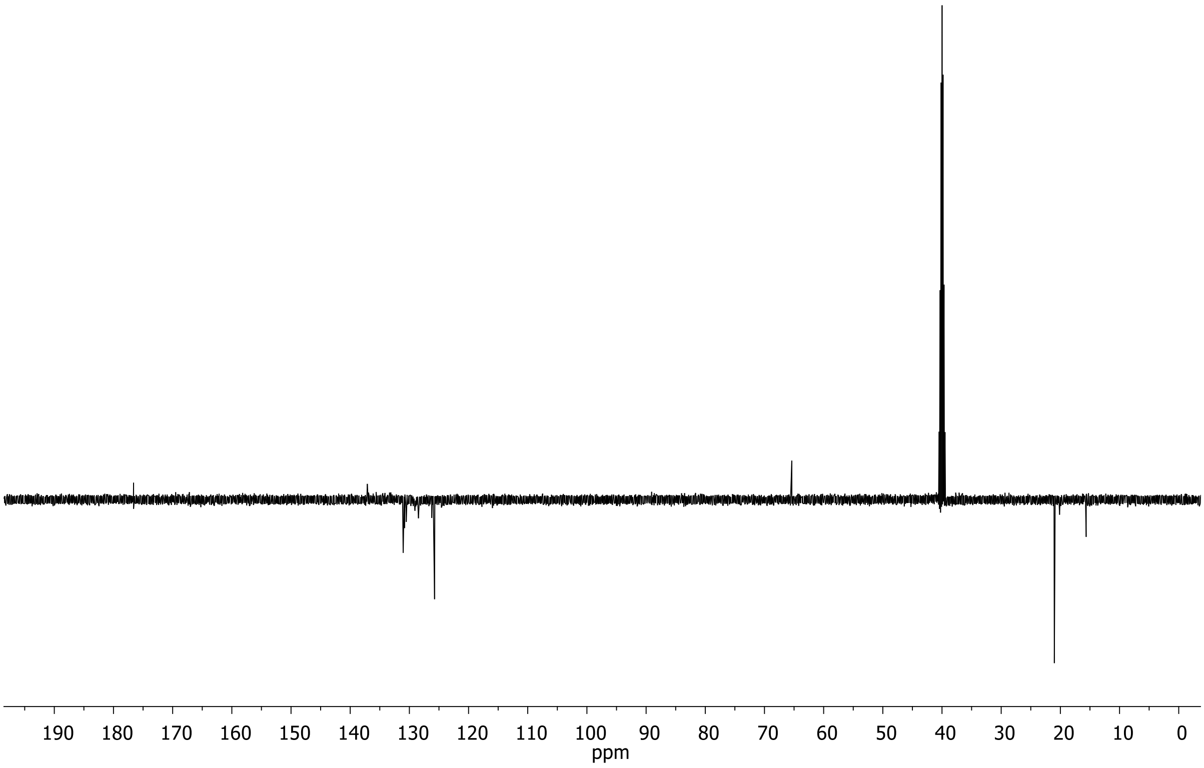
190 180 170 160 150 140 130 120 110 100 90 80 70 60 50 40 30 20 10 0
ppm

3.6.3 UPLC

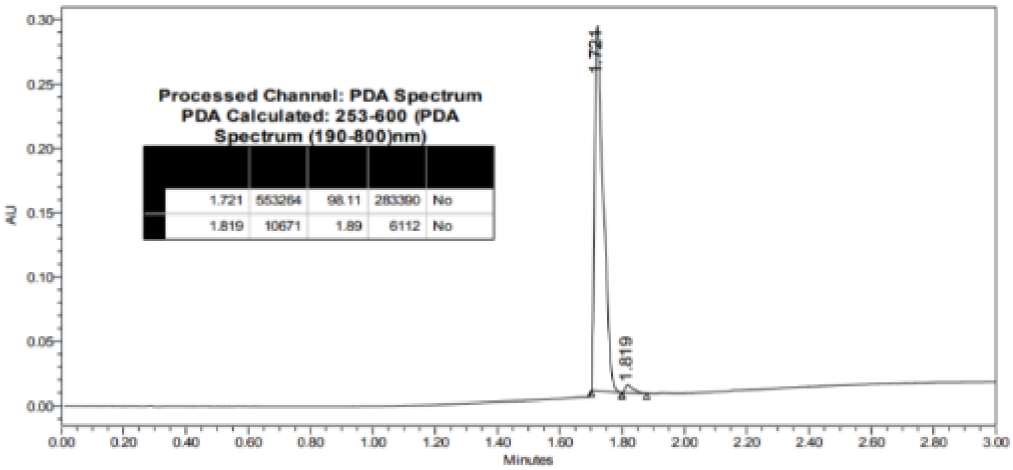
Processed Channel: PDA Spectrum
PDA Calculated: 253-600 (PDA
Spectrum (190-800)nm)
1.721 553264 98.11 283390 No
1.819 10671 1.89 6112 No
1.721
1.819
AU
Minutes

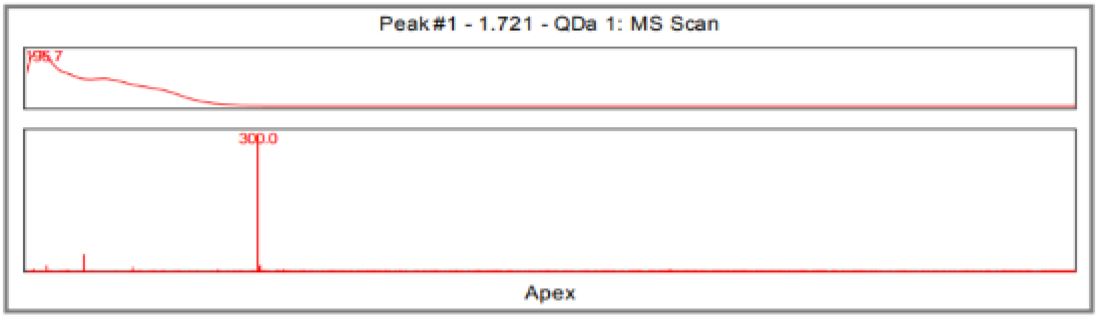
Peak #1 - 1.721 - QDa 1: MS Scan
300.0
Apex

3.7 Compound 60

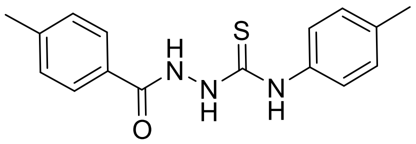

3.7.1 ^{1}H-NMR

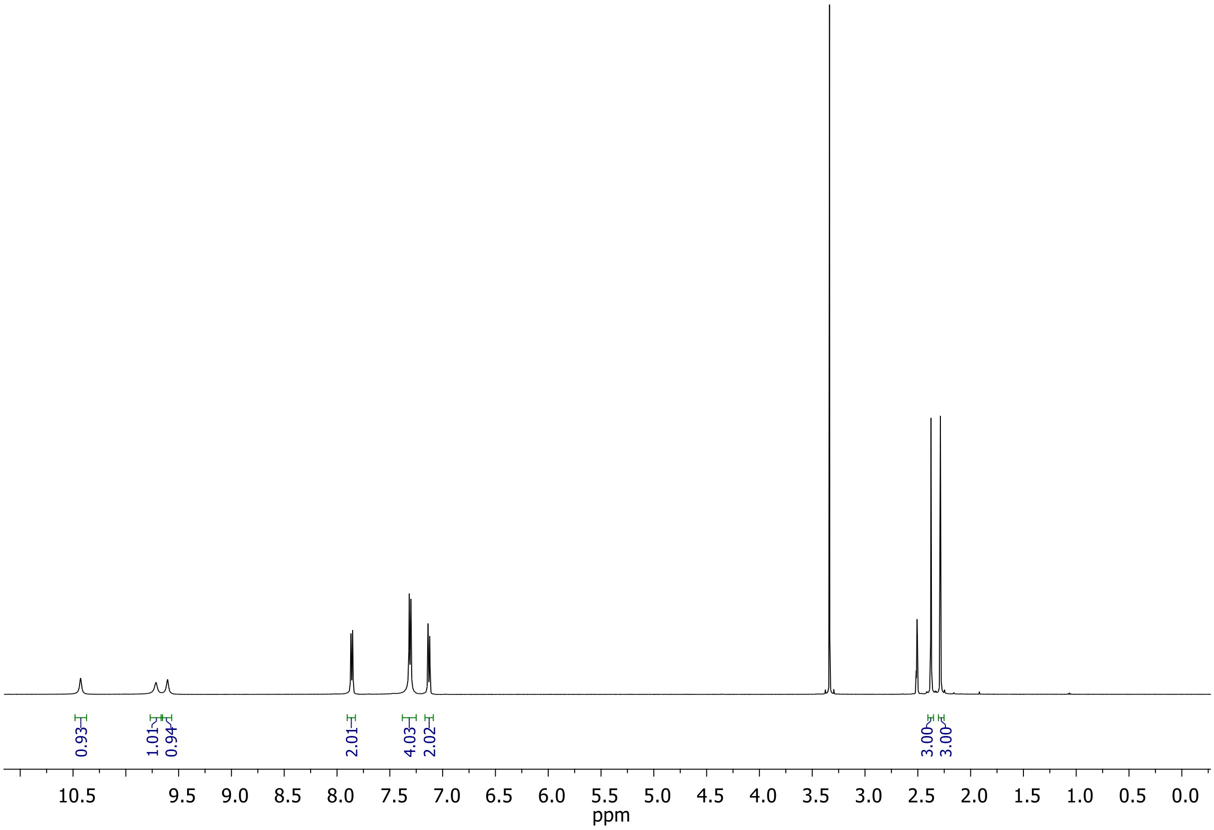

3.7.2 ^{13}C-NMR

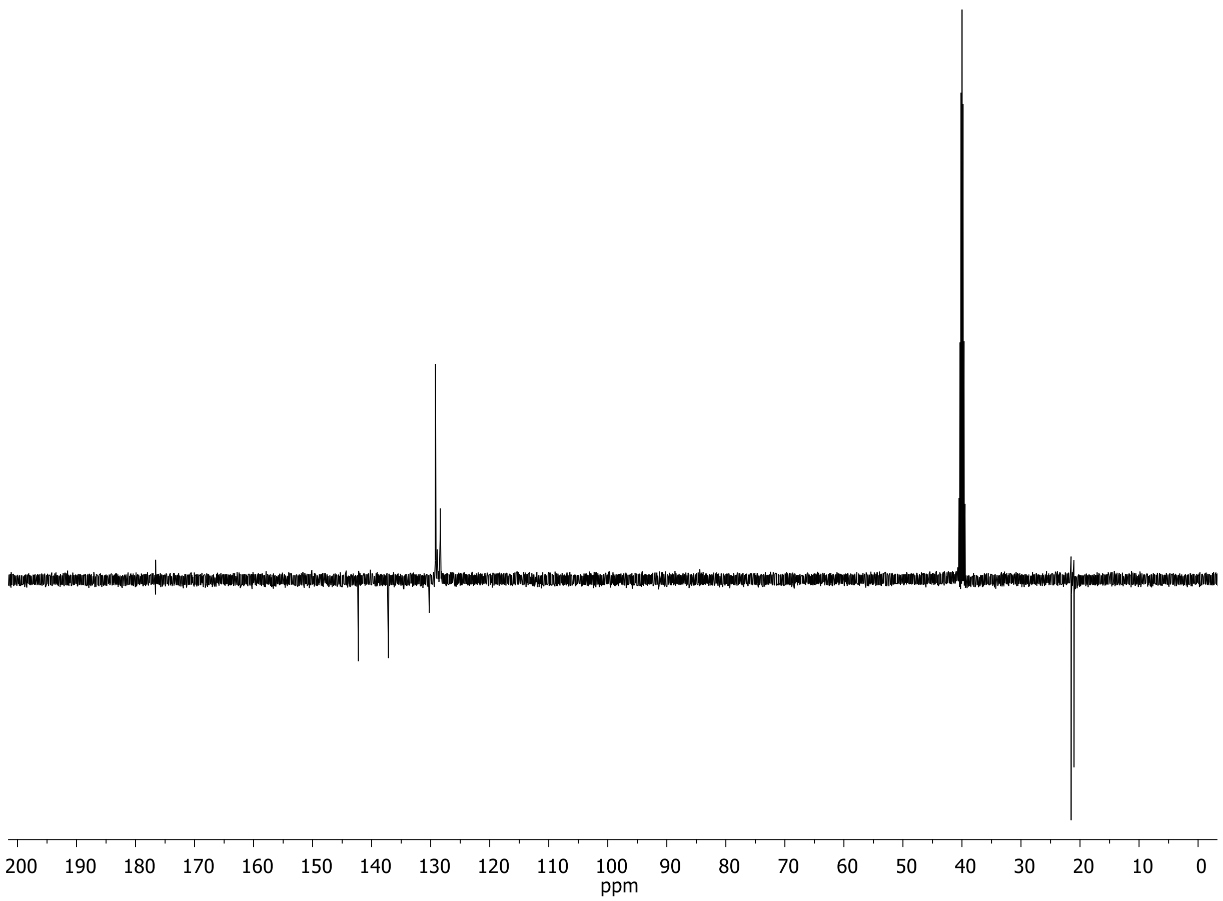
200 190 180 170 160 150 140 130 120 110 100 90 80 70 60 50 40 30 20 10 0
ppm

3.7.3 UPLC

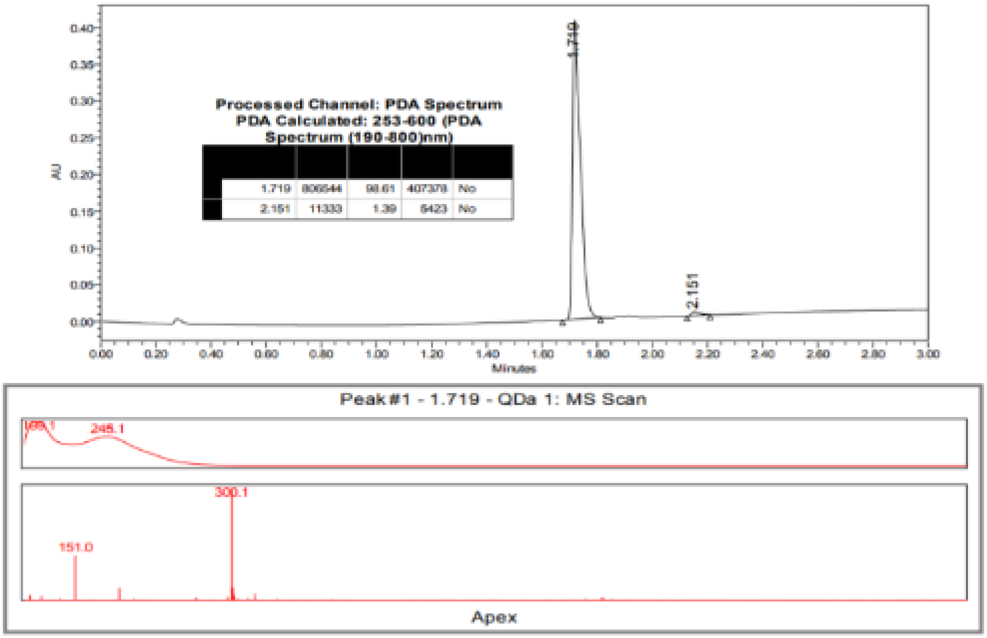
Processed Channel: PDA Spectrum
PDA Calculated: 253-600 (PDA
Spectrum (190-800)nm)
AU
Minutes
2.151
Peak #1 - 1.719 - QDa 1: MS Scan
245.1
300.1
151.0
Apex

3.8 Compound 61

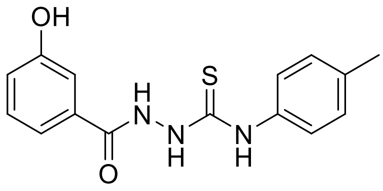

3.8.1 ^{1}H-NMR

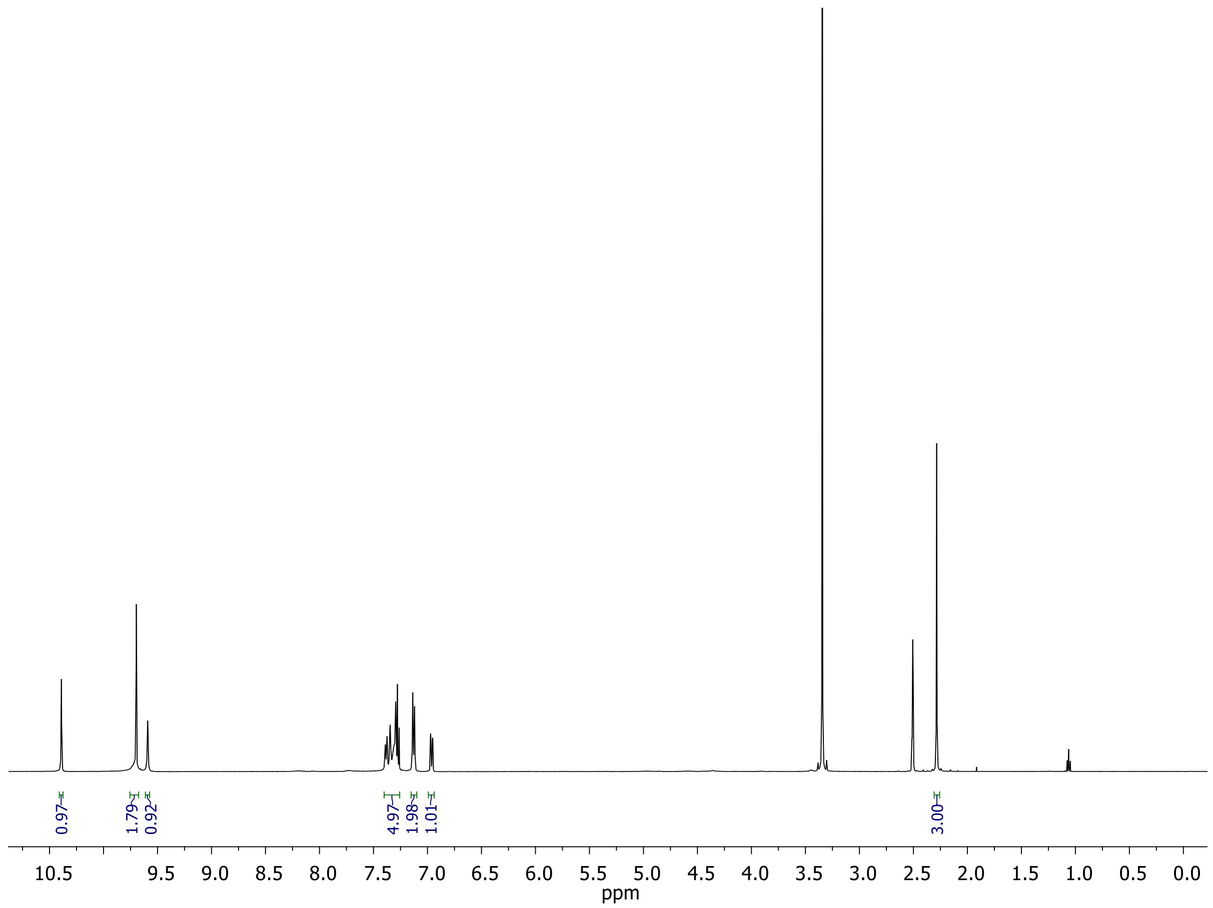

3.8.2 ^{13}C-NMR

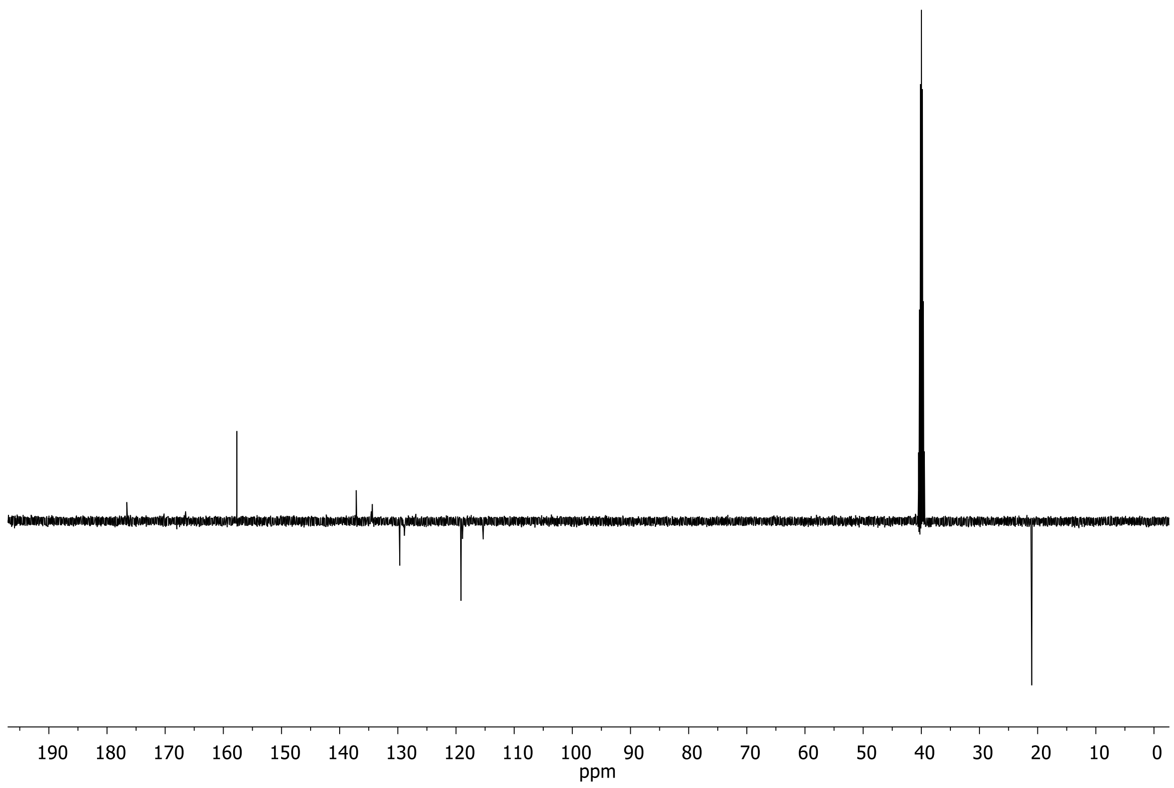
190 180 170 160 150 140 130 120 110 100 90 80 70 60 50 40 30 20 10 0
ppm

3.8.3 UPLC

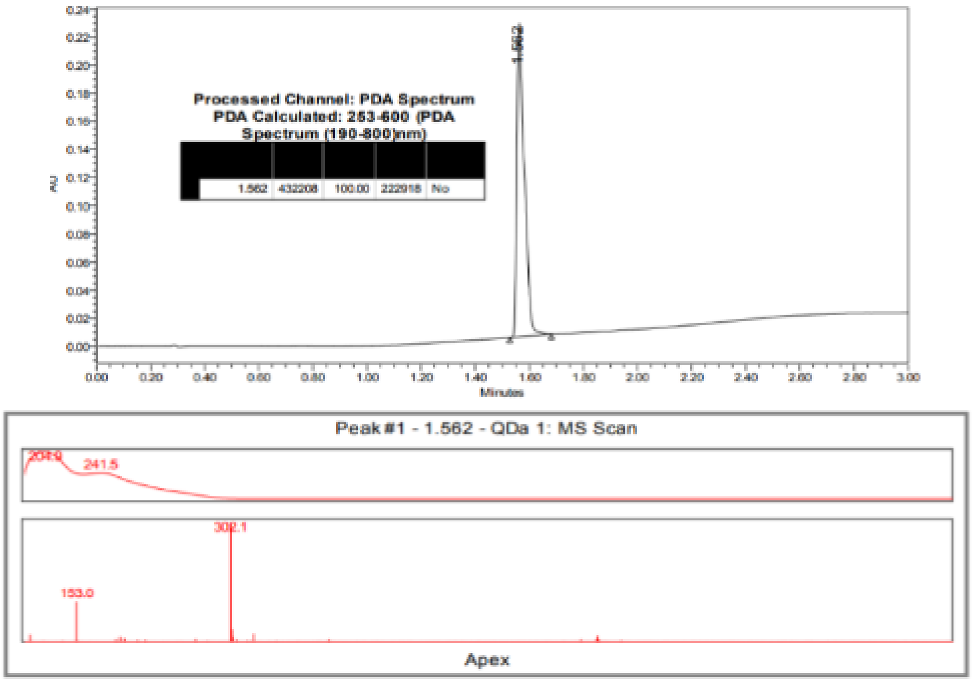
Processed Channel: PDA Spectrum
PDA Calculated: 253-600 (PDA
Spectrum (190-800)nm)
1.562 432208 100.00 222918 No
AU
Minutes
Peak #1 - 1.562 - QDa 1: MS Scan
241.5
153.0
Apex

3.9 Compound 62

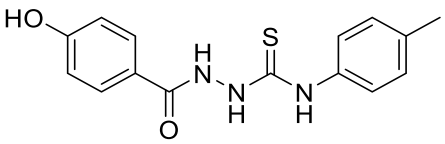

3.9.1 ^{1}H-NMR

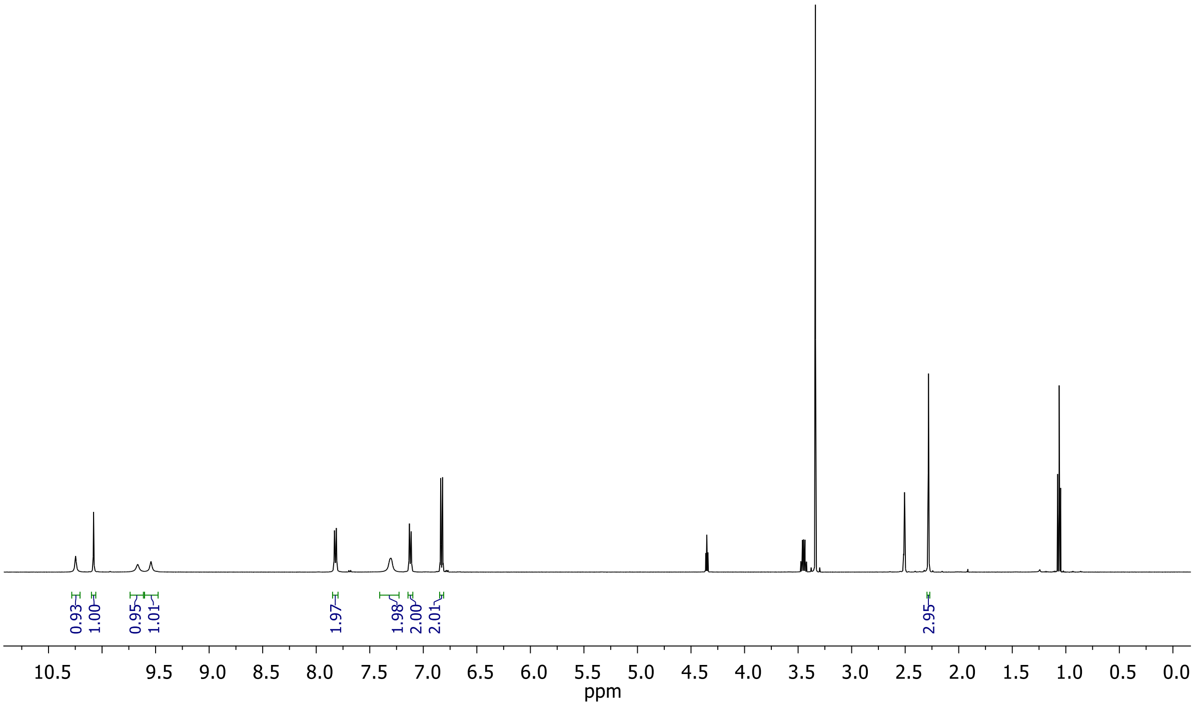

3.9.2 ^{13}C-NMR

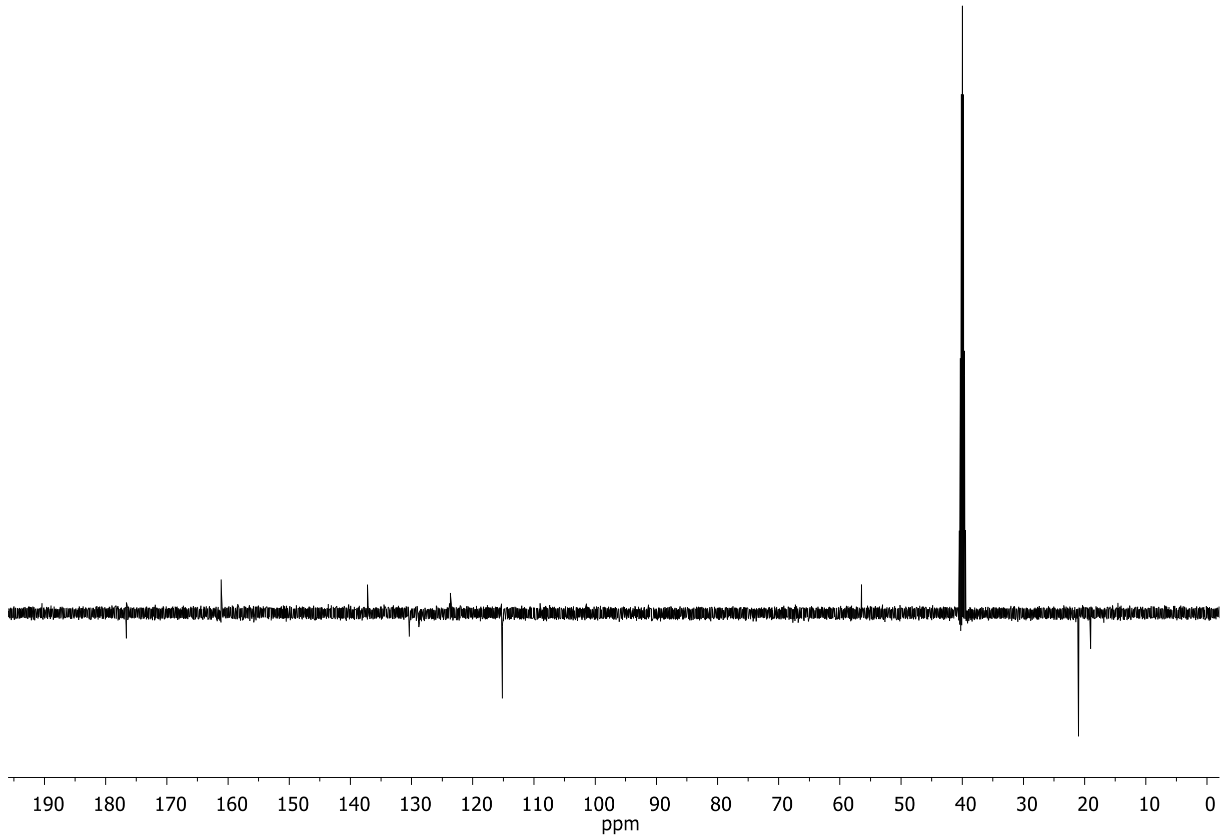
190
180
170
160
150
140
130
120
110
100
90
80
70
60
50
40
30
20
10
0
ppm

3.9.3 UPLC

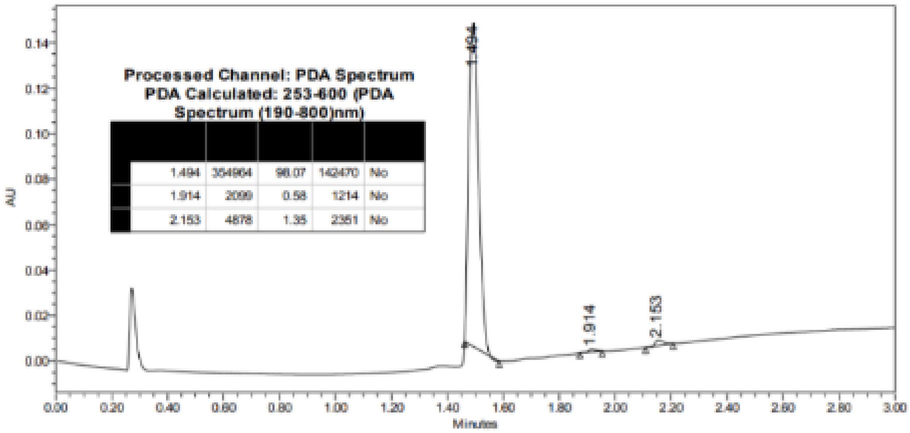
Processed Channel: PDA Spectrum
PDA Calculated: 253-600 (PDA Spectrum (190-800)nm)
1.494 354964 98.07 142470 No
1.914 2099 0.58 1214 No
2.153 4878 1.35 2351 No
1.494
1.914
2.153
AU
Minutes

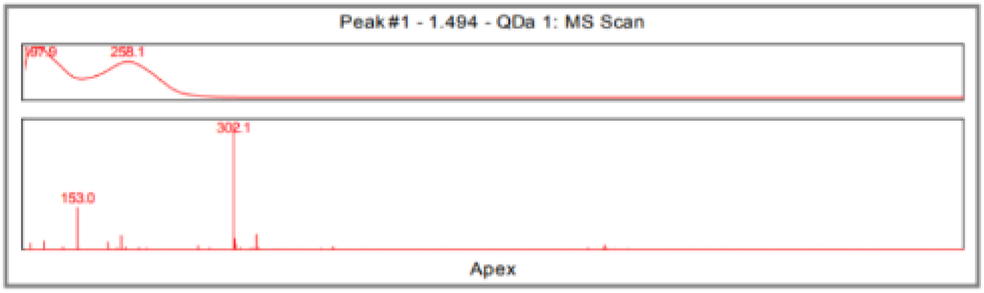
Peak #1 - 1.494 - QDa 1: MS Scan
258.1
302.1
153.0
Apex

3.10 Compound 63

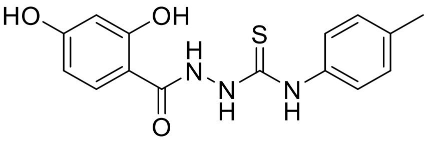

3.10.1 ^{1}H-NMR

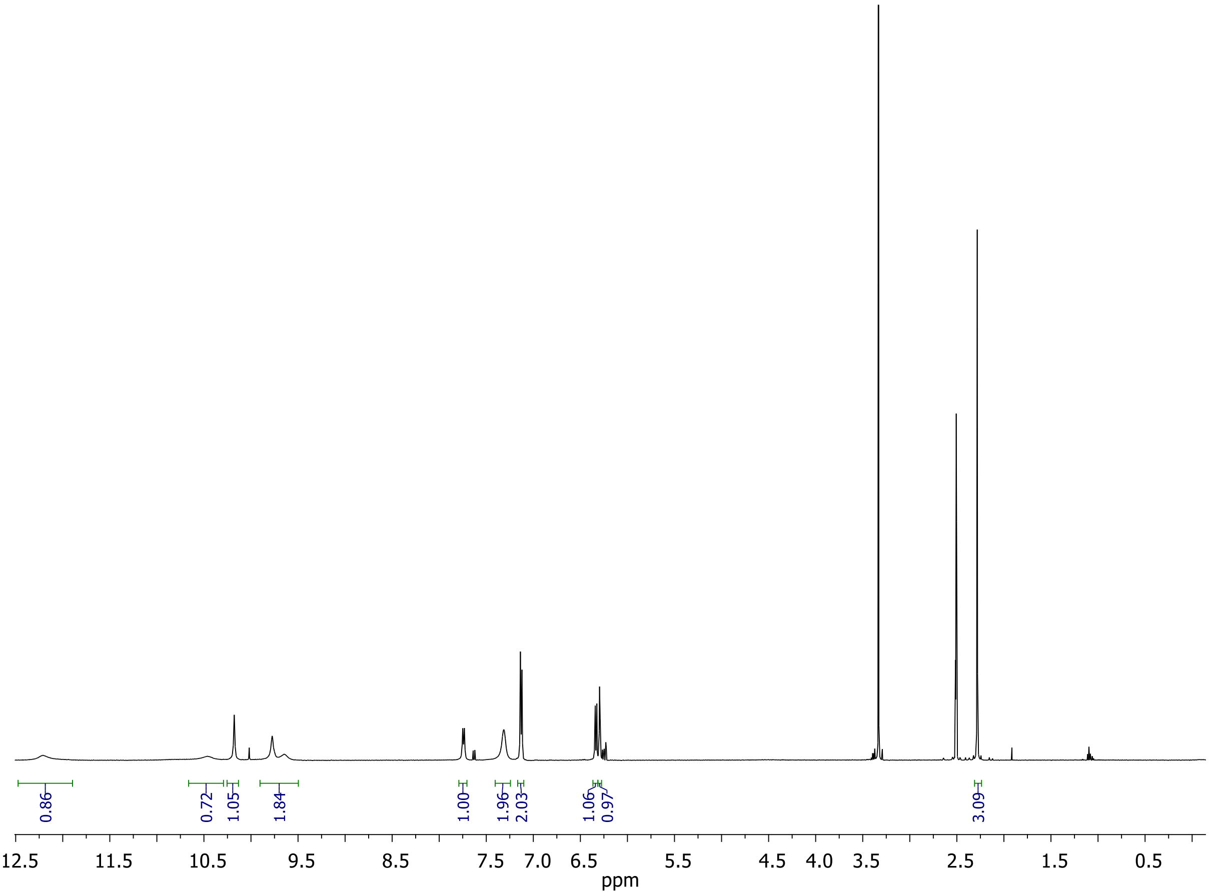

3.10.2 ^{13}C-NMR

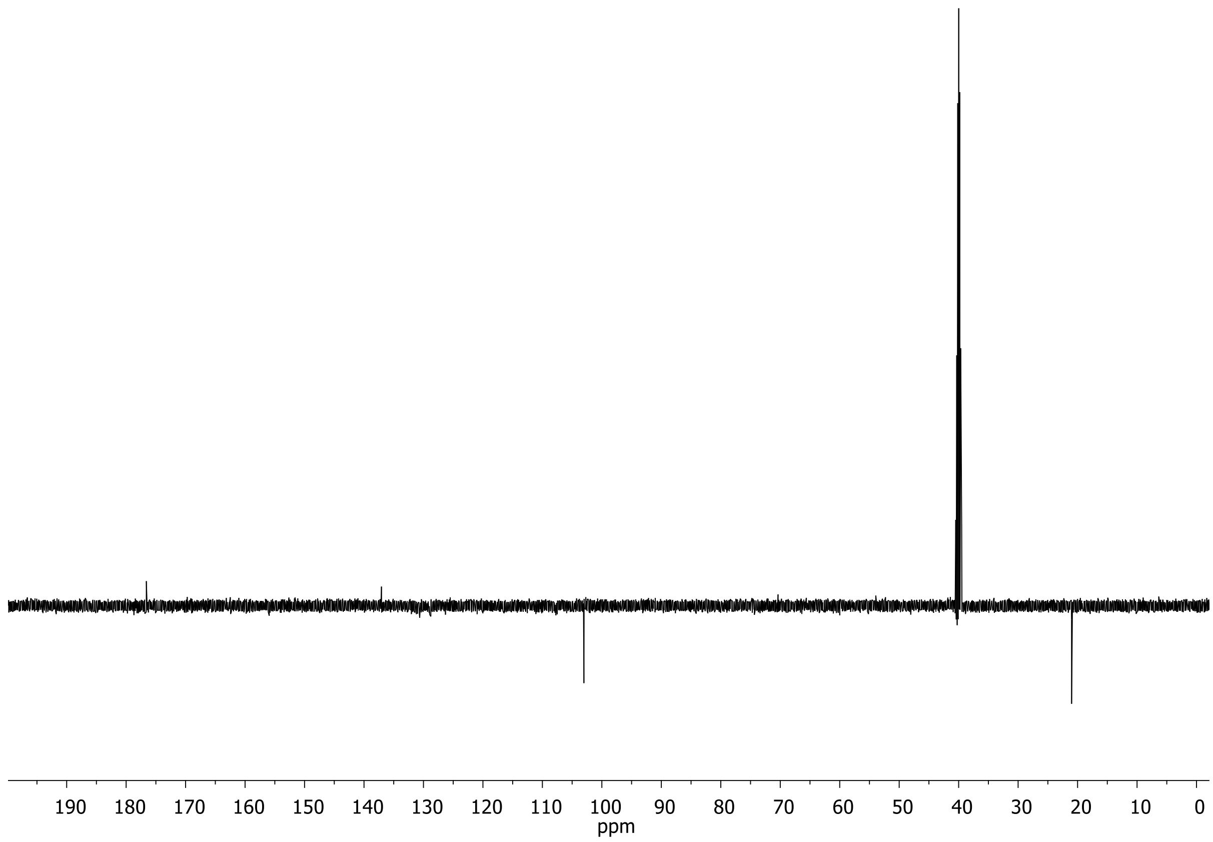
190 180 170 160 150 140 130 120 110 100 90 80 70 60 50 40 30 20 10 0
ppm

3.10.3 UPLC

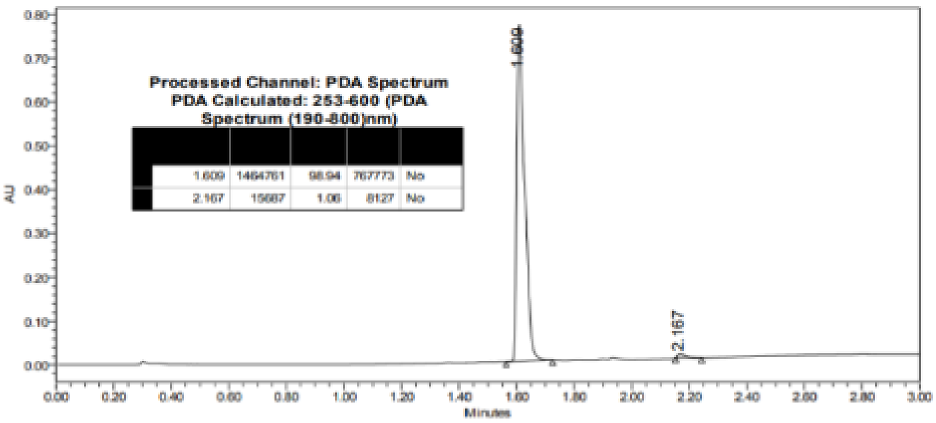
Processed Channel: PDA Spectrum
PDA Calculated: 253-600 (PDA
Spectrum (190-800)nm)
1.609 1464761 98.94 767773 No
2.167 15687 1.06 8127 No
1.609
2.167
AU
Minutes

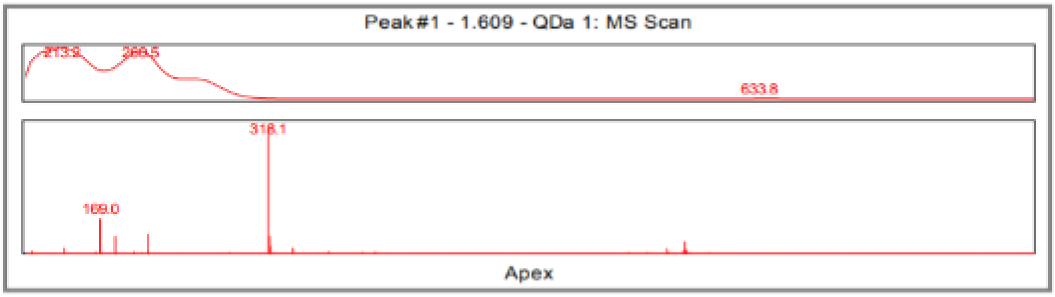
Peak #1 - 1.609 - QDa 1: MS Scan
213.2
266.5
633.8
318.1
169.0
Apex

3.11 Compound 64

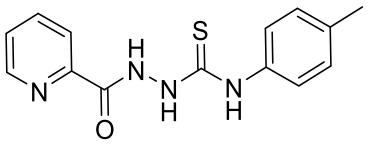

3.11.1 ^{1}H-NMR

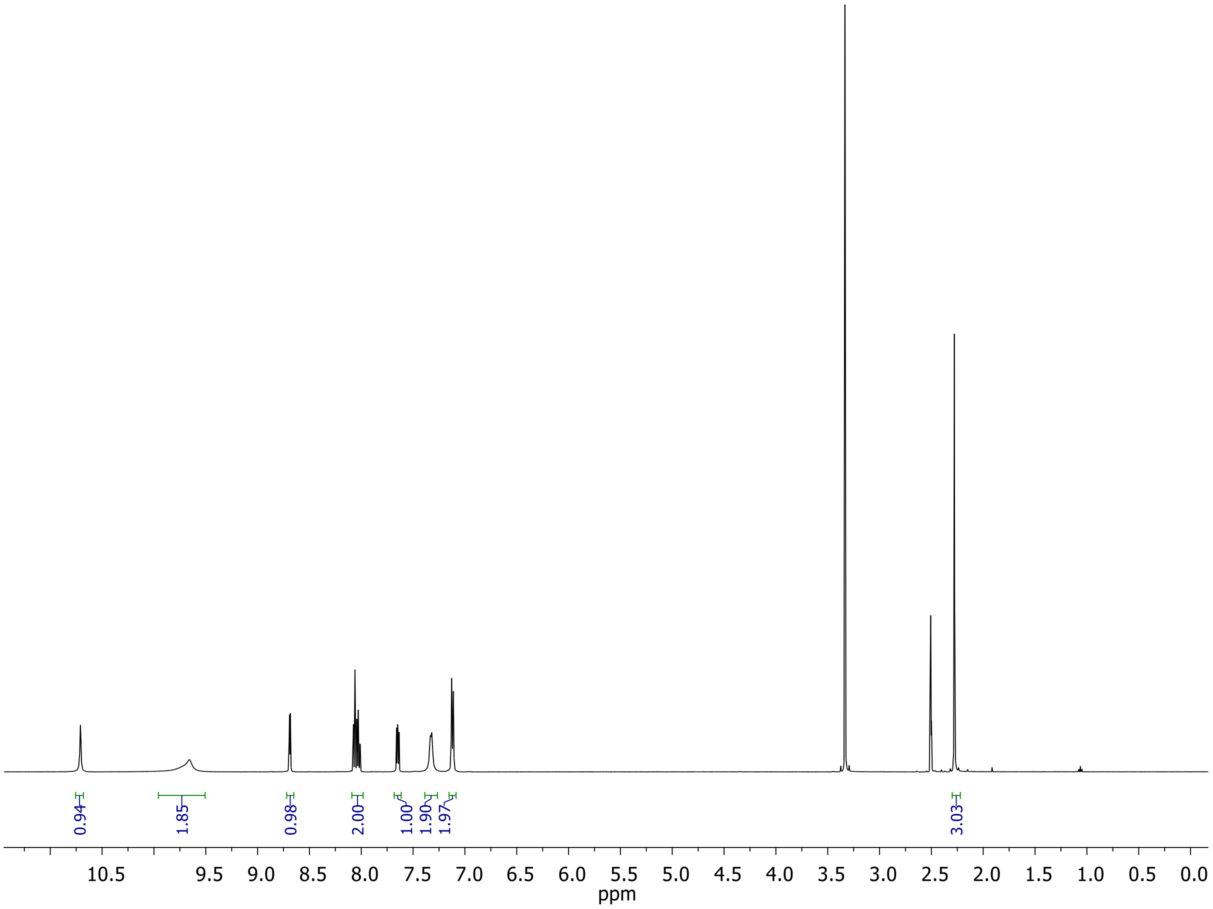

3.11.2 ^{13}C-NMR

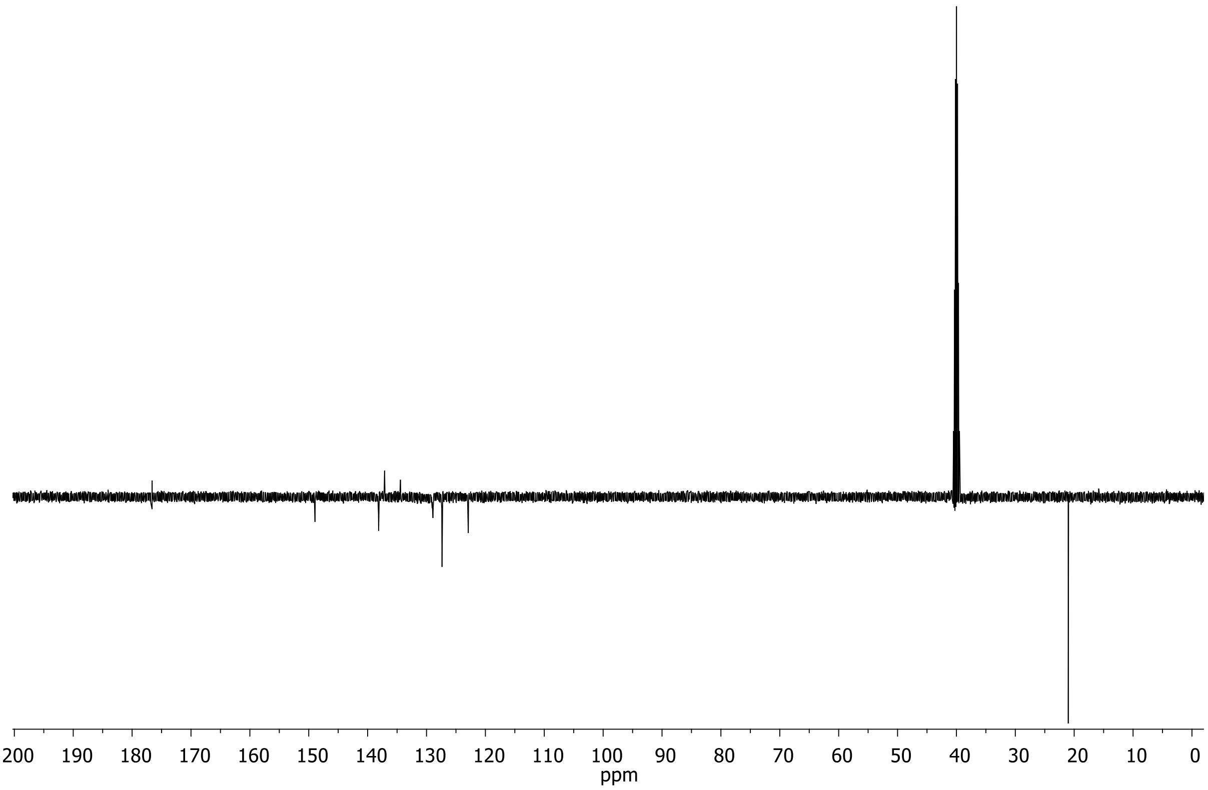

200
190
180
170
160
150
140
130
120
110
100
90
80
70
60
50
40
30
20
10
0
ppm

3.11.3 UPLC

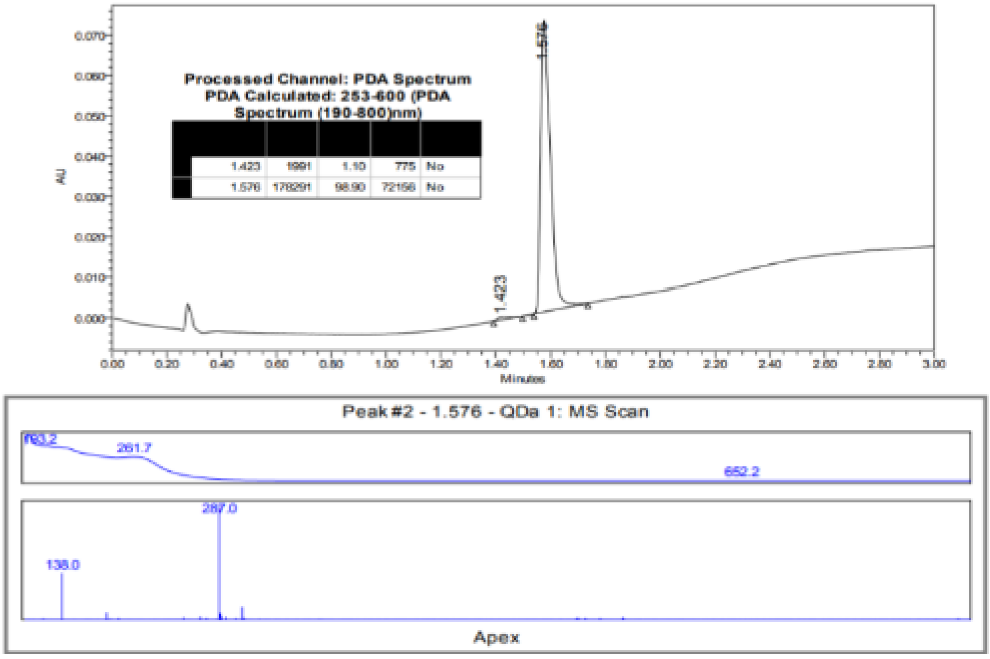
Processed Channel: PDA Spectrum
PDA Calculated: 253-600 (PDA Spectrum (190-800)nm)
1.423 1991 1.10 775 No
1.576 178291 98.90 72156 No
1.576
1.423
AU
Minutes
Peak #2 - 1.576 - QDa 1: MS Scan
261.7
652.2
287.0
138.0
Apex

3.12 Compound 65

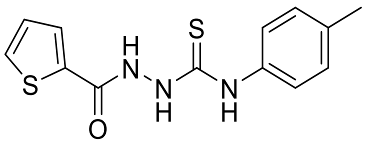

3.12.1 ^{1}H-NMR

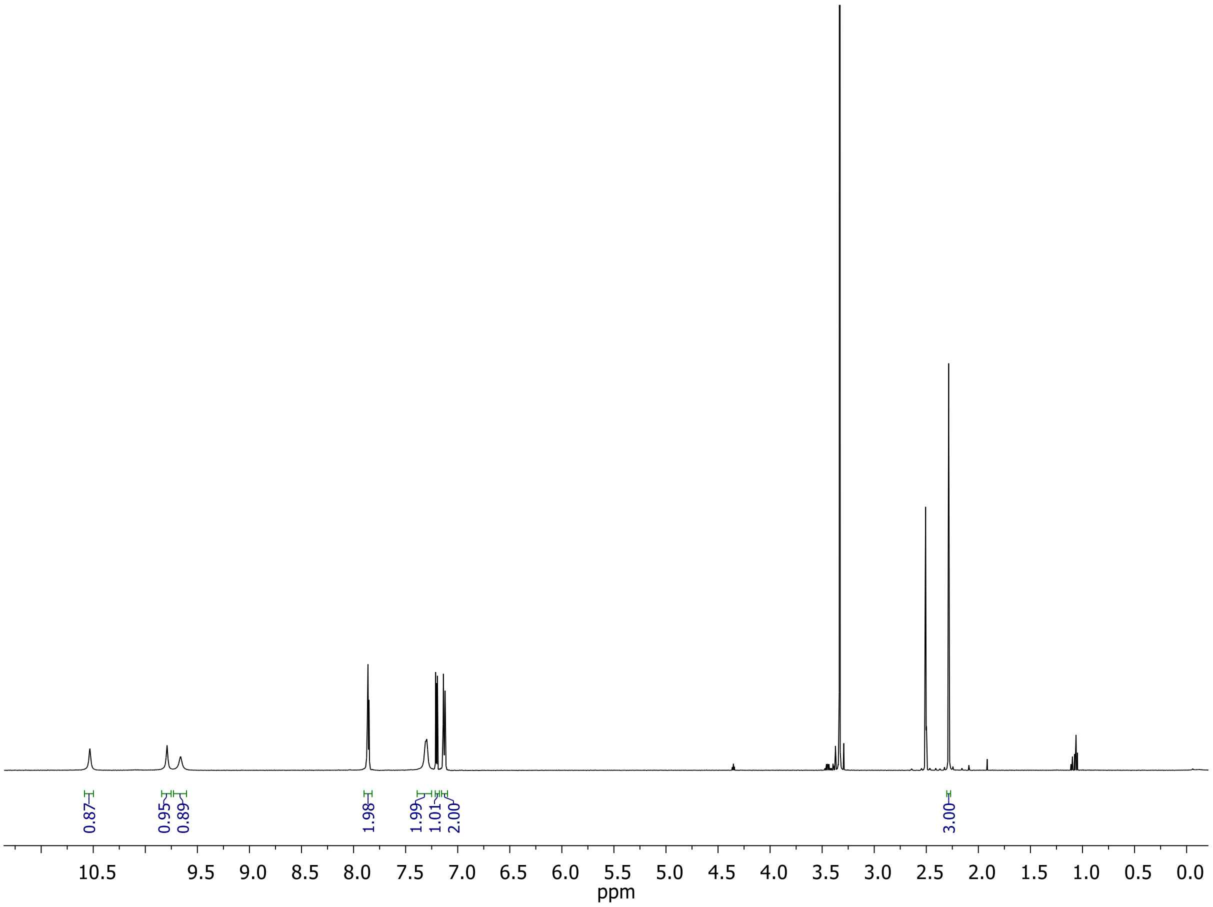

3.12.2 ^{13}C-NMR

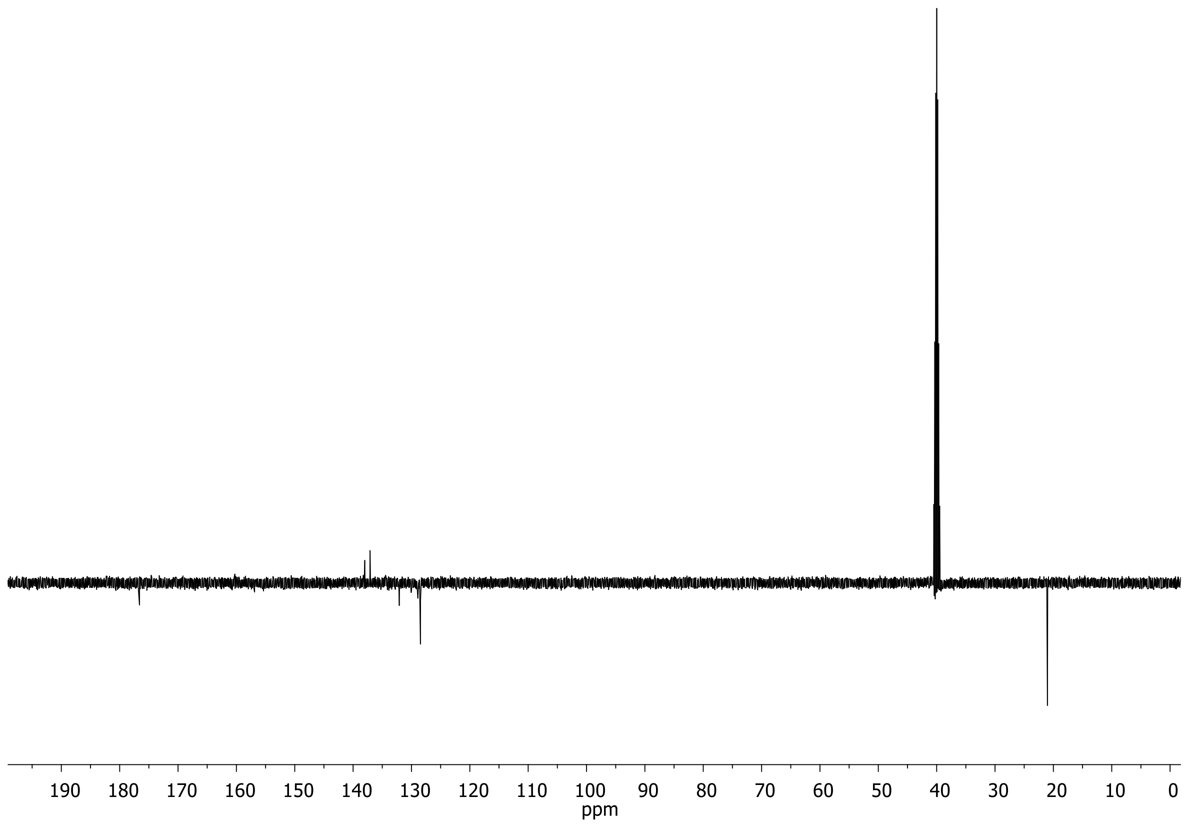
190 180 170 160 150 140 130 120 110 100 90 80 70 60 50 40 30 20 10 0
ppm

3.12.3 UPLC

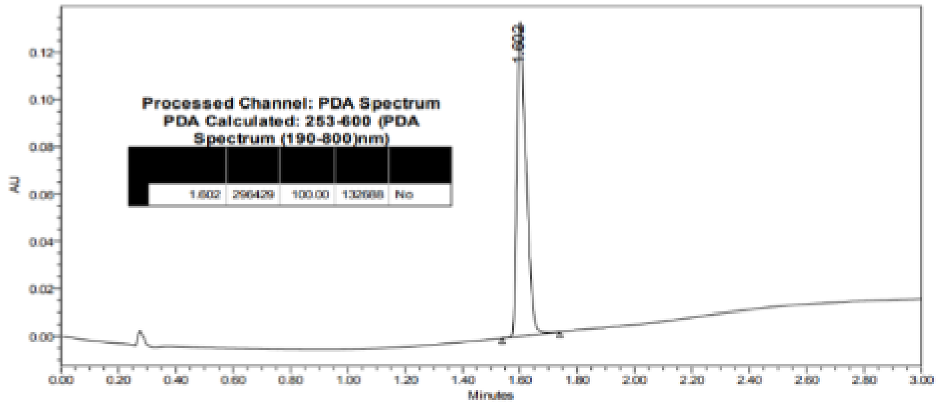
1.602
Processed Channel: PDA Spectrum
PDA Calculated: 253-600 (PDA
Spectrum (190-800)nm)
1.602 296429 100.00 132688 No
AU
0.12
0.10
0.08
0.06
0.04
0.02
0.00
0.00 0.20 0.40 0.60 0.80 1.00 1.20 1.40 1.60 1.80 2.00 2.20 2.40 2.60 2.80 3.00
Minutes

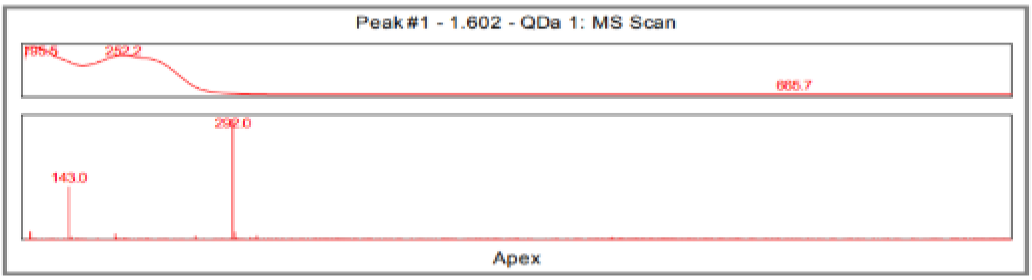
Peak #1 - 1.602 - QDa 1: MS Scan
252.2
665.7
292.0
143.0
Apex

3.13 Compound 66

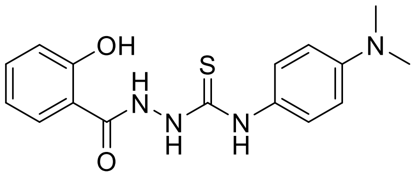

3.13.1 ¹H-NMR

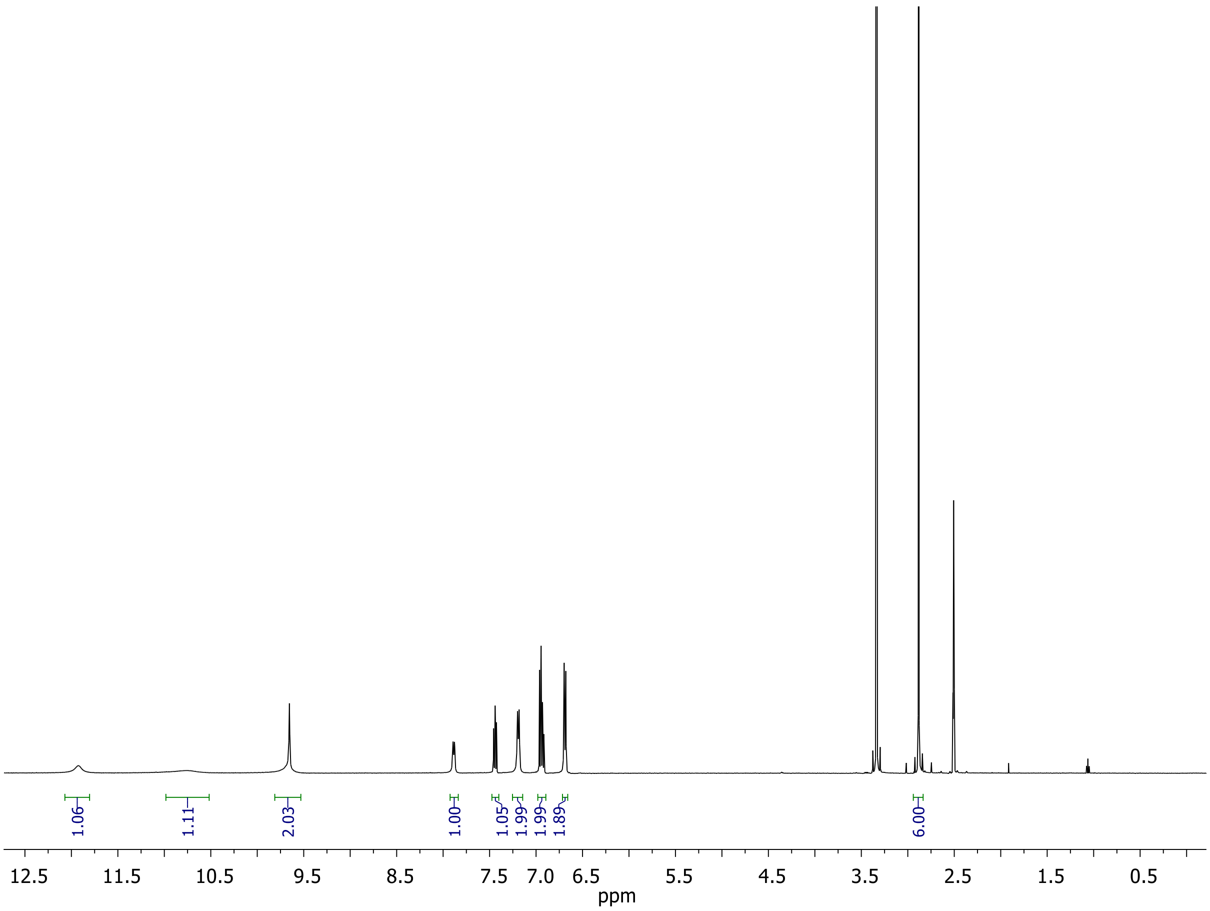

3.13.2 ^{13}C-NMR

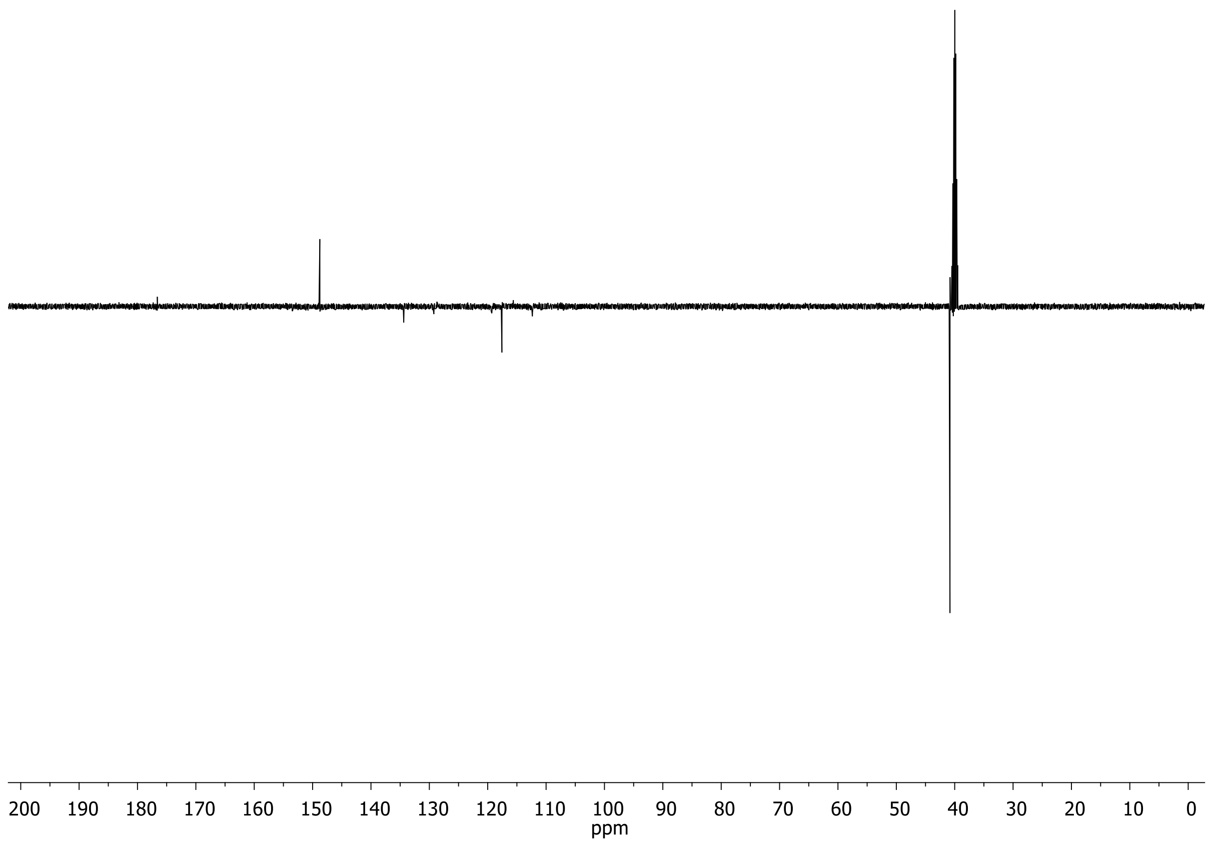

200 190 180 170 160 150 140 130 120 110 100 90 80 70 60 50 40 30 20 10 0
ppm

3.13.3 UPLC

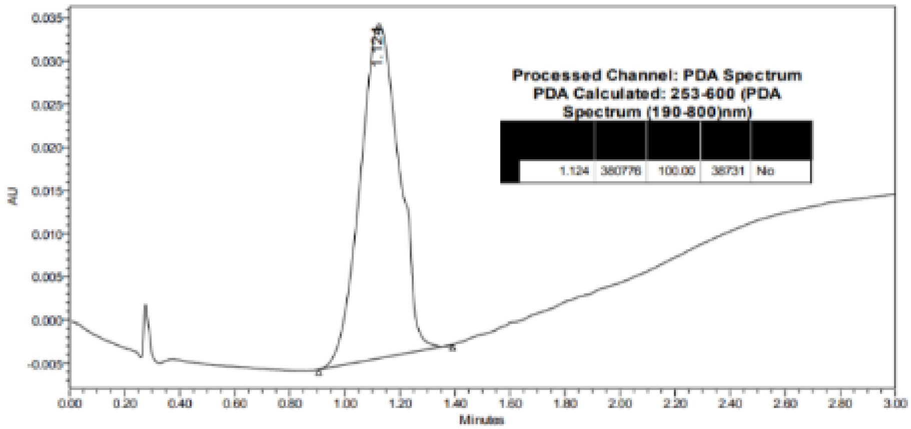

Processed Channel: PDA Spectrum
PDA Calculated: 253-600 (PDA Spectrum (190-800)nm)
1.124
AU
Minutes

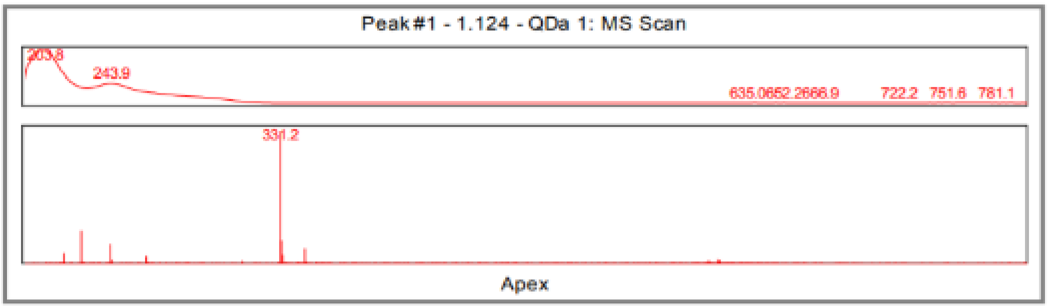

Peak #1 - 1.124 - QDa 1: MS Scan
203.8
243.9
635.0652.2666.9
722.2
751.6
781.1
331.2
Apex

3.14 Compound 67

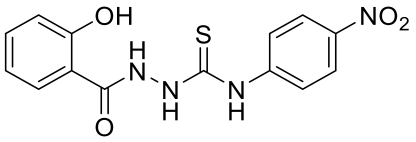

3.14.1 ^{1}H-NMR

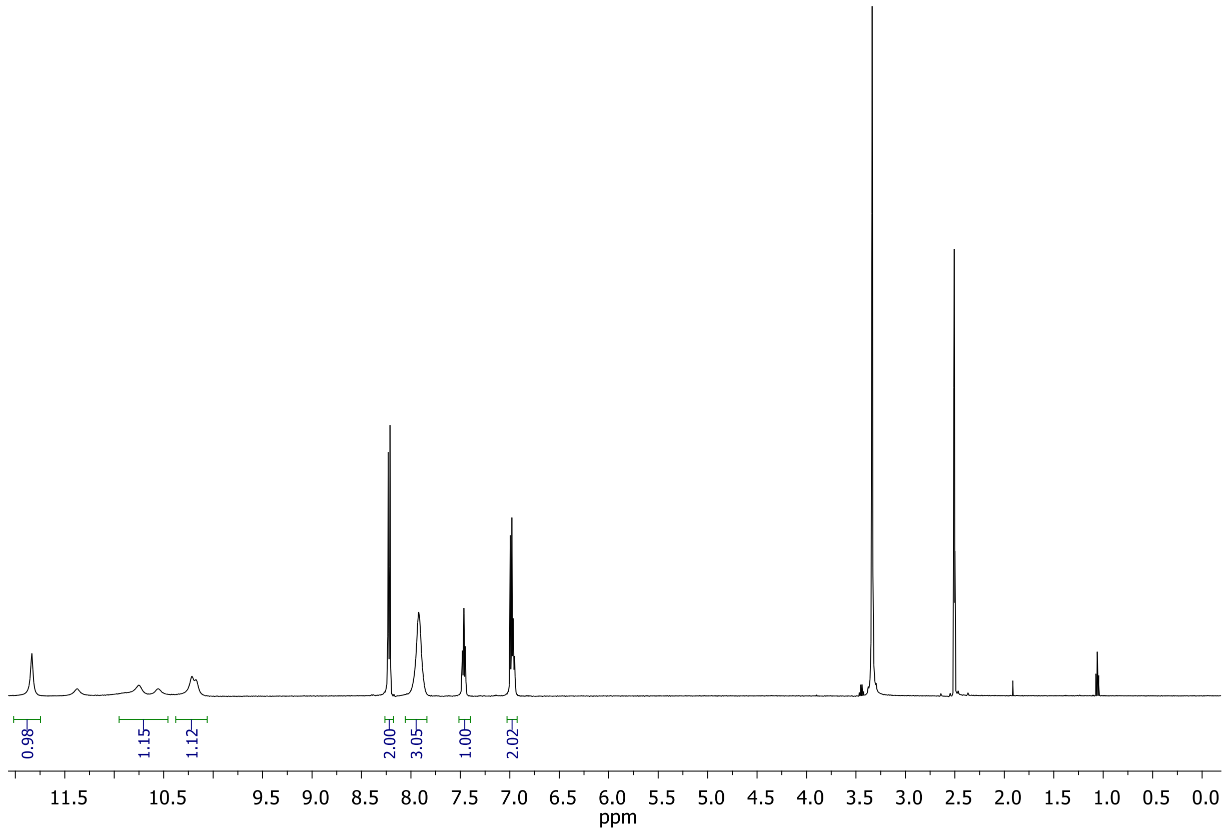

3.14.2 ^{13}C-NMR

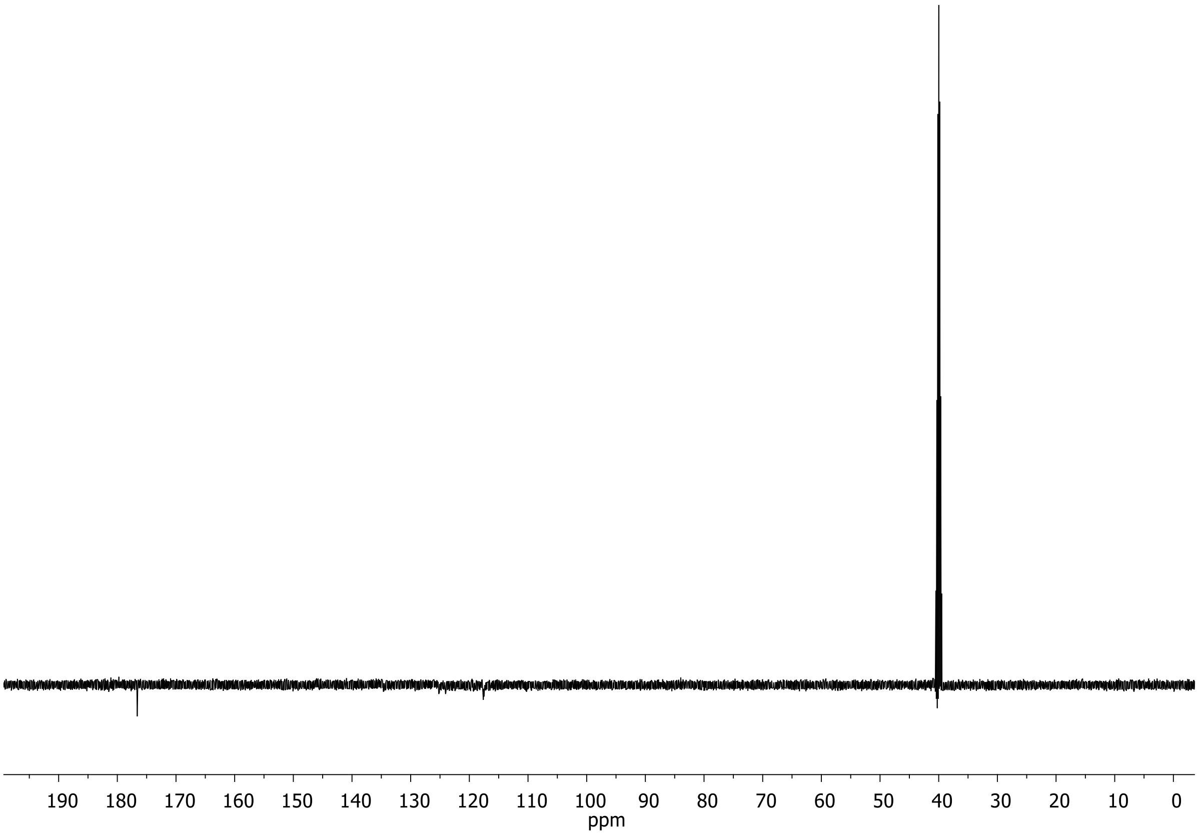
190 180 170 160 150 140 130 120 110 100 90 80 70 60 50 40 30 20 10 0
ppm

3.14.3 UPLC

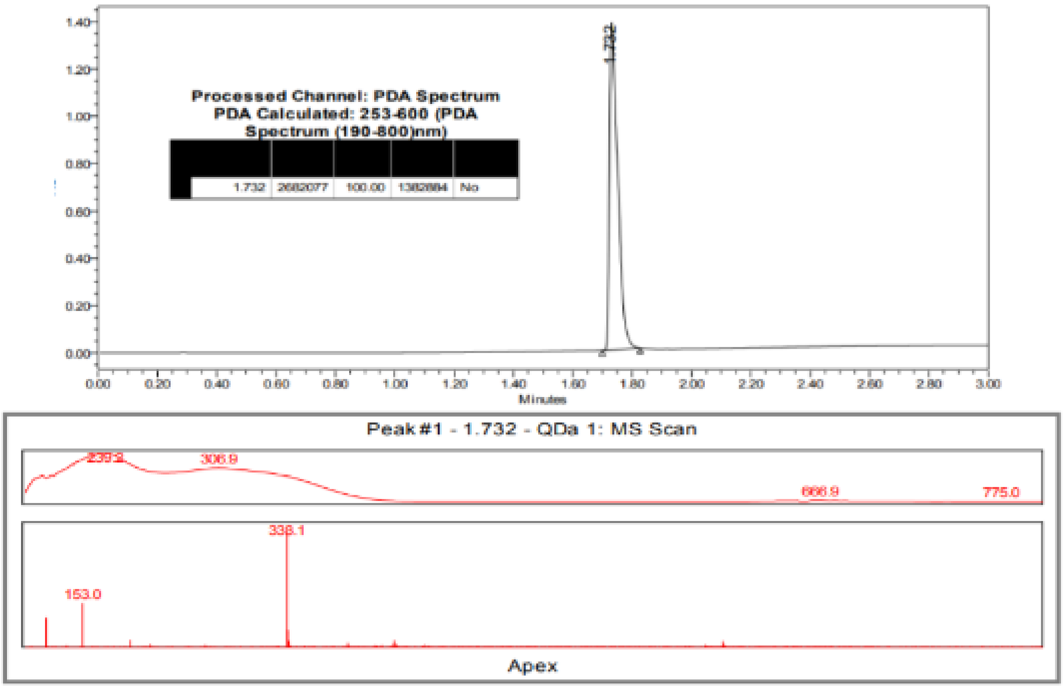
Processed Channel: PDA Spectrum
PDA Calculated: 253-600 (PDA Spectrum (190-800)nm)
1.732
Minutes
Peak #1 - 1.732 - QDa 1: MS Scan
306.9
666.9
775.0
338.1
153.0
Apex

3.15 Compound 68

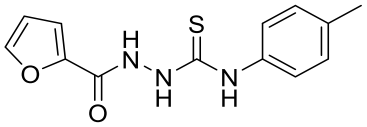

3.15.1 ^{1}H-NMR

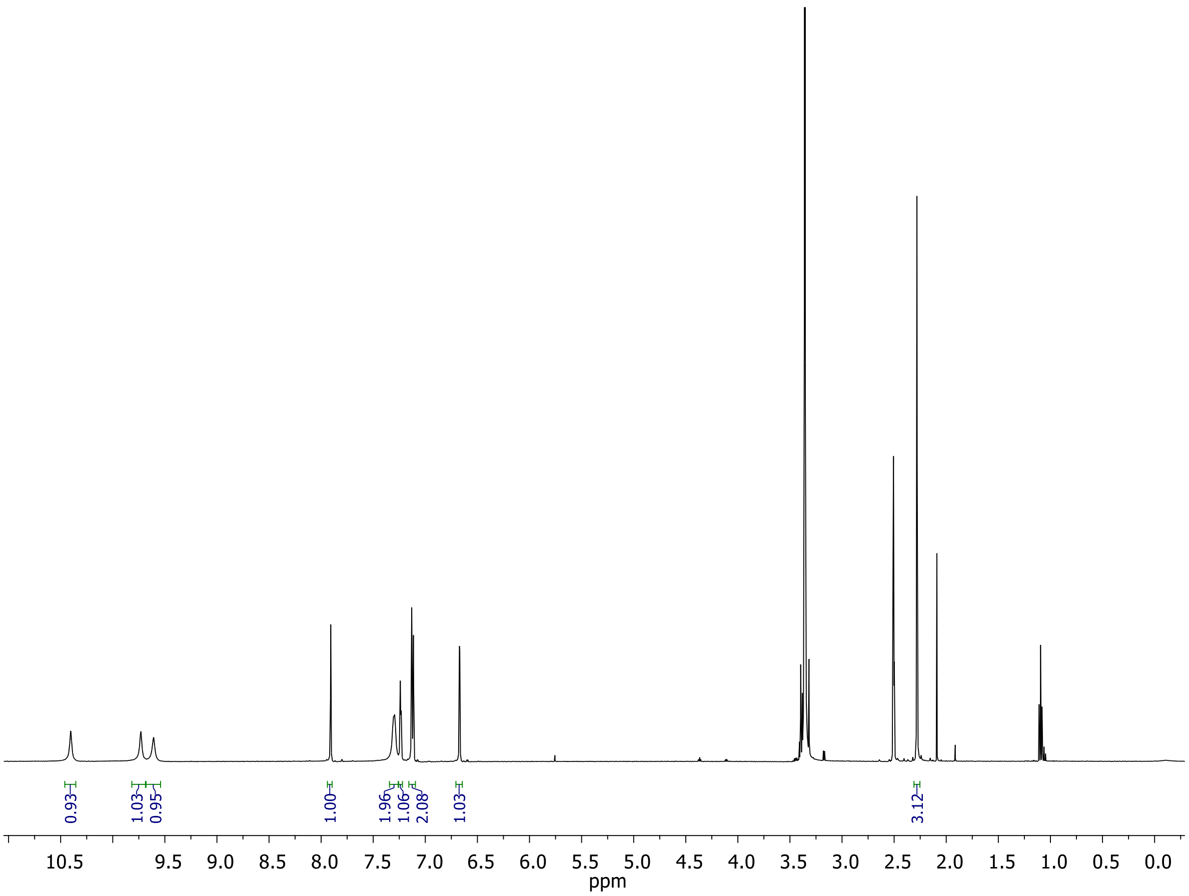

3.15.2 ^{13}C-NMR

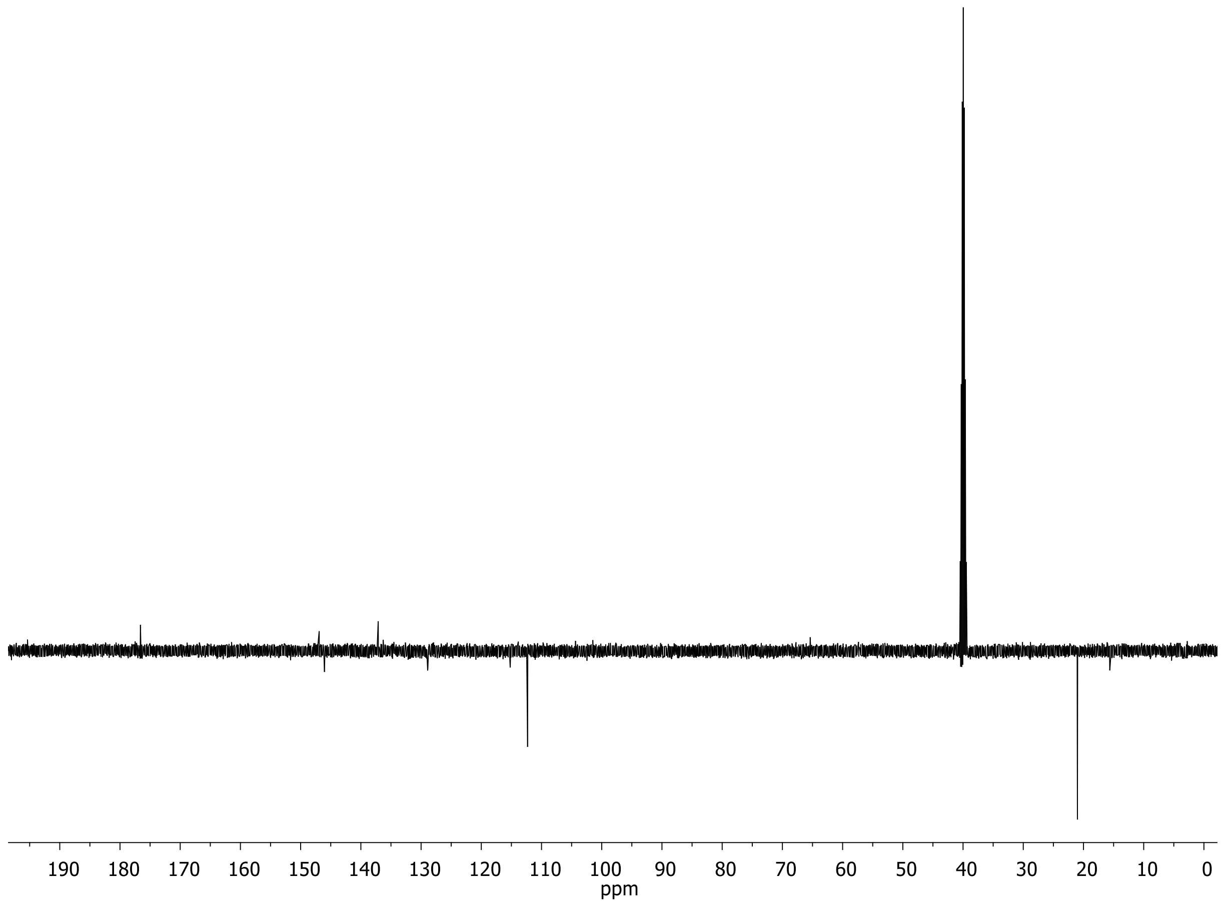

190 180 170 160 150 140 130 120 110 100 90 80 70 60 50 40 30 20 10 0
ppm

3.15.3 UPLC

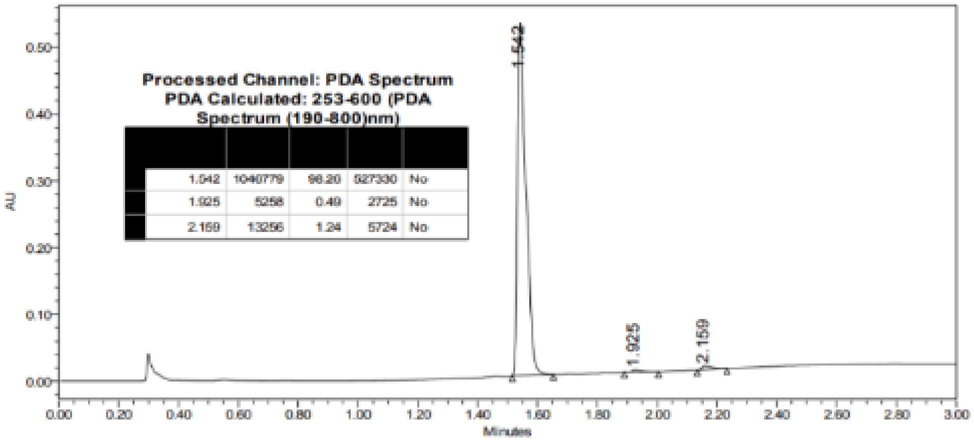
Processed Channel: PDA Spectrum
PDA Calculated: 253-600 (PDA Spectrum (190-800)nm)
1.542 1040779 98.20 527330 No
1.925 5258 0.49 2725 No
2.159 13256 1.24 5724 No
1.542
1.925
2.159
AU
Minutes

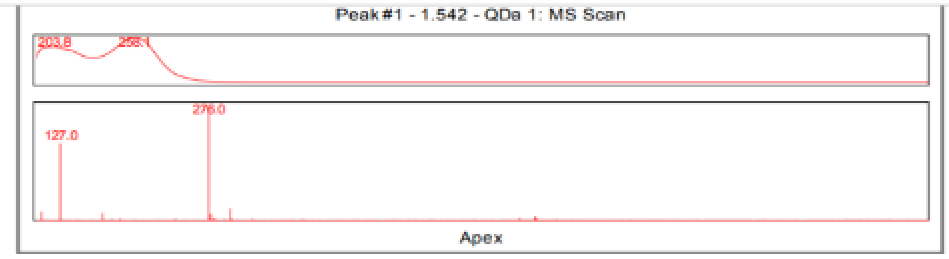
Peak #1 - 1.542 - QDa 1: MS Scan
203.8
258.1
276.0
127.0
Apex

3.16 Compound 69

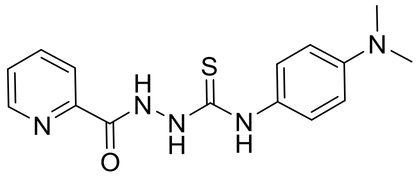

3.16.1 ^{1}H-NMR

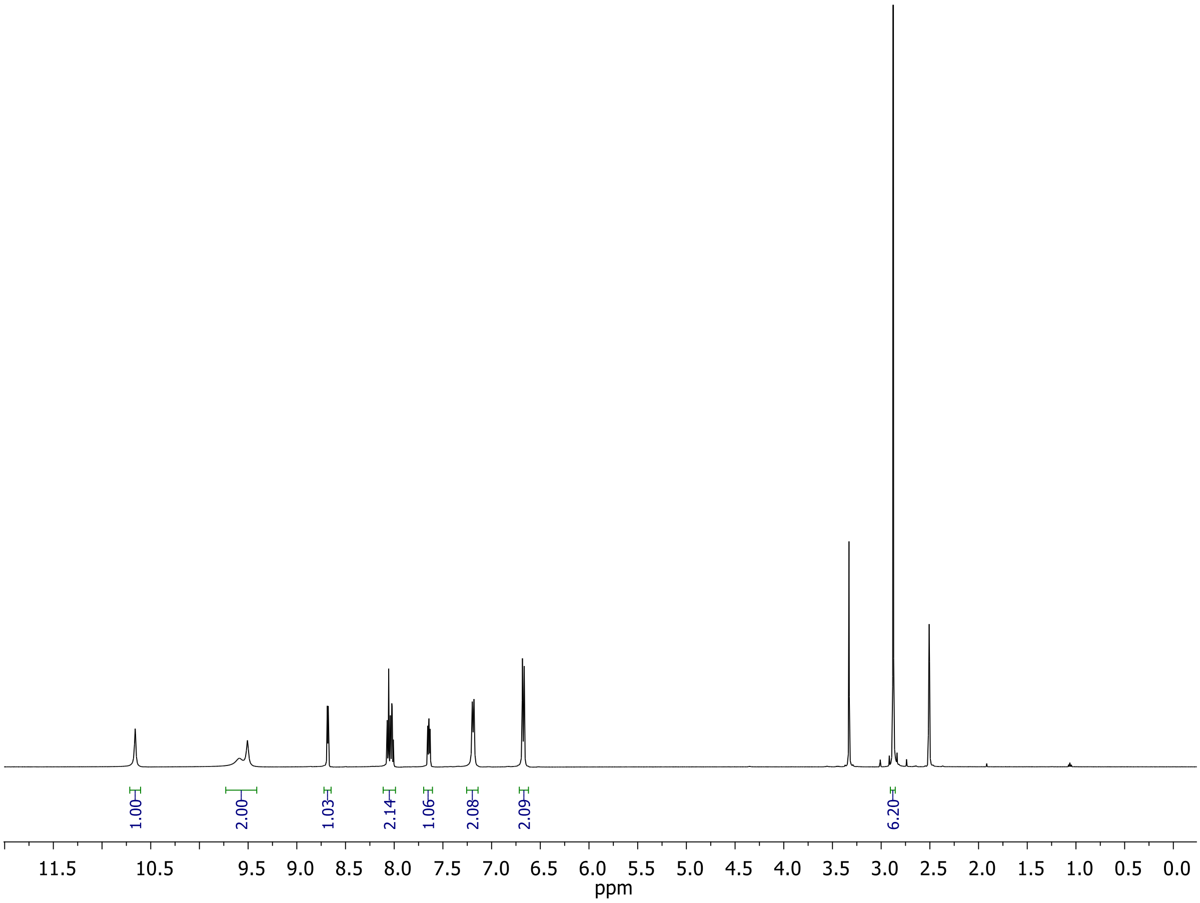

3.16.2 ^{13}C-NMR

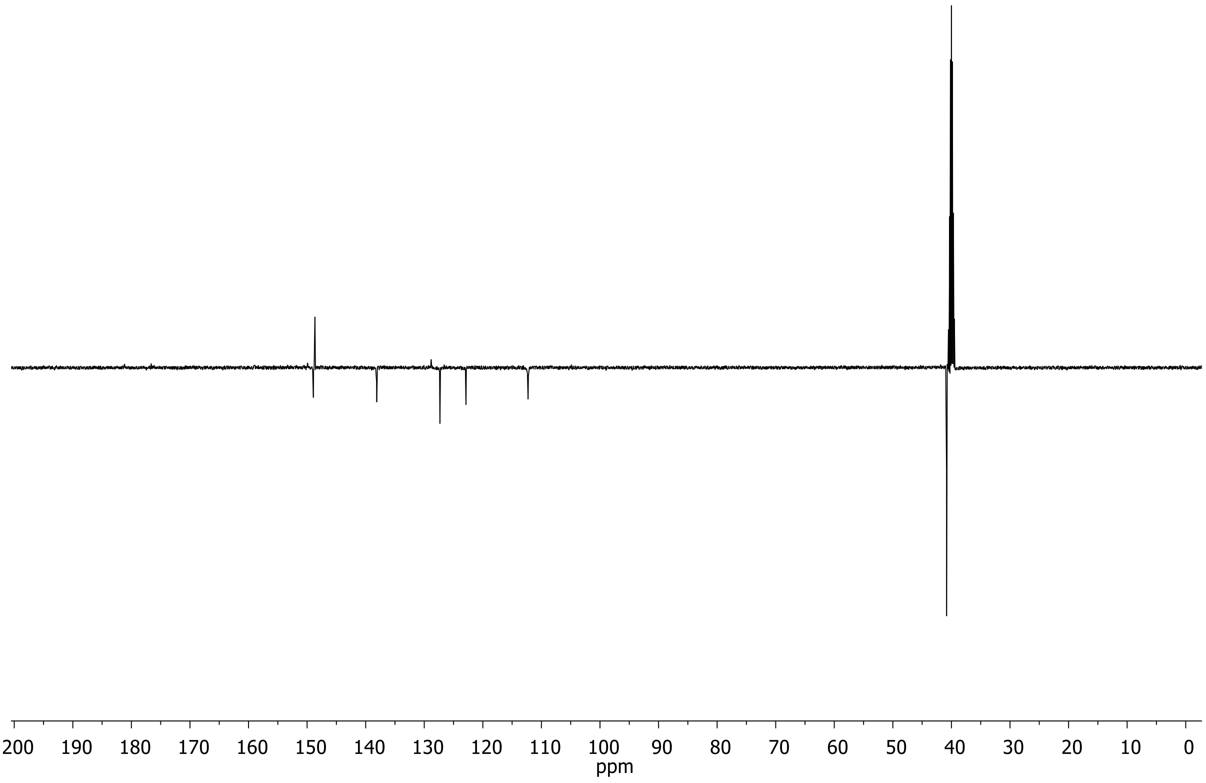
200 190 180 170 160 150 140 130 120 110 100 90 80 70 60 50 40 30 20 10 0
ppm

3.16.3 UPLC

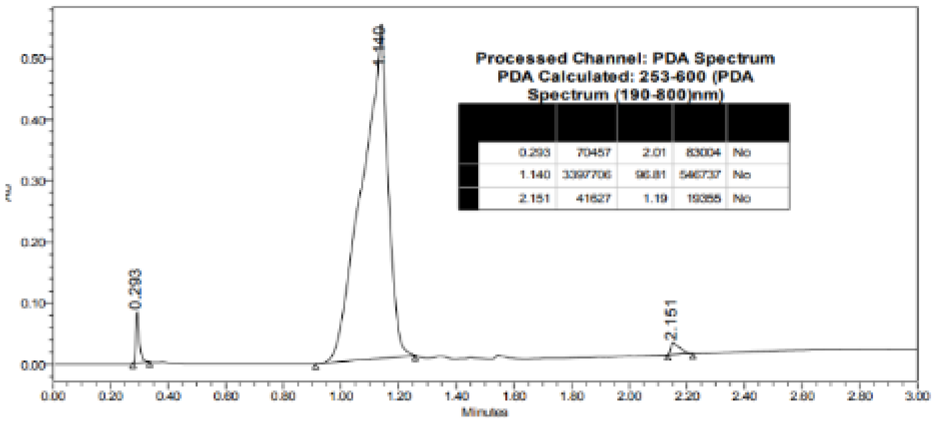

Processed Channel: PDA Spectrum
PDA Calculated: 253-600 (PDA Spectrum (190-800)nm)
0.293 70457 2.01 83004 No
1.140 3397706 96.81 546737 No
2.151 41627 1.19 19355 No
1.140
0.293
2.151
Minutes

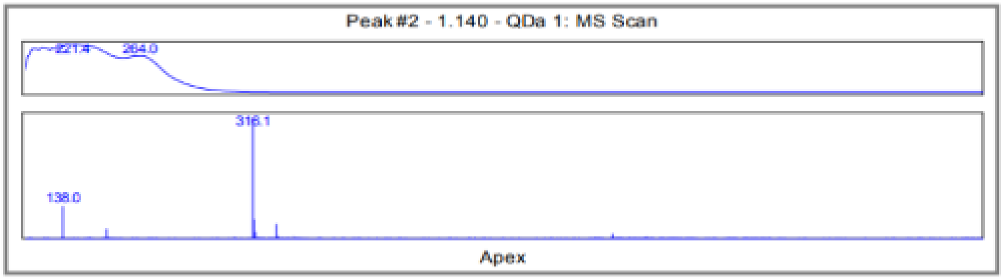

Peak #2 - 1.140 - QDa 1: MS Scan
221.4
264.0
316.1
138.0
Apex

3.17 Compound 70

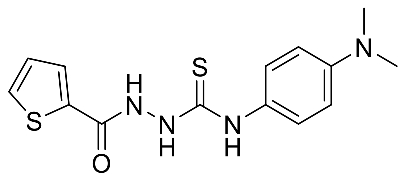

3.17.1 ^{1}H-NMR

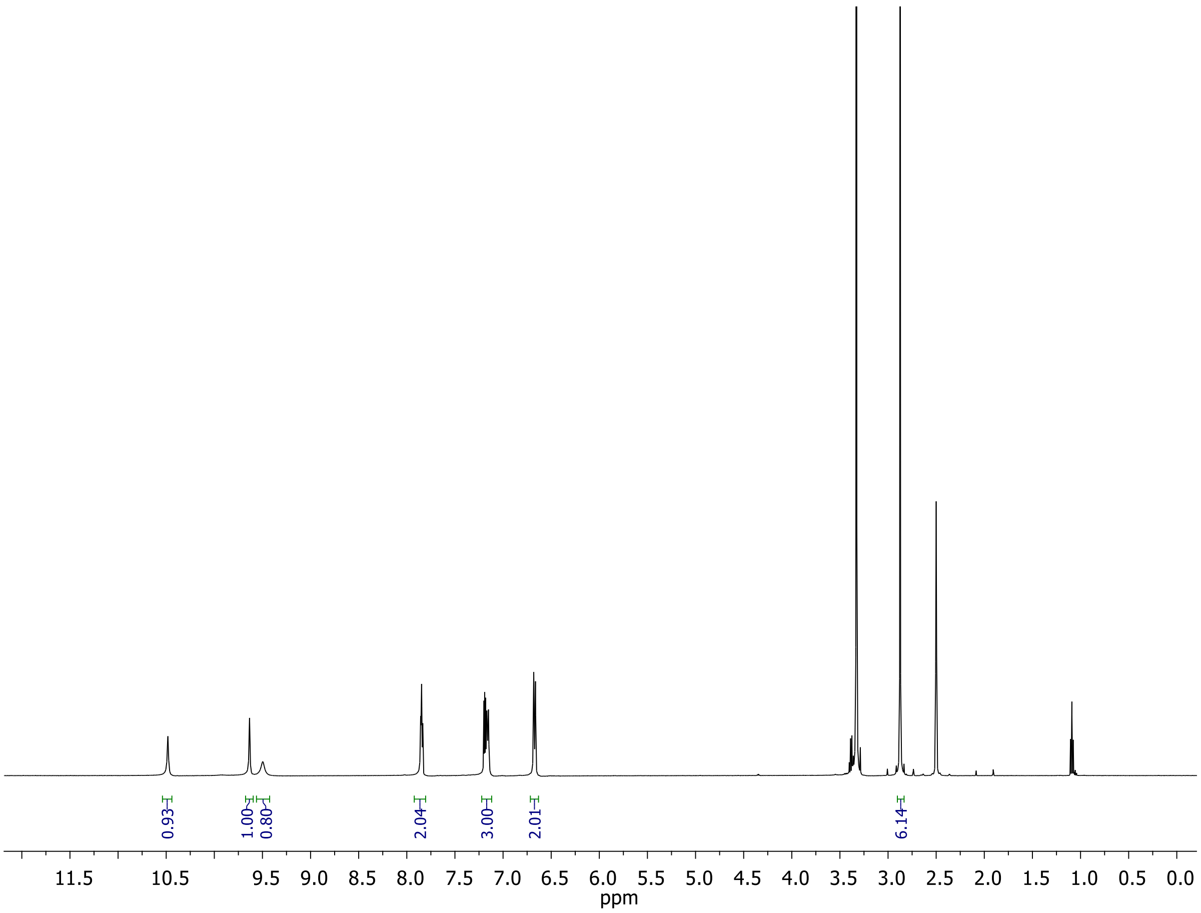

3.17.2 ^{13}C-NMR

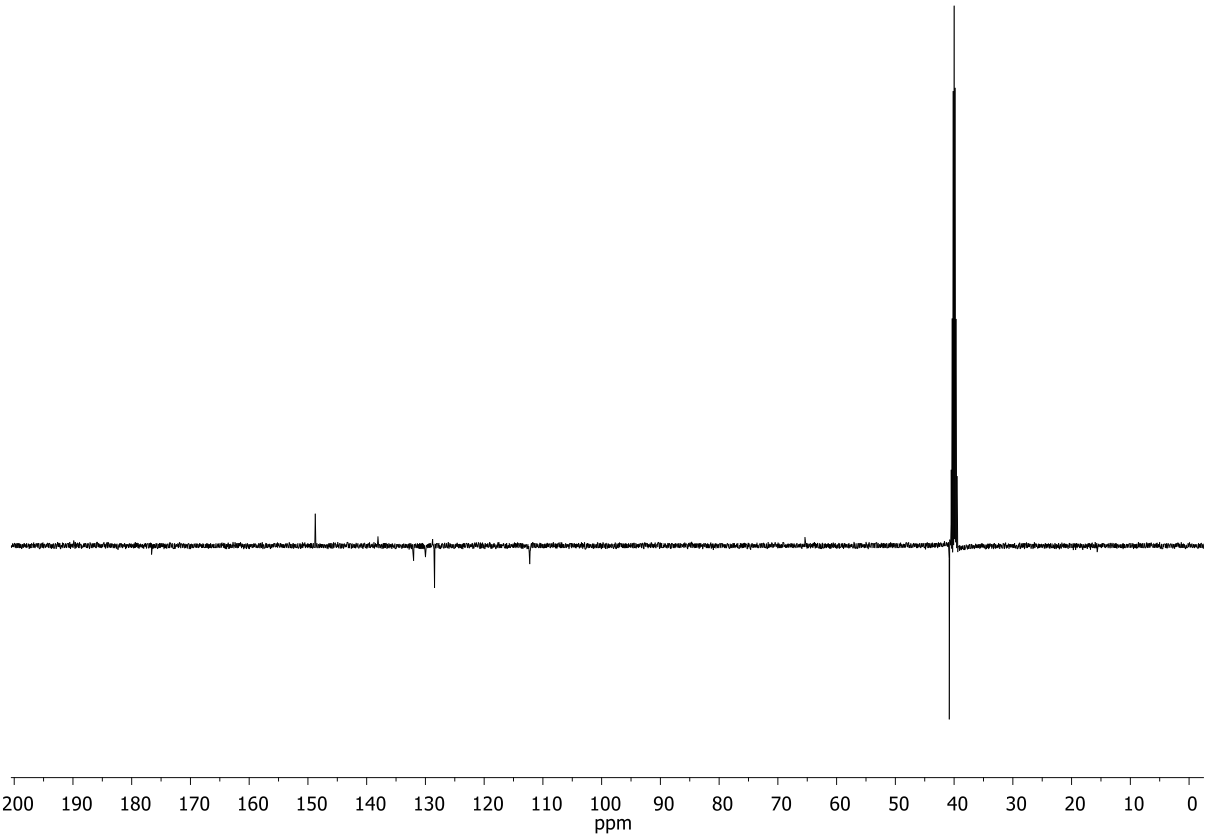
200 190 180 170 160 150 140 130 120 110 100 90 80 70 60 50 40 30 20 10 0
ppm

3.17.3 UPLC

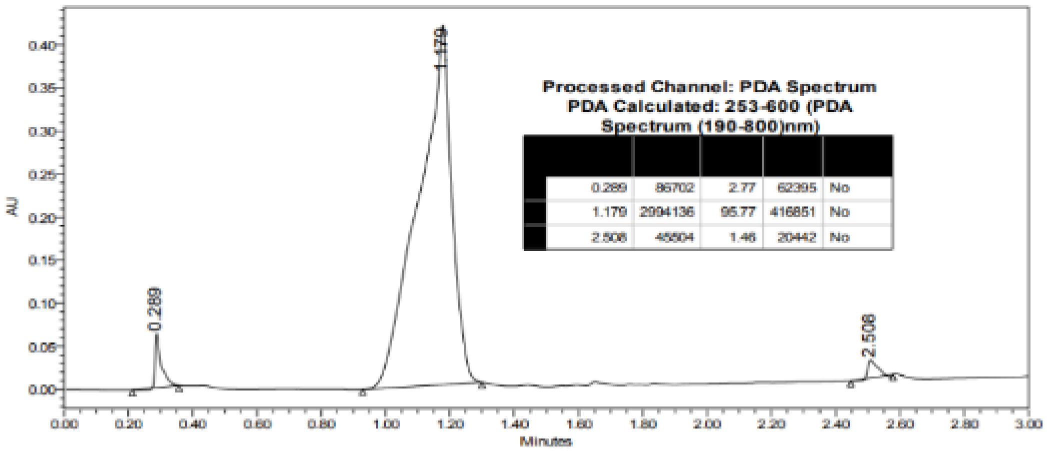

Processed Channel: PDA Spectrum
PDA Calculated: 253-600 (PDA Spectrum (190-800)nm)
0.289 86702 2.77 62395 No
1.179 2994136 95.77 416851 No
2.508 45604 1.46 20442 No
0.289
1.179
2.508
AU
Minutes

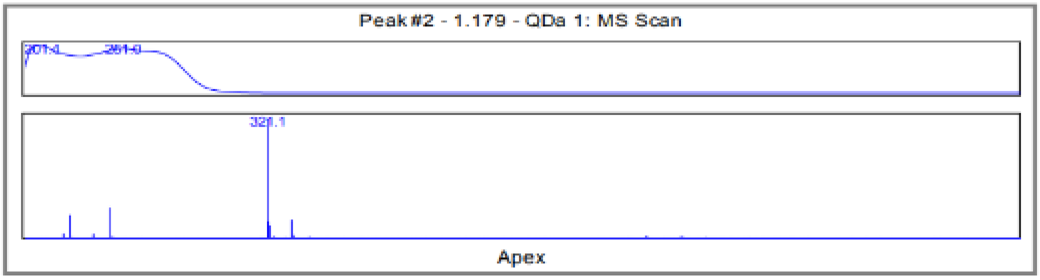

Peak #2 - 1.179 - QDa 1: MS Scan
Apex

3.18 Compound 71

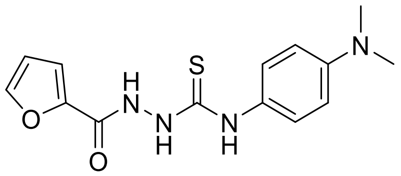

3.18.1 ^{1}H-NMR

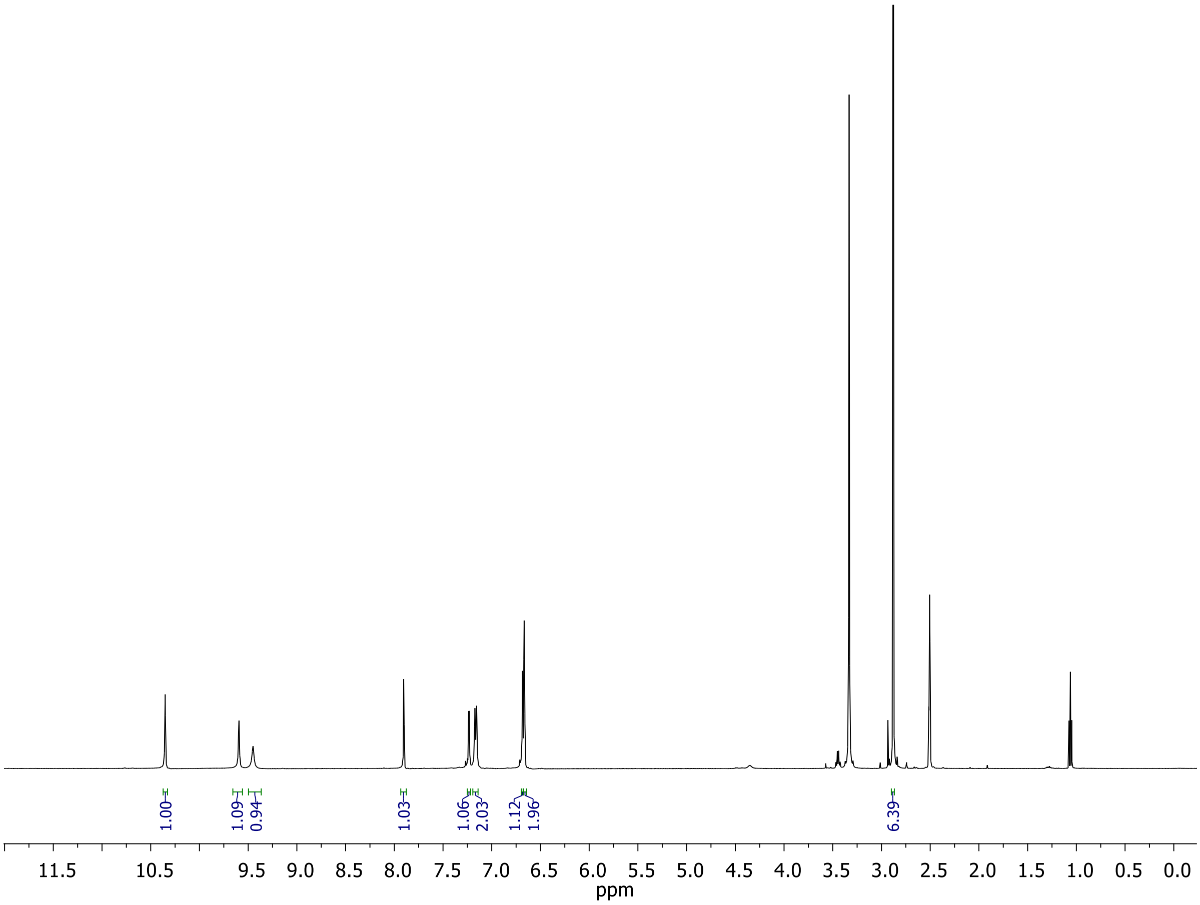

3.18.2 ^{13}C-NMR

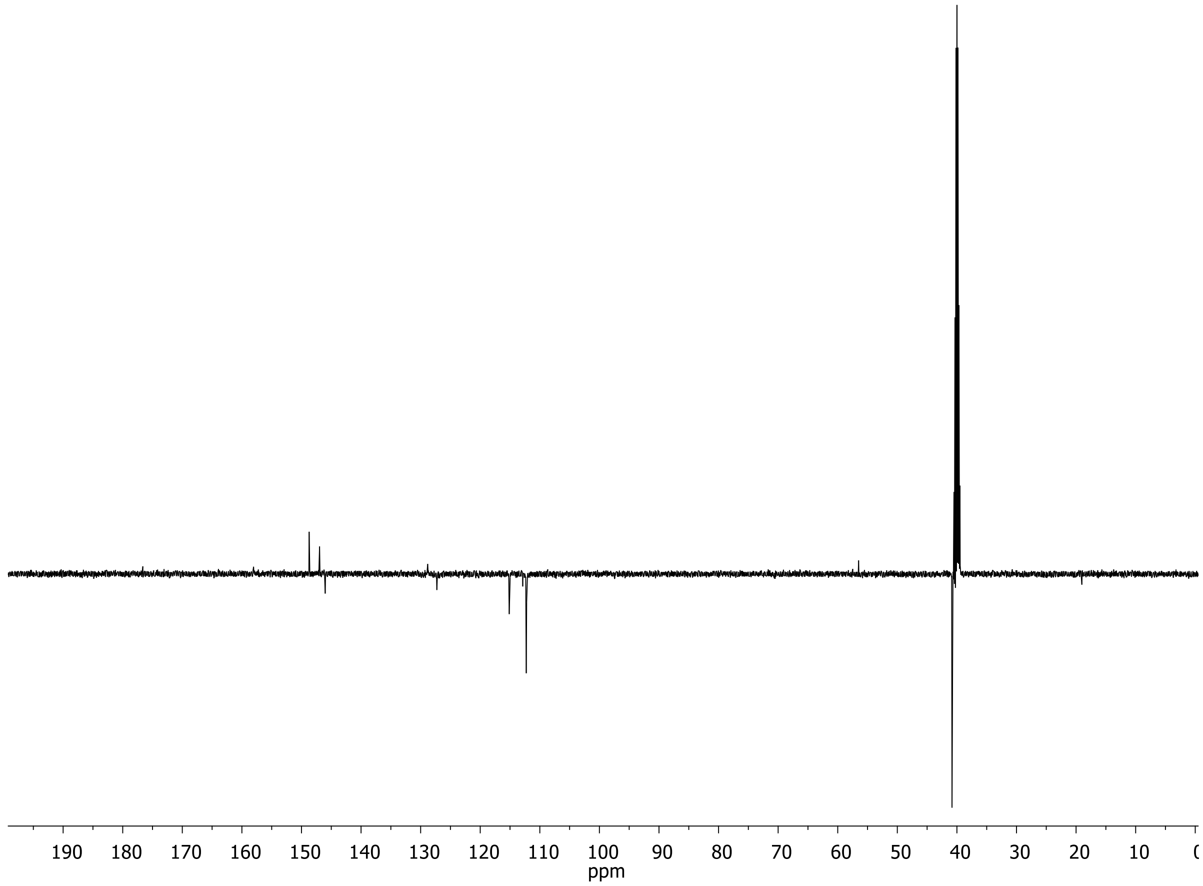
190 180 170 160 150 140 130 120 110 100 90 80 70 60 50 40 30 20 10 0
ppm

3.18.3 UPLC

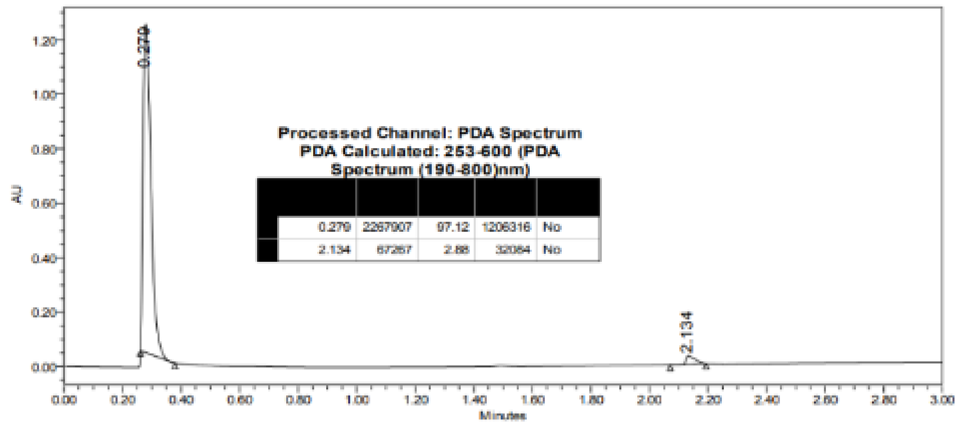
0.279
2.134
Processed Channel: PDA Spectrum
PDA Calculated: 253-600 (PDA
Spectrum (190-800)nm)
0.279 2267907 97.12 1206316 No
2.134 67267 2.88 32064 No
AU
Minutes

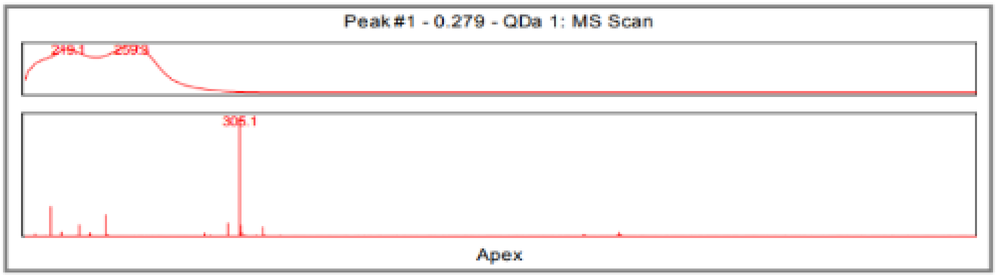
Peak #1 - 0.279 - QDa 1: MS Scan
249.1
259.9
306.1
Apex

3.19 Compound 72

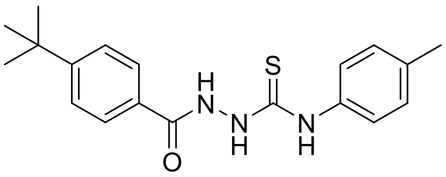

3.19.1 ^{1}H-NMR

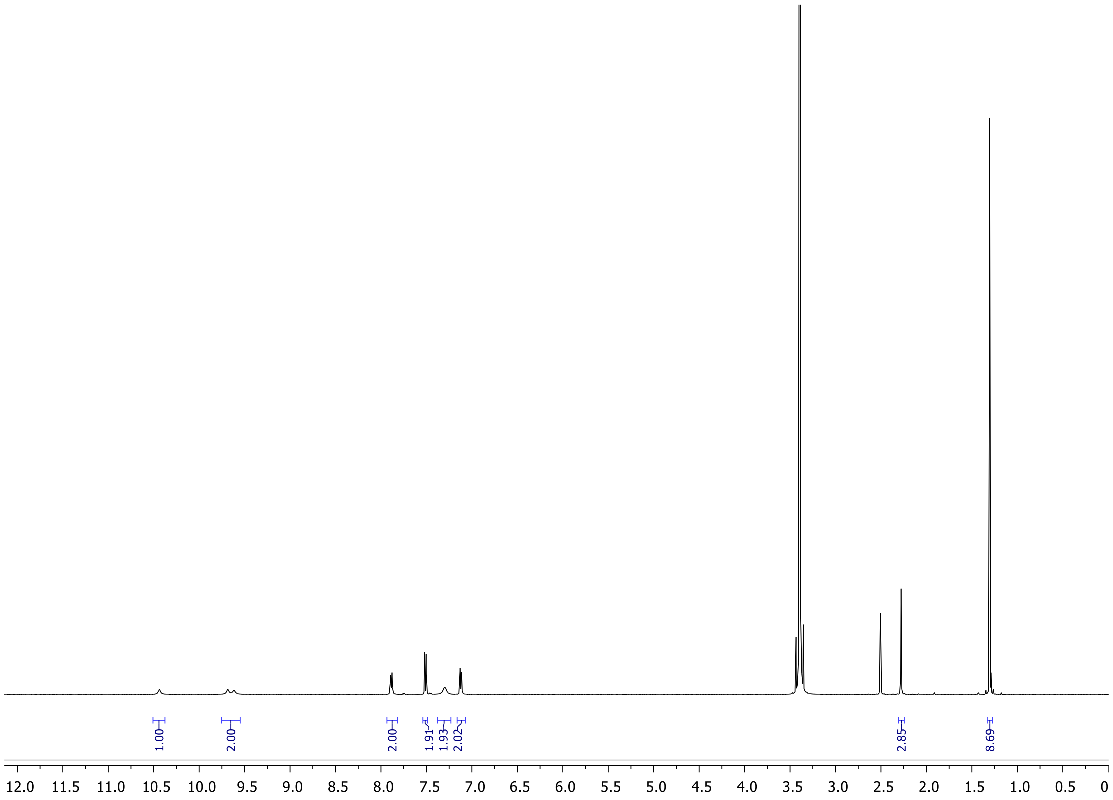

3.19.2 ^{13}C-NMR

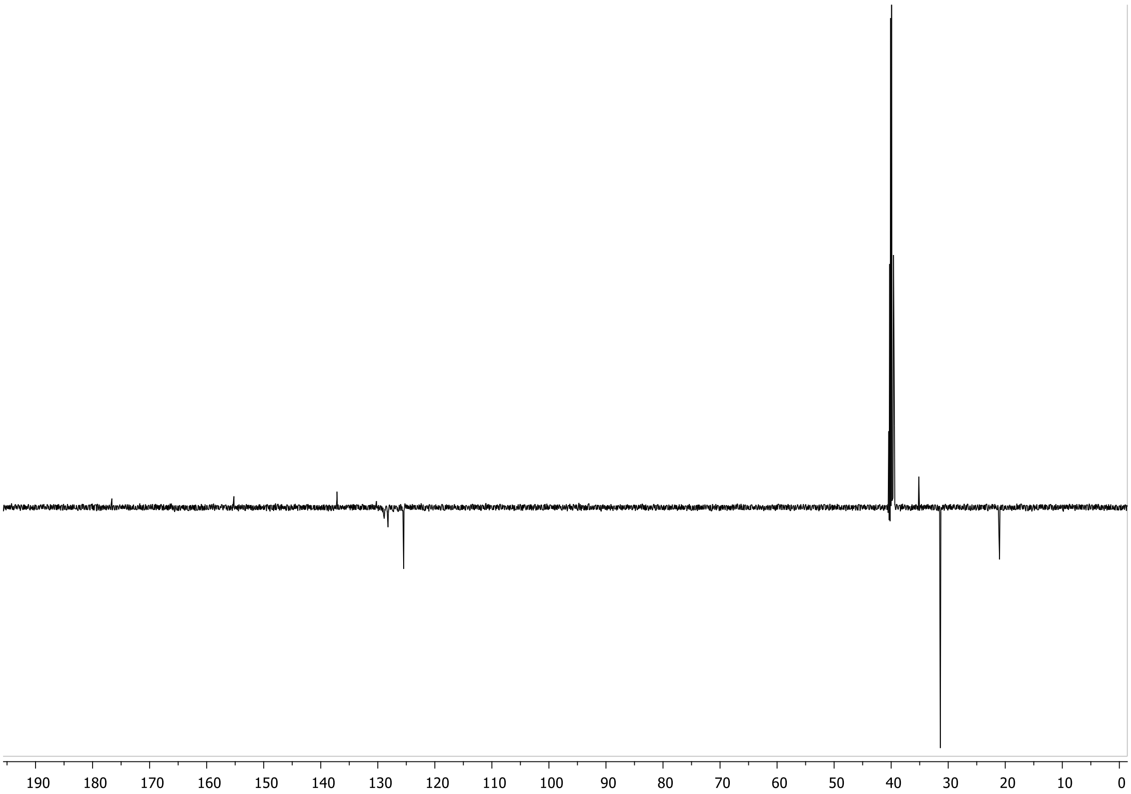

3.19.3 UPLC

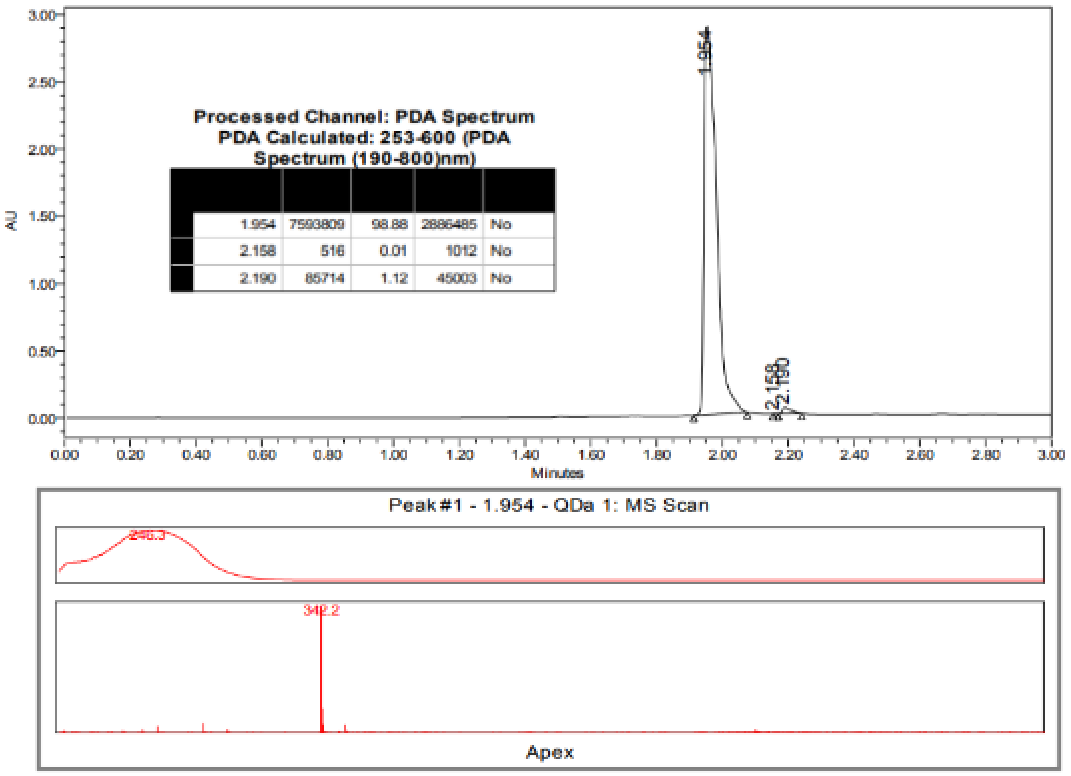

Processed Channel: PDA Spectrum
PDA Calculated: 253-600 (PDA Spectrum (190-800)nm)
1.954 7593809 98.88 2886485 No
2.158 516 0.01 1012 No
2.190 85714 1.12 45003 No
AU
Minutes
1.954
2.158
2.190
Peak #1 - 1.954 - QDa 1: MS Scan
246.3
342.2
Apex

3.20 Compound 73

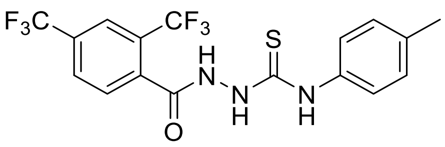

3.20.1 ^{1}H-NMR

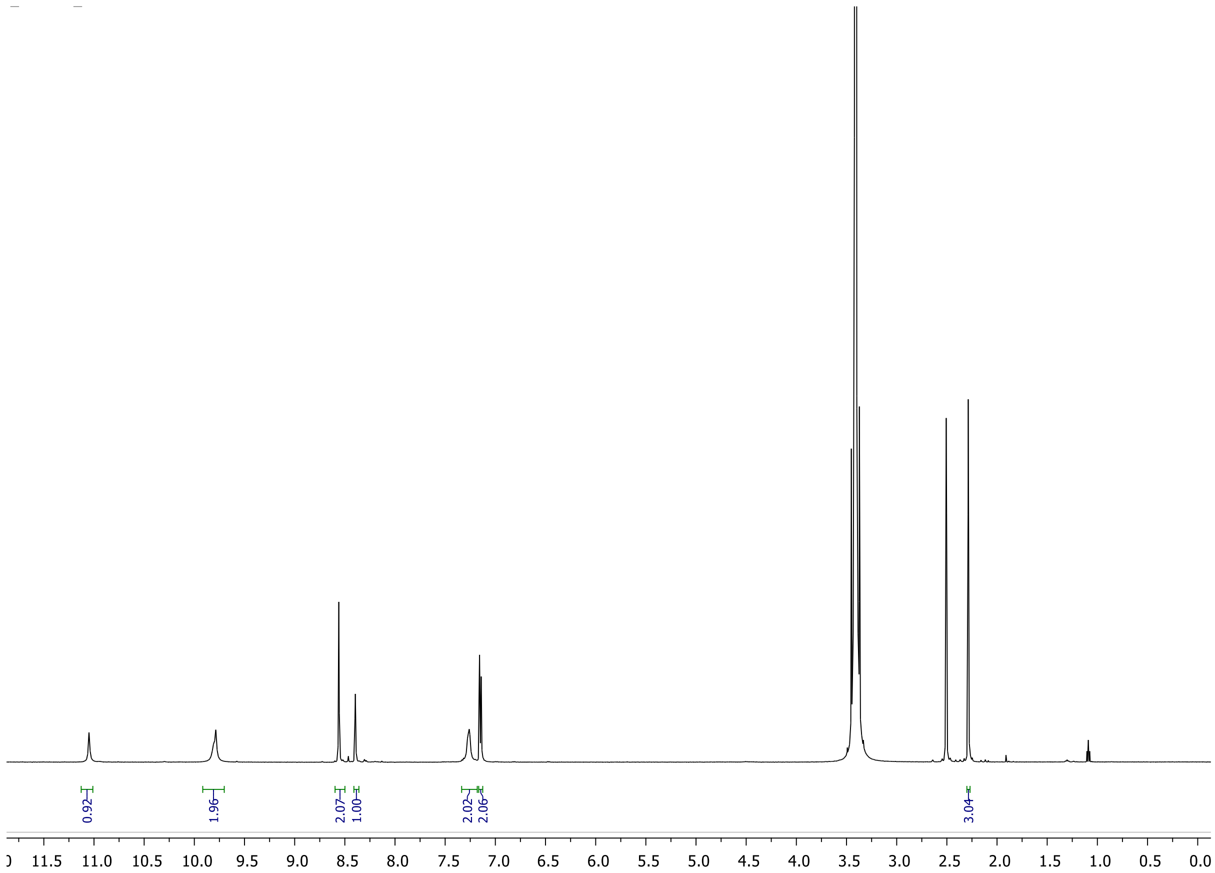

3.20.2 ^{13}C-NMR

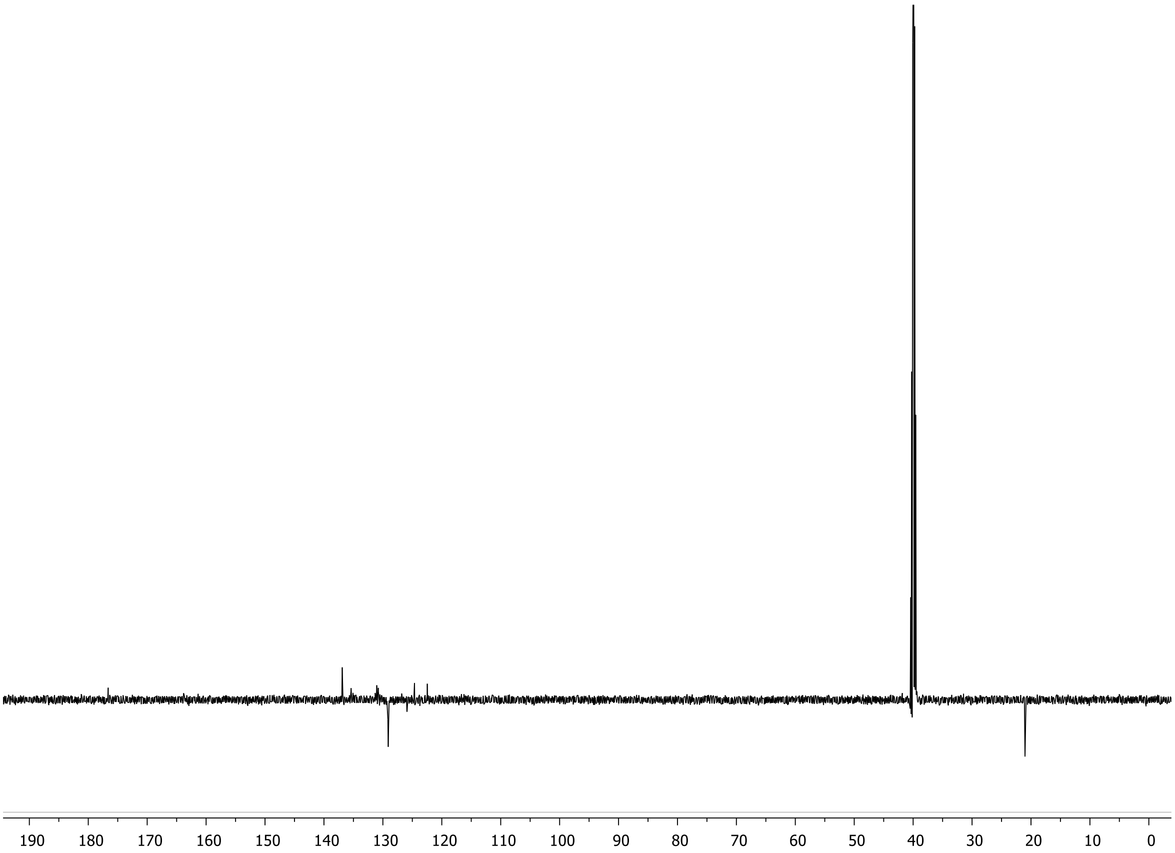

3.20.3 UPLC

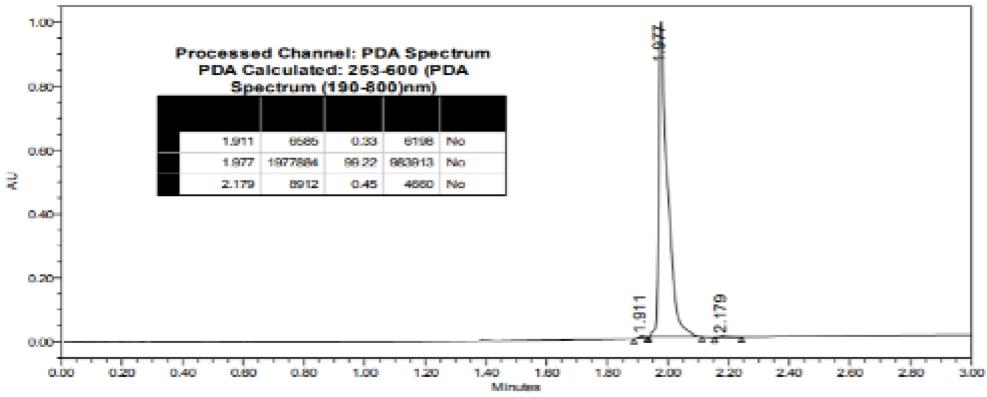
Processed Channel: PDA Spectrum
PDA Calculated: 253-600 (PDA
Spectrum (190-800)nm)
1.911 No
1.977 No
2.179 No
1.977
1.911
2.179
AU
Minutes

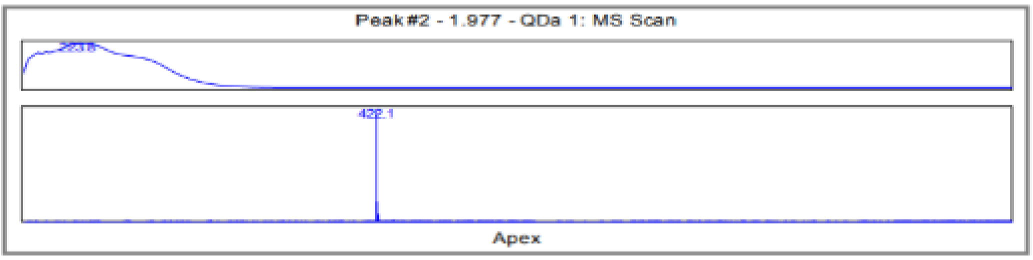
Peak #2 - 1.977 - QDa 1: MS Scan
422.1
Apex

3.21 Compound 74

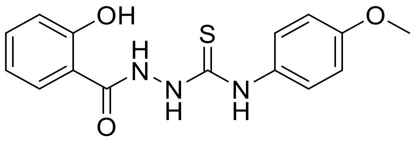

3.21.1 ^{1}H-NMR

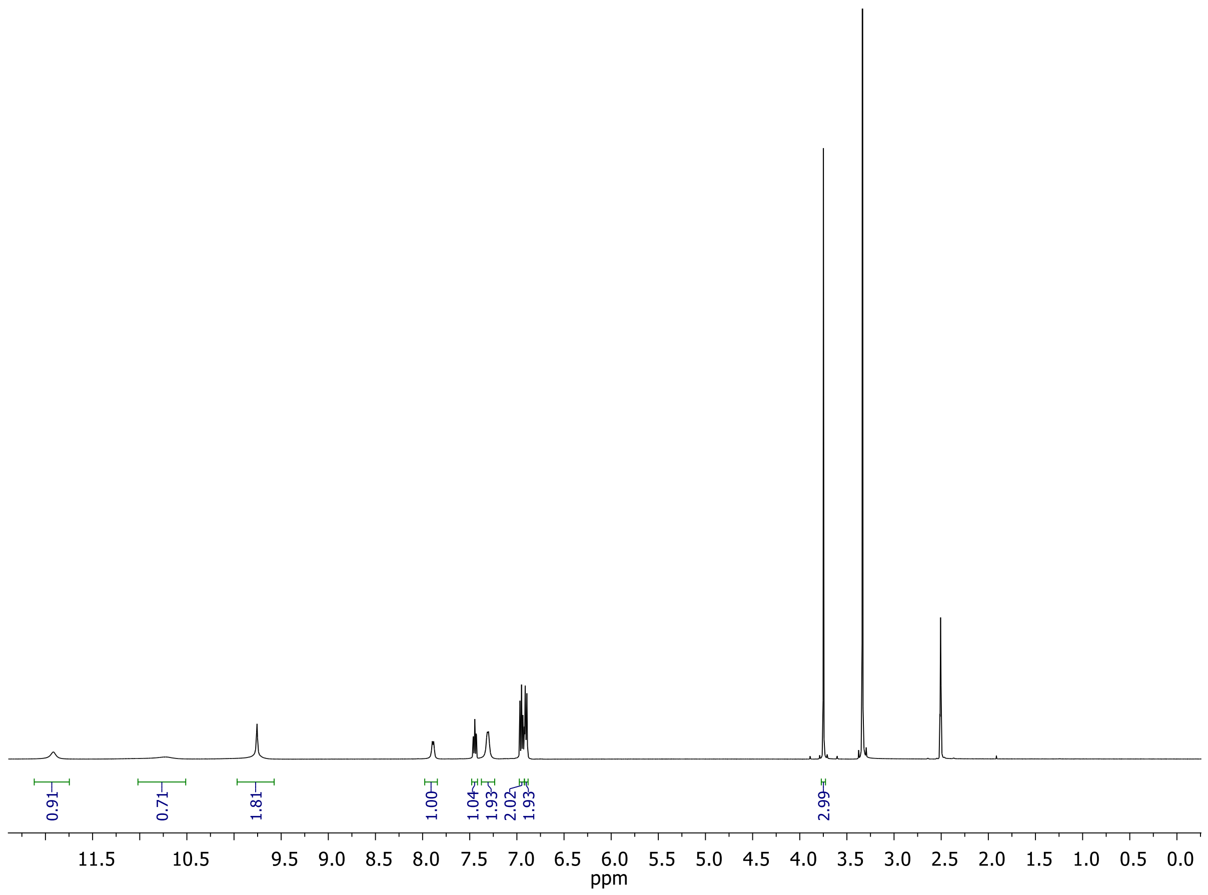

3.21.2 ^{13}C-NMR

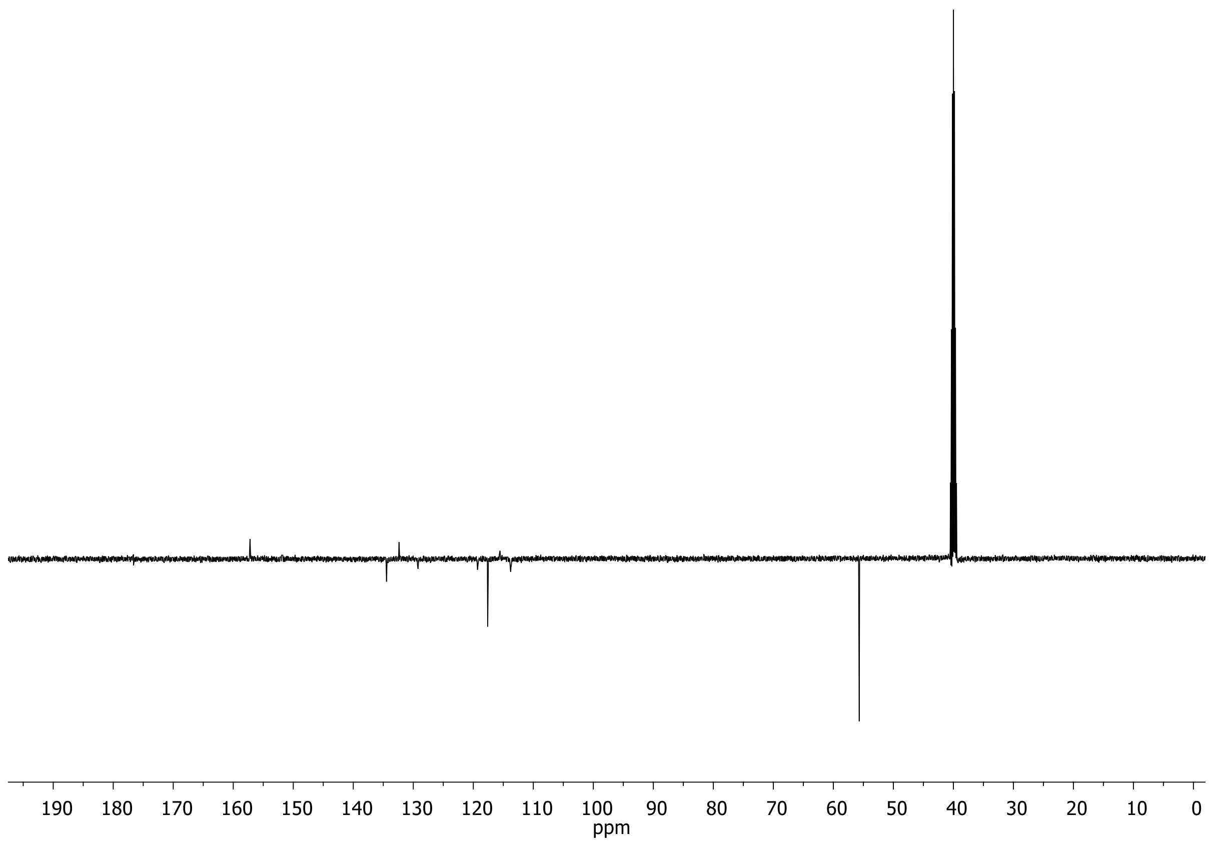
190 180 170 160 150 140 130 120 110 100 90 80 70 60 50 40 30 20 10 0
ppm

3.21.3 UPLC

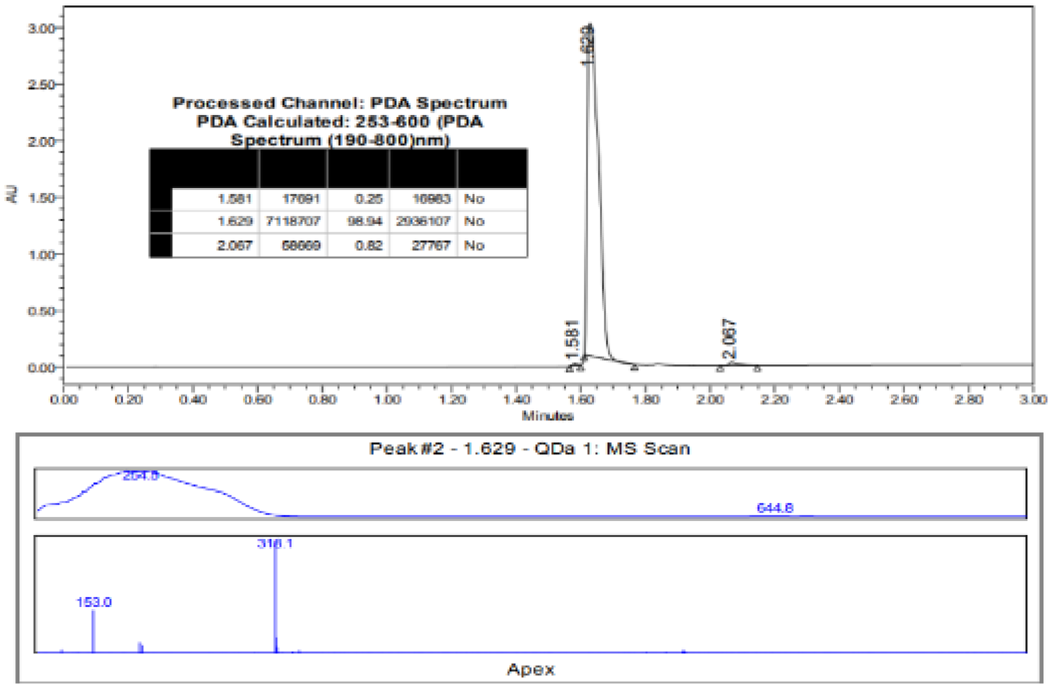
Processed Channel: PDA Spectrum
PDA Calculated: 253-600 (PDA Spectrum (190-800)nm)
1.581 | 17091 | 0.25 | 16963 | No
1.629 | 7118707 | 98.94 | 2936107 | No
2.067 | 58669 | 0.82 | 27767 | No
1.629
1.581
2.067
AU
Minutes
Peak #2 - 1.629 - QDa 1: MS Scan
254.9
644.8
316.1
153.0
Apex

3.22 Compound 75

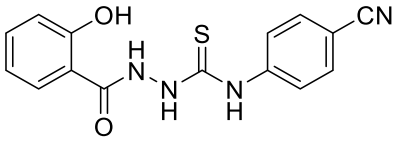

3.22.1 ^{1}H-NMR

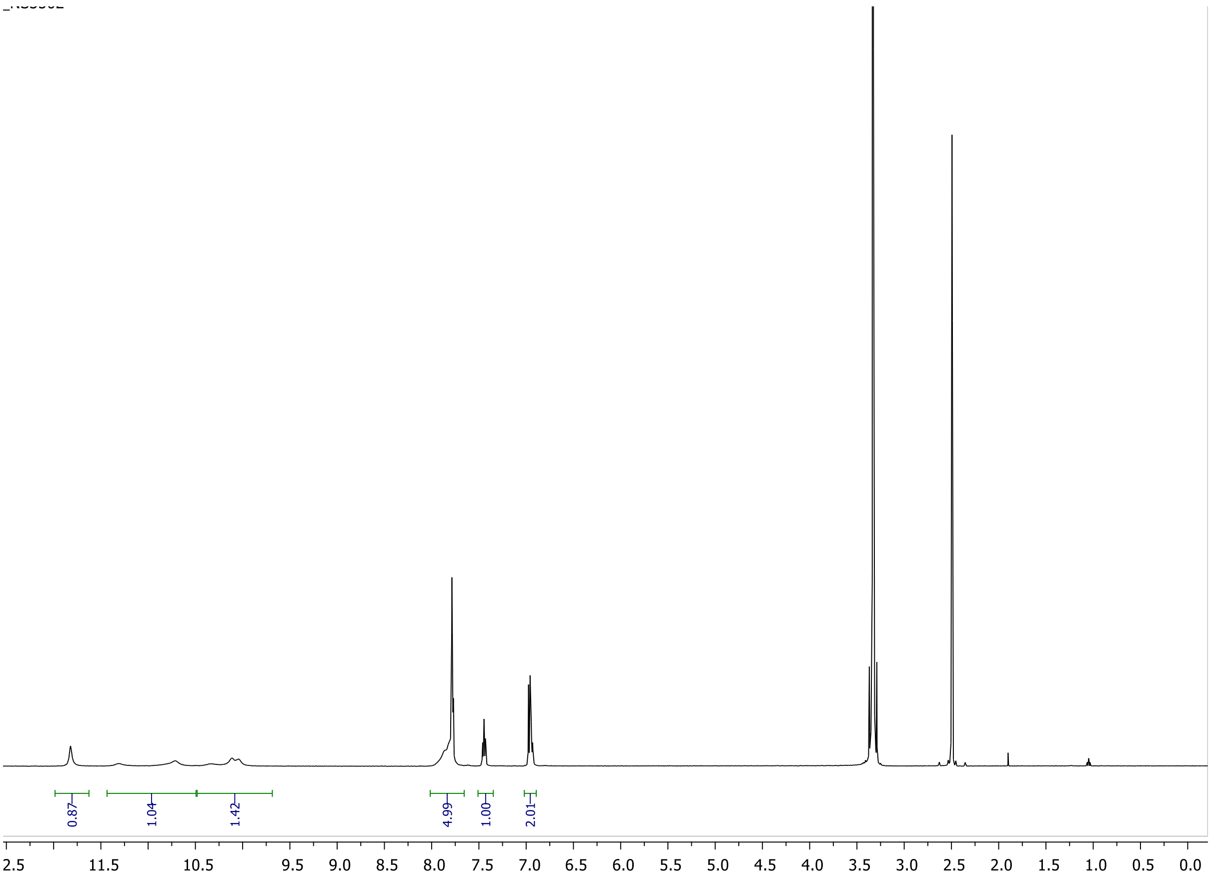

3.22.2 ^{13}C-NMR

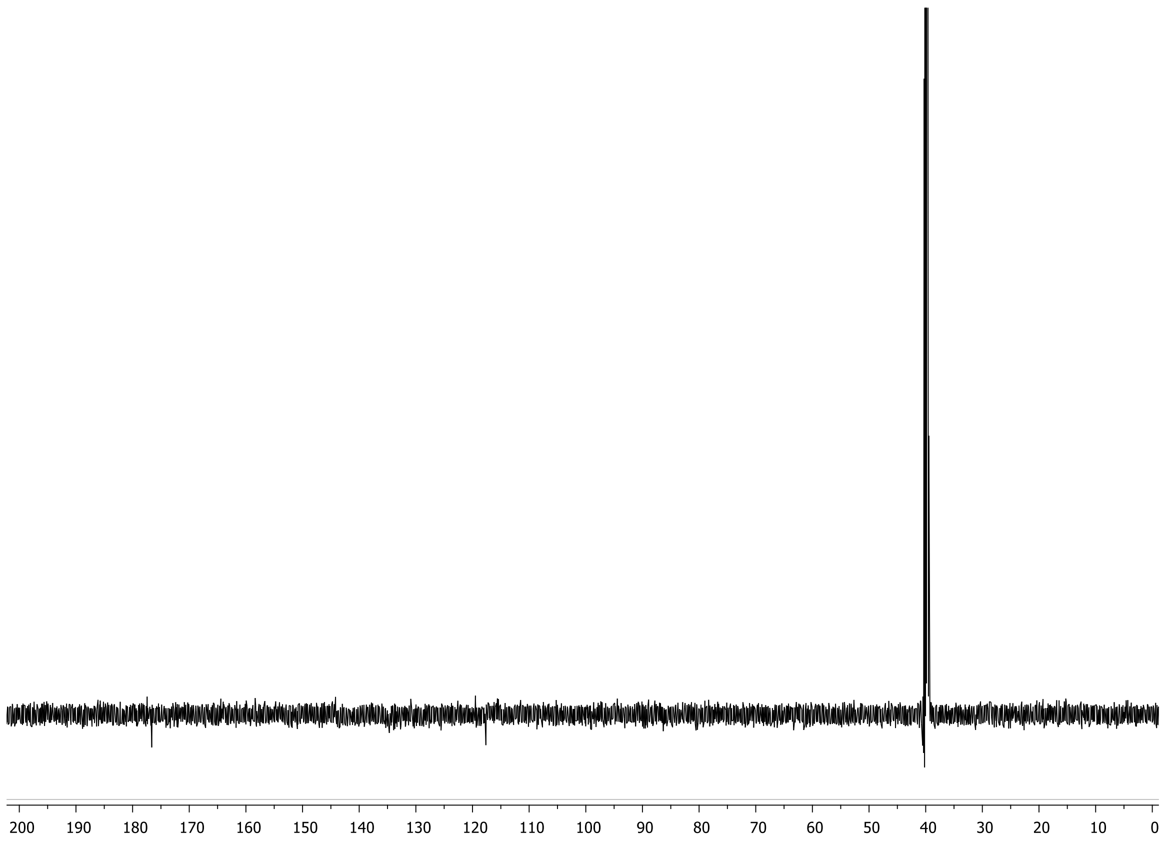

200 190 180 170 160 150 140 130 120 110 100 90 80 70 60 50 40 30 20 10 0

3.22.3 UPLC

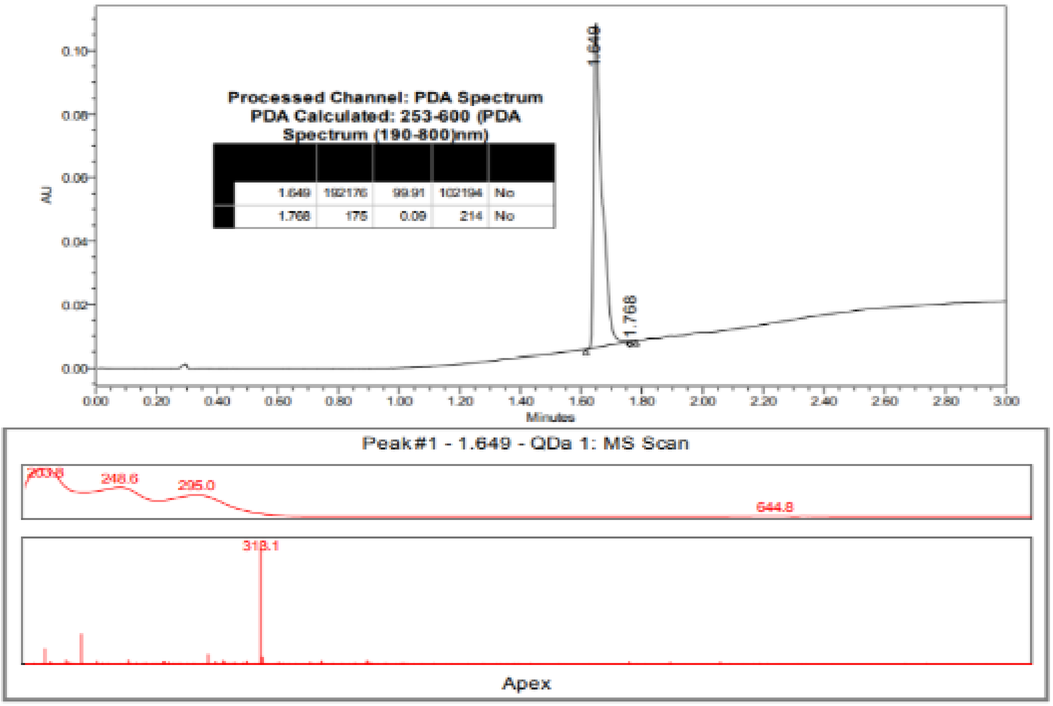
Processed Channel: PDA Spectrum
PDA Calculated: 253-600 (PDA Spectrum (190-800)nm)
1.649 192176 99.91 102194 No
1.768 175 0.09 214 No
1.649
1.768
AU
Minutes
Peak#1 - 1.649 - QDa 1: MS Scan
248.6
295.0
644.8
318.1
Apex

3.23 Compound 76

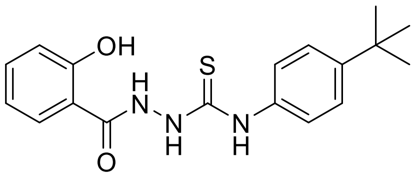

3.23.1 ^{1}H-NMR

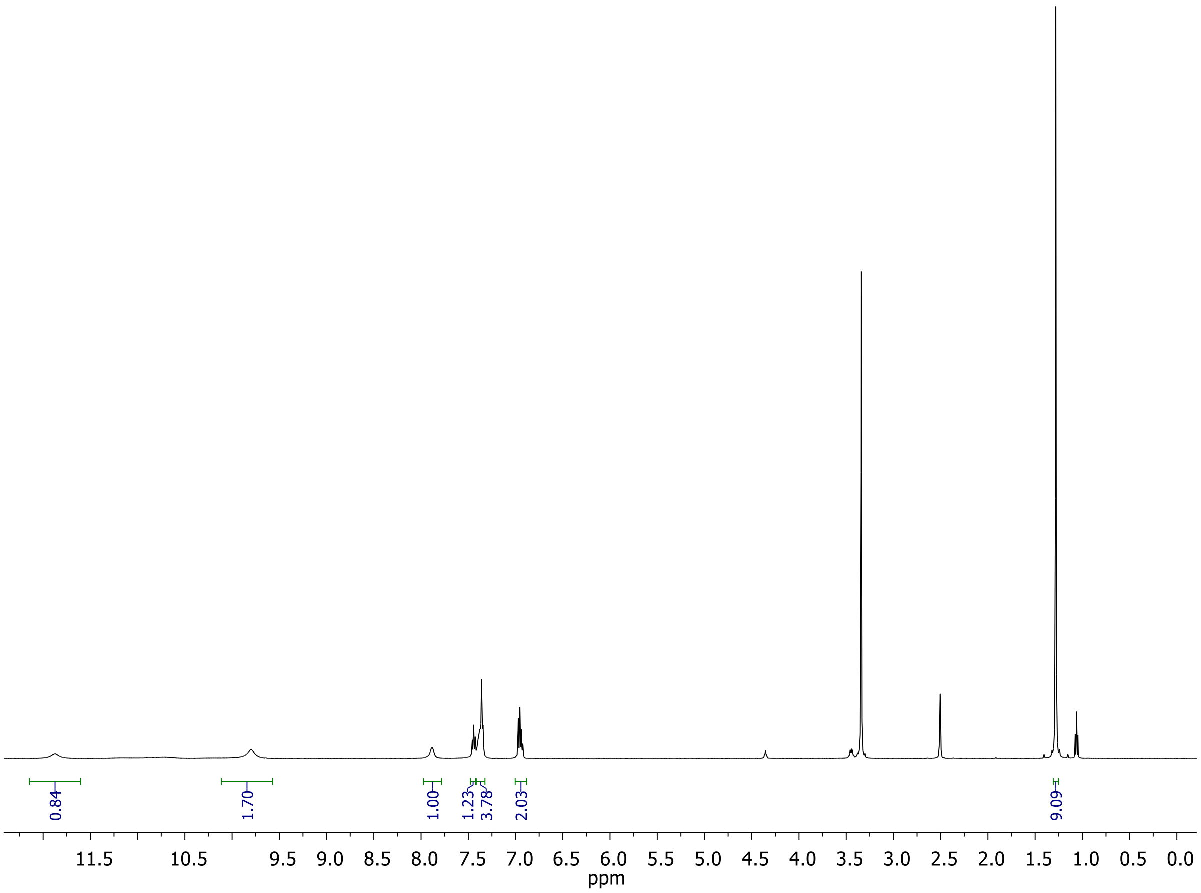

3.23.2 ^{13}C-NMR

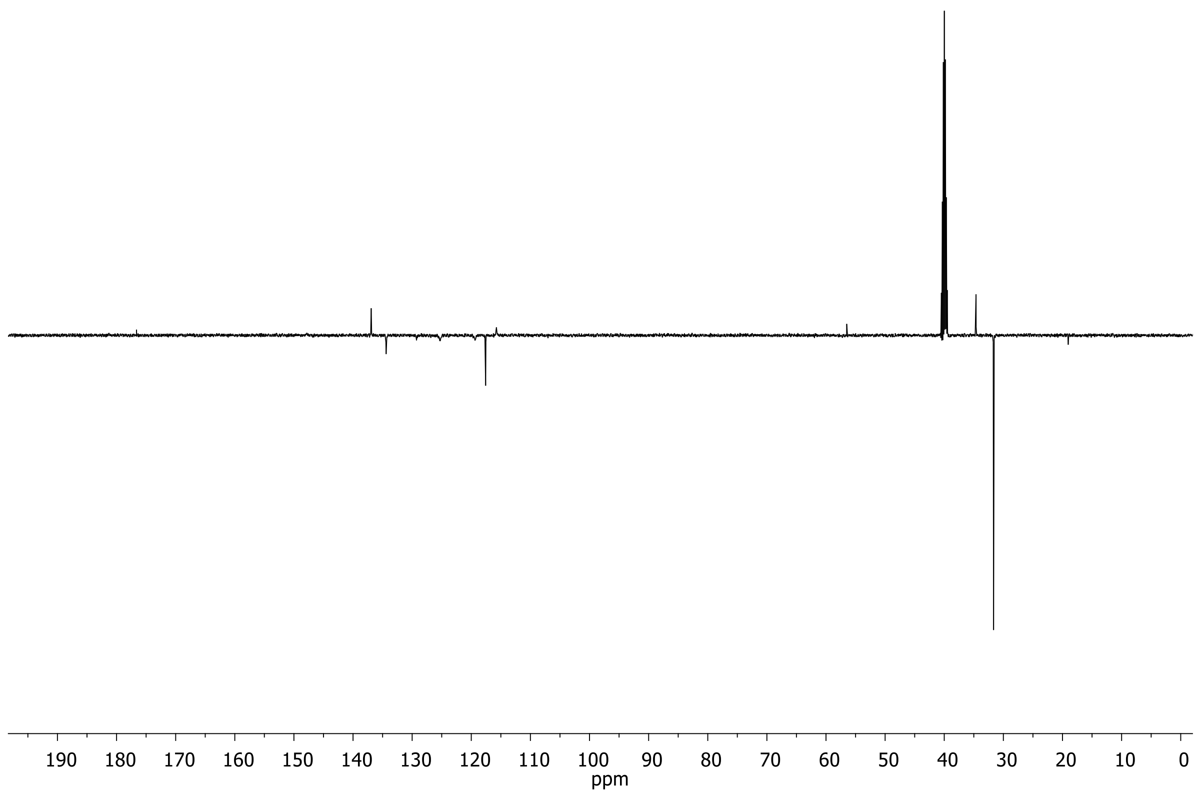
190 180 170 160 150 140 130 120 110 100 90 80 70 60 50 40 30 20 10 0
ppm

3.23.3 UPLC

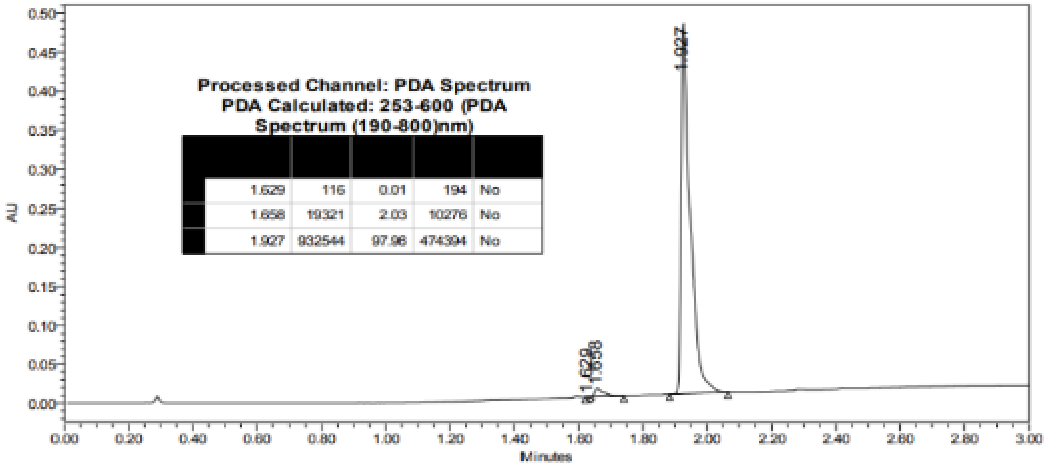
Processed Channel: PDA Spectrum
PDA Calculated: 253-600 (PDA Spectrum (190-800)nm)
1.629 116 0.01 194 No
1.658 19321 2.03 10276 No
1.927 932544 97.98 474394 No
1.927
1.629
1.658
AU
Minutes

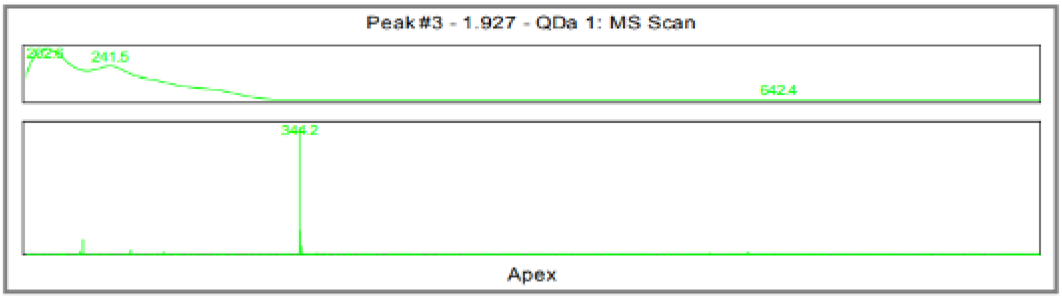
Peak #3 - 1.927 - QDa 1: MS Scan
241.5
642.4
344.2
Apex

3.24 Compound 77

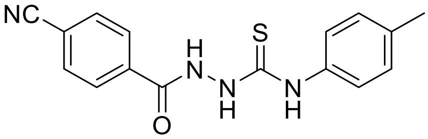

3.24.1 ^{1}H-NMR

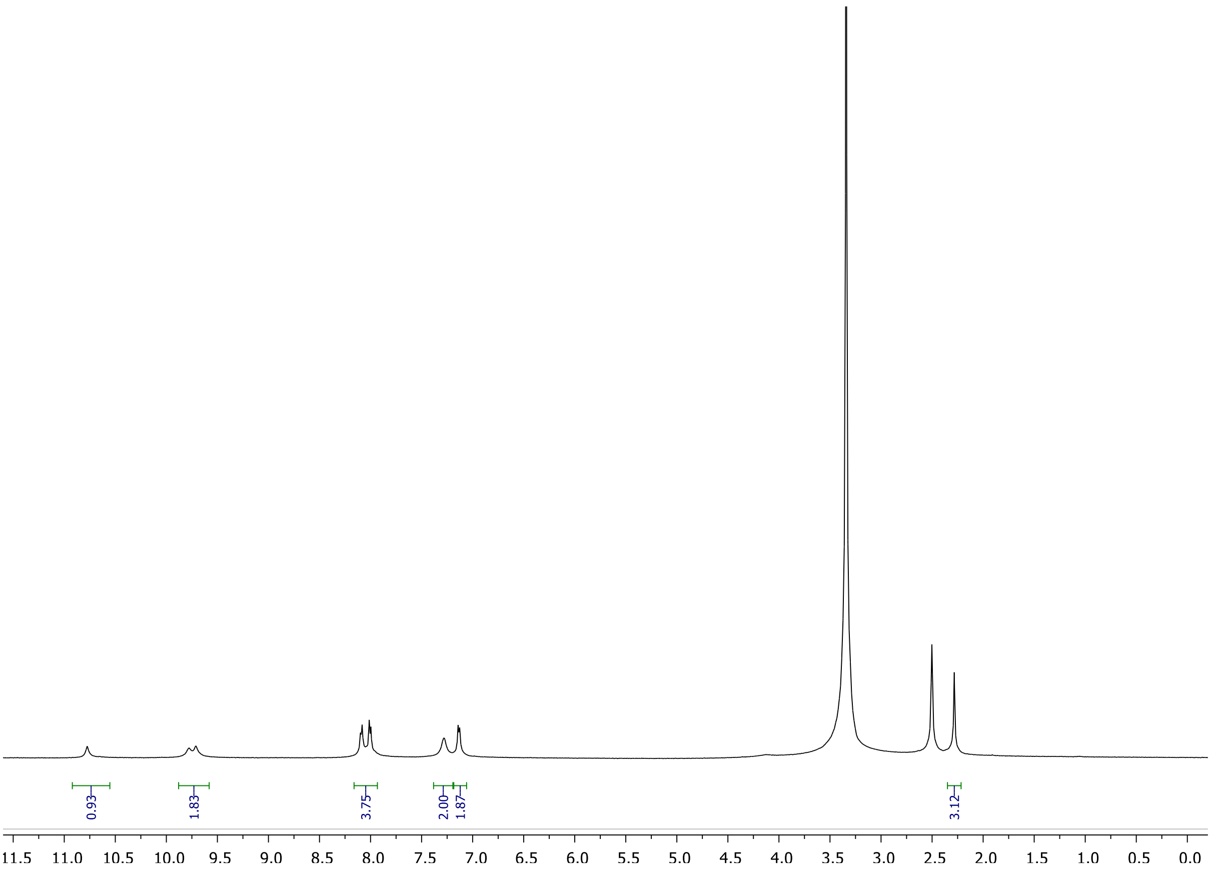

3.24.2 ^{13}C-NMR

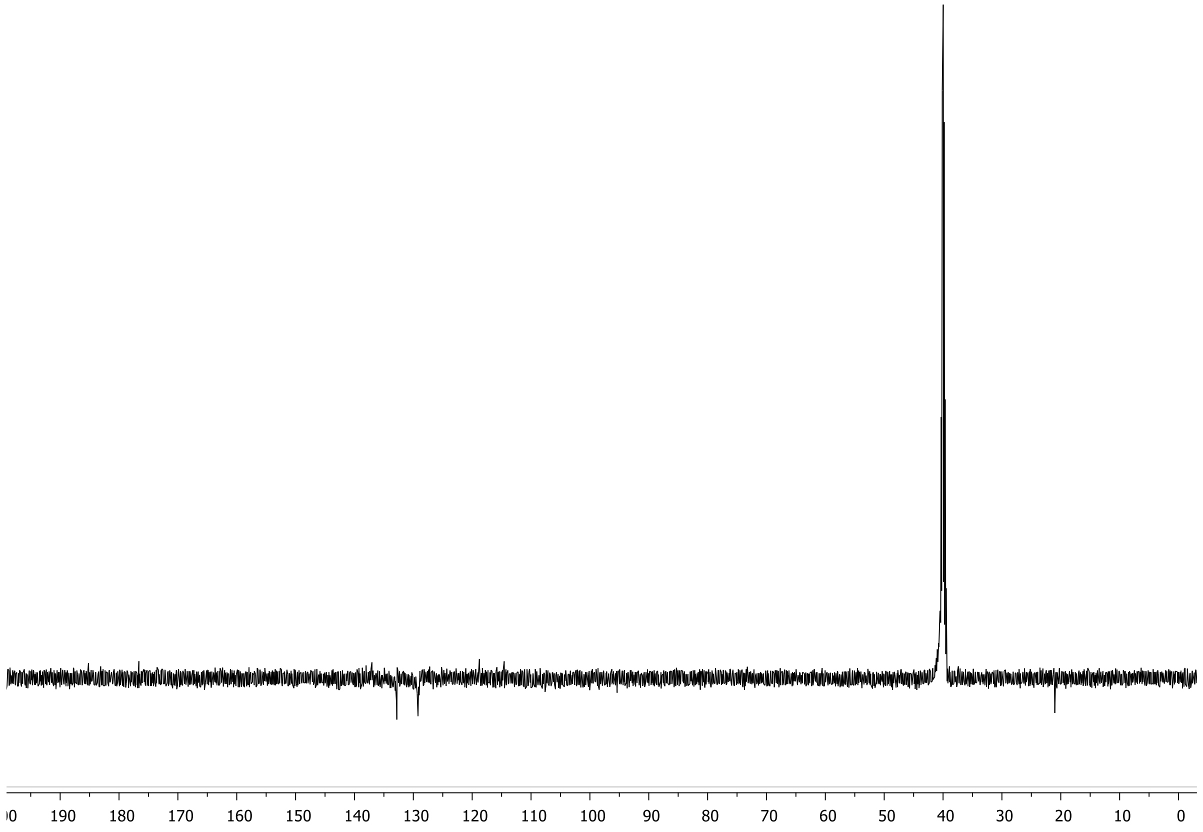

3.24.3 UPLC

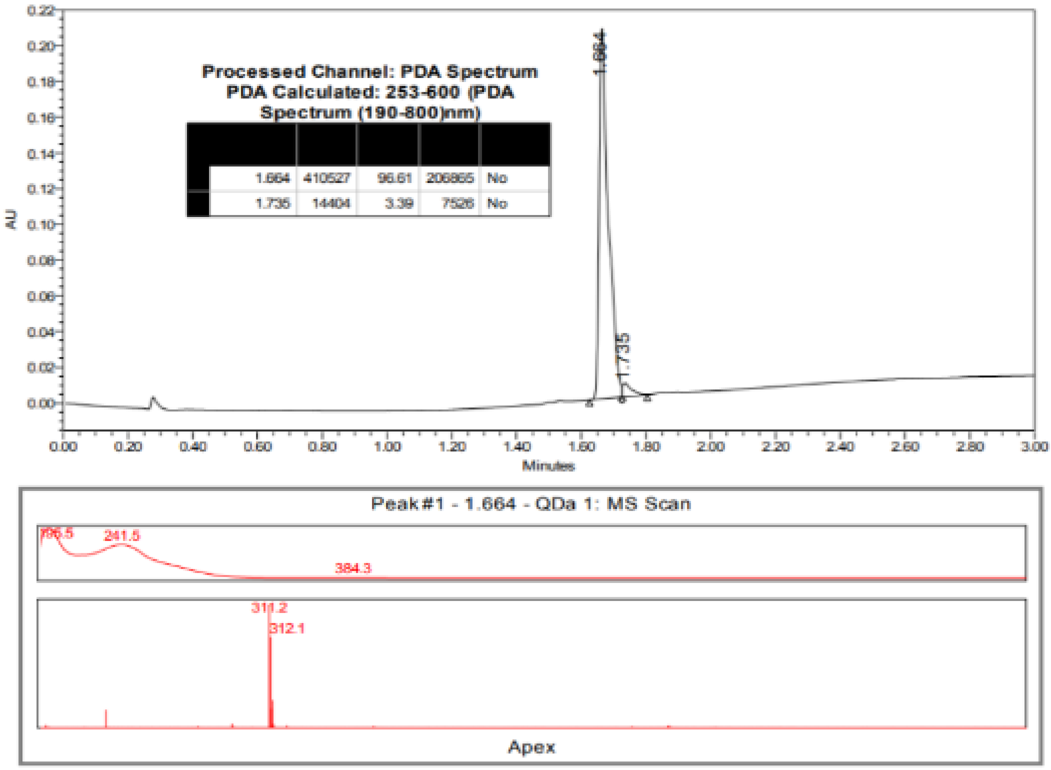
Processed Channel: PDA Spectrum
PDA Calculated: 253-600 (PDA Spectrum (190-800)nm)
1.664 410527 96.61 206865 No
1.735 14404 3.39 7526 No
1.664
1.735
AU
Minutes
Peak #1 - 1.664 - QDa 1: MS Scan
241.5
384.3
311.2
312.1
Apex

3.25 Compound 78

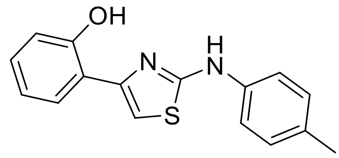

3.25.1 ^{1}H-NMR

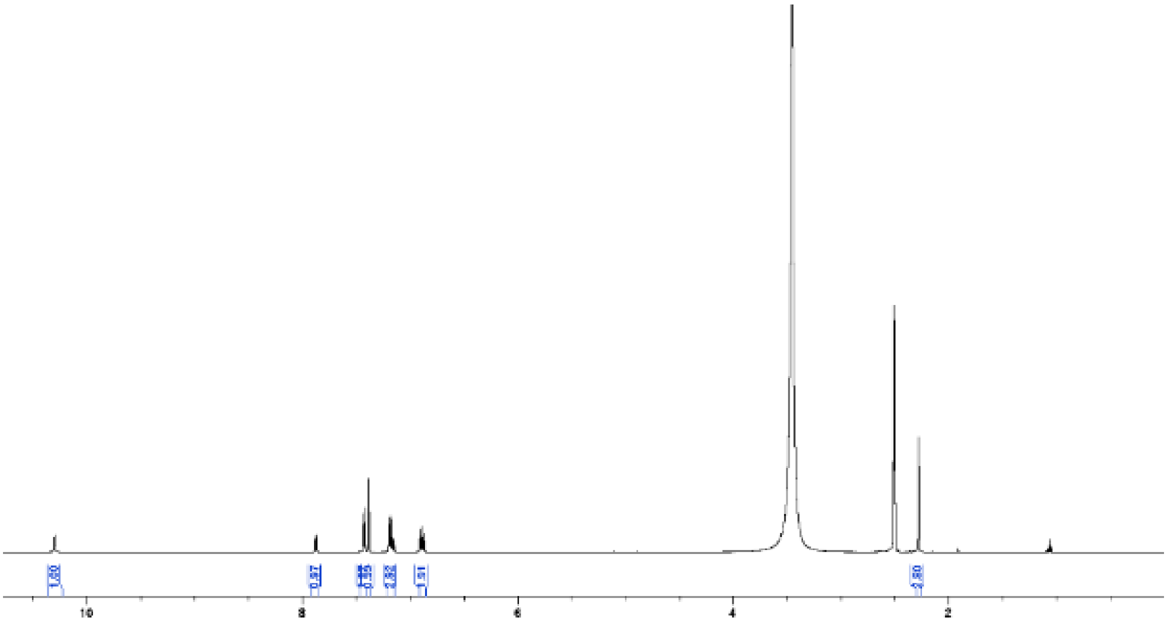

3.25.2 UPLC

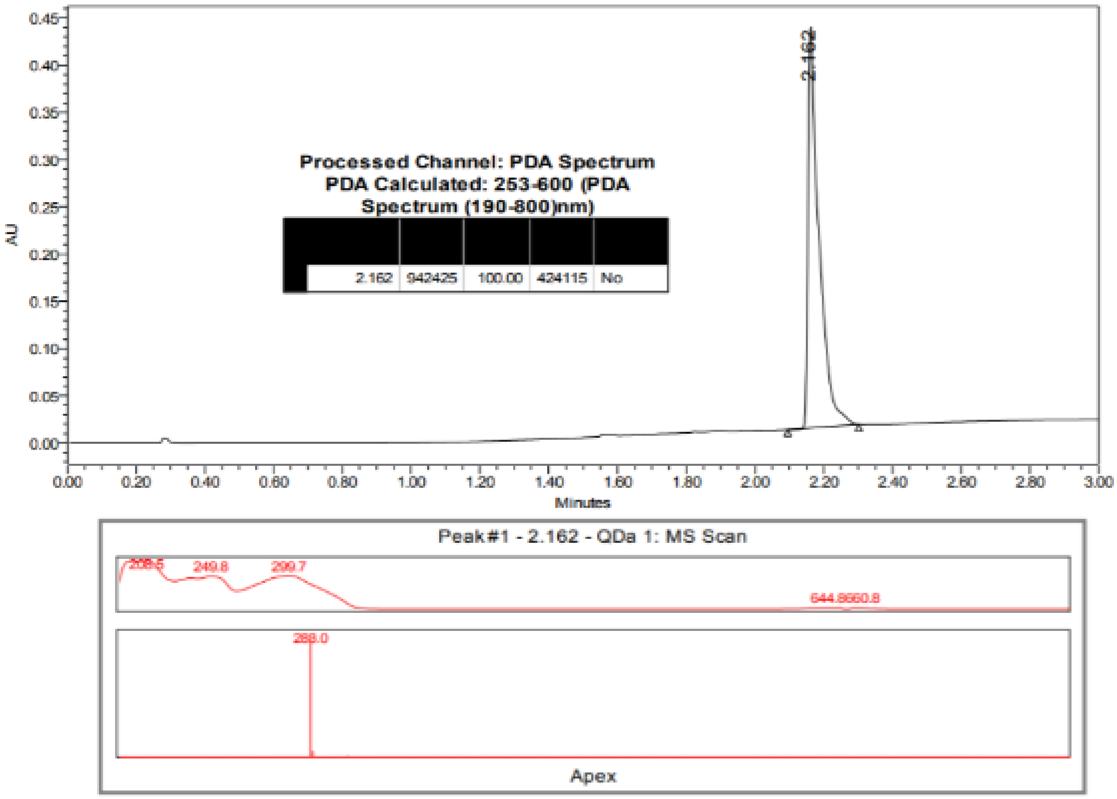

Processed Channel: PDA Spectrum
PDA Calculated: 253-600 (PDA Spectrum (190-800)nm)
2.162
942425
100.00
424115
No
AU
Minutes
Peak#1 - 2.162 - QDa 1: MS Scan
249.8
299.7
Apex

3.26 Compound 79

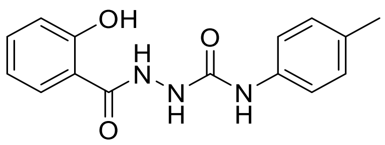

3.26.1 ^{1}H-NMR

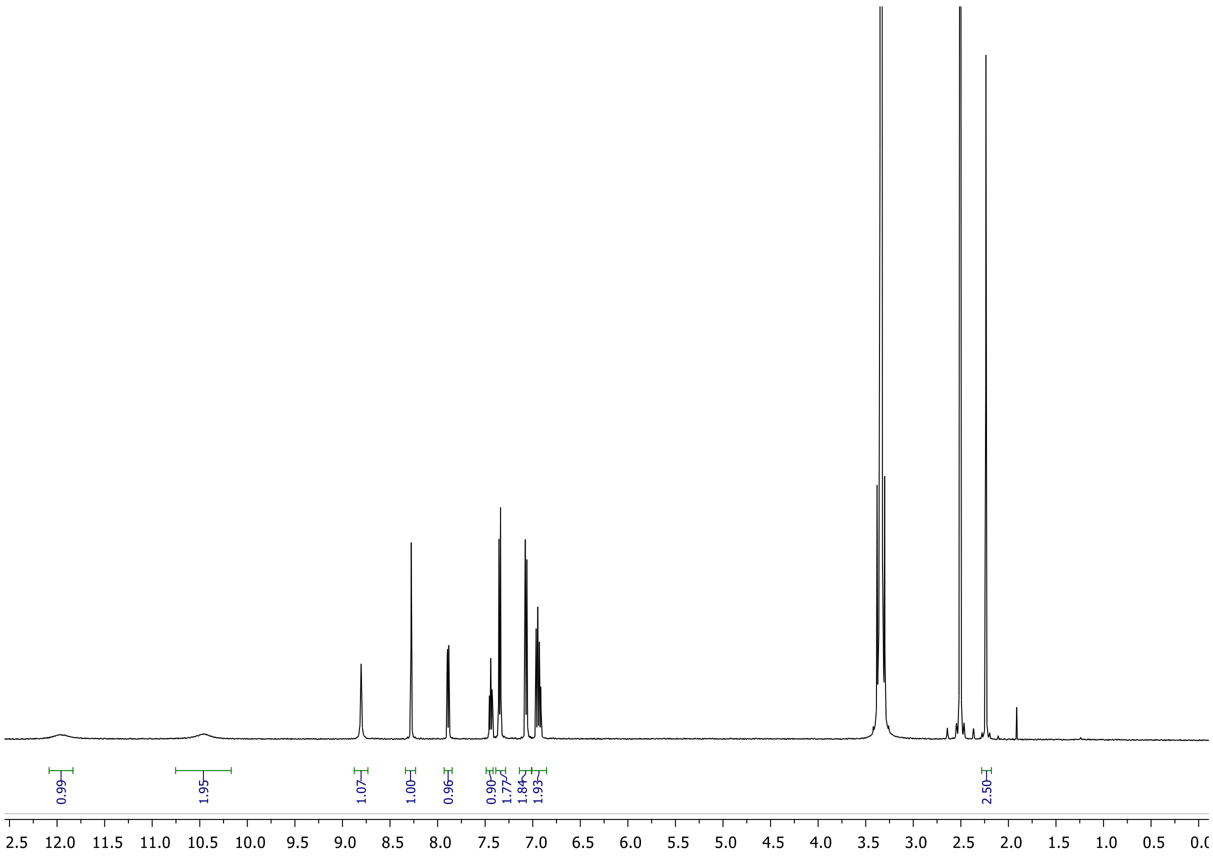

3.26.2 ^{13}C-NMR

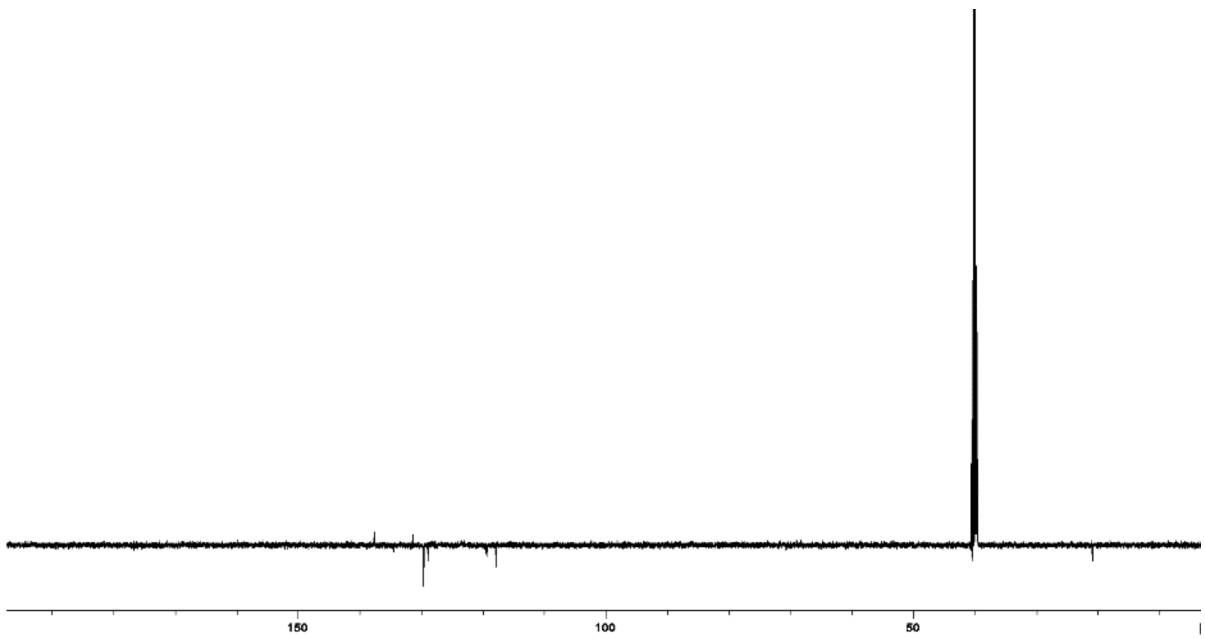

3.26.3 UPLC

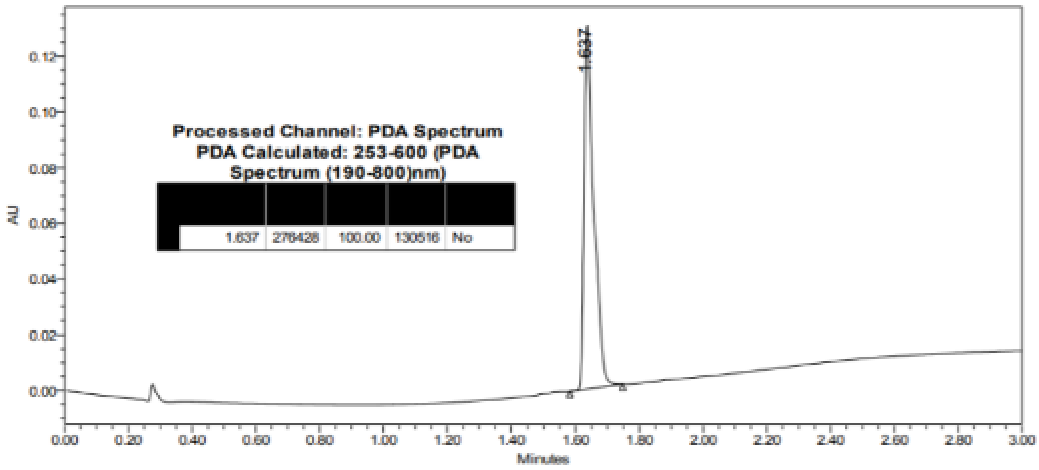

Processed Channel: PDA Spectrum
PDA Calculated: 253-600 (PDA Spectrum (190-800)nm)
1.637 276428 100.00 130516 No
1.637
AU
Minutes

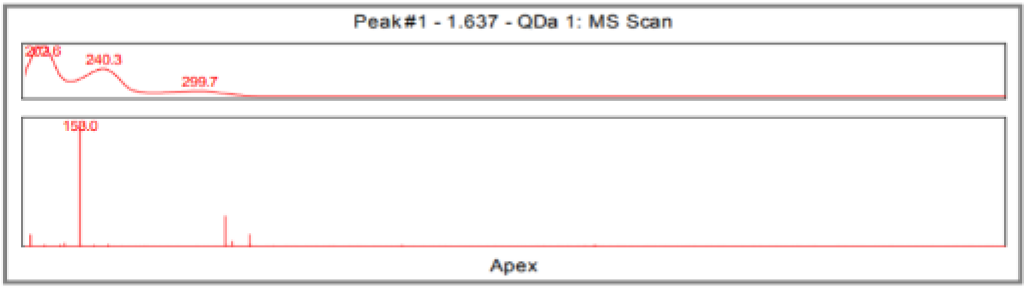

Peak#1 - 1.637 - QDa 1: MS Scan
240.3
299.7
Apex

4. References

Cansiz, A., Etin, A., Kutulay, P., Koparir, M., 2009. Synthesis of Tautomeric Forms of 5-(2-Hydroxyphenyl)- 4-substituted-3H-1,2,4-triazole-3-thione). *Asian Journal of Chemistry,* 21, 617-626.
